# New Generation Modified Azole Antifungals against Multidrug-Resistant 

**DOI:** 10.1021/acs.jmedchem.5c01253

**Published:** 2025-06-23

**Authors:** Yiyuan Chen, Yunxiao Li, Kazi S. Nahar, Md. Mahbub Hasan, Caleb Marsh, Melanie Clifford, Godwin A. Aleku, Steven L. Kelly, David C. Lamb, Chengetai Diana Mpamhanga, Ilias Kounatidis, Ajit J. Shah, Charlotte K. Hind, J. Mark Sutton, Khondaker Miraz Rahman

**Affiliations:** † Institute of Pharmaceutical Science, 4616 King’s College London, 150 Stamford Street, London SE1 9NH, United Kingdom; ‡ Countermeasures, Development, Evaluation and Preparedness, UK Health Security Agency, Manor Farm Road, Salisbury SP4 0JG, United Kingdom; § Department of Natural Sciences, University of Middlesex, The Burroughs, Hendon, London NW4 4BT, United Kingdom; ∥ Department of Genetic Engineering and Biotechnology, Faculty of Biological Sciences, University of Chittagong, Chattogram 4331, Bangladesh; ⊥ Centre for Cytochrome P450 Biodiversity, Faculty of Medicine, Health and Life Science, Swansea University, Swansea, SA2 8PP, United Kingdom; # School of Life Health and Chemical Sciences, 5488 The Open University, Milton Keynes MK7 6AE, United Kingdom

## Abstract

The rise of antifungal resistance and limited treatment options highlight the urgent need for new drug classes. is a serious global health threat with few effective therapies. In this study, novel azole-based compounds were developed by modifying the azole core with cyclic heteroaliphatic linkers connecting aromatic and heteroaromatic rings. Several compounds showed potent activity against , including azole-resistant strains, with MICs ranging from 0.016 to 4 μg/mL. The compounds also demonstrated strong activity against , , , and , with MICs mostly below 1 μg/mL. Compounds **7**, **18**, and **21** were more potent than fluconazole. Compound **7** inhibited CYP51, eradicated biofilms, and showed better intracellular accumulation than fluconazole. *In vivo* studies in and confirmed efficacy at 5 mg/kg and no toxicity up to 50 mg/kg, supporting further development of this scaffold against multidrug-resistant infections.

## Graphical abstract

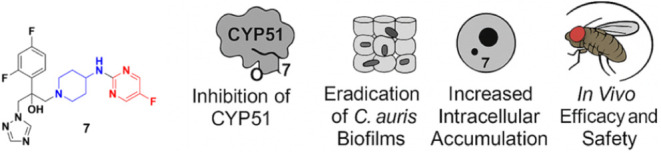

## Introduction

Antifungal resistance is the ability of a fungus to grow and survive in the presence of antifungal drugs. This can lead to severe infections that are hard to treat.^1,2^ Antifungal drugs are used to treat a variety of fungal infections, including candidiasis, aspergillosis, and cryptococcosis.^3–5^ Resistance can occur naturally or develop over time due to exposure to antifungal drugs or fungicides. Improper use of antifungal drugs, such as low doses or short courses, can also contribute to resistance.^2,6,7^ Some fungi, like and certain species, are resistant to some or all types of antifungal drugs.^8–11^ Among them, is a new and highly resistant fungus that can spread quickly in healthcare settings.^12–14^

 is an emerging fungal pathogen that is resistant to many antifungal drugs. It can cause serious infections in hospitals and other healthcare settings.^15–18^ The U.S. Centers for Disease Control and Prevention (CDC) and the World Health Organization (WHO) both consider to be a major threat to public health.^19^ It was first discovered in Japan in 2009 and has since been reported in over 47 countries worldwide, with 6 clades emerging.^20^ A study of patients with the echinocandin-susceptible bloodstream infection at three hospitals in Brooklyn, New York, found that 30.1% of patients died within 30 days and 44.6% died within 90 days.^21^

Resistant strains are relatively prevalent in clinical settings. The collected data from the CDC showed that around 7% of the clinical strains isolated from hospitalized patients suffering from bloodstream infections exhibited resistance to marketed antifungal drugs.^22,23^ In another study, approximately 90% of the isolates were found to be resistant to at least one commercially available antifungal drug, and 30% of the clinical strains were nonsusceptible to more than one antifungal on the market.^24^ All the data from public health authorities make it clear that innovation in the treatment of antifungal infections, particularly drug-resistant , is urgently necessary.

There are only four major classes of antifungal drugs available on the market for treating systemic fungal infections, namely azoles (e.g., fluconazole, voriconazole, itraconazole, and posaconazole), polyenes (typically amphotericin B), echinocandins (e.g., micafungin and caspofungin), and pyrimidine analogues (mainly flucytosine or its salt form).^25,26^ Azoles (imidazoles and triazoles, containing two and three nitrogens on the azole ring, respectively) are a class of antifungal drugs widely used to treat fungal infections in humans.^27^ They work by inhibiting the synthesis of ergosterol, an essential component of fungal cell membranes. This disruption weakens the cell membrane and leads to the death of the fungal pathogen.^28,29^

Despite the potential of azoles, the increasing levels of resistance against them are becoming a concern in tackling the healthcare burden caused by fungal infections. The treatment of fungal pathogens using azoles triggers the upregulation of efflux pumps in *Candida spp.*, including CDR1, MDR1, RDC3, SNQ2, and YHD3 in .^30–32^ Moreover, reports show that . can also overexpress azole targets such as the ERG11 gene (that encodes lanosterol 14-alpha-demethylase),^33,34^ sequester azoles within vacuoles and biofilms (in fluconazole-resistant ),^35,36^ and modify the targets by mutation over time (ERG11 mutation triggered by the application of azoles in .).^37,38^ To develop azoles that can overcome resistance observed in and other drug-resistant species, we made a targeted modification in the azole core scaffold with various types of heteroaliphatic linkers with different lengths, sizes, and ring strains (Figure 1A) and modified the terminal aromatic and heteroaromatic ring. This medicinal chemistry-led approach has led to compounds with activity against multidrug-resistant and other clinically important species that can be taken forward toward preclinical development.

**Figure 1:**
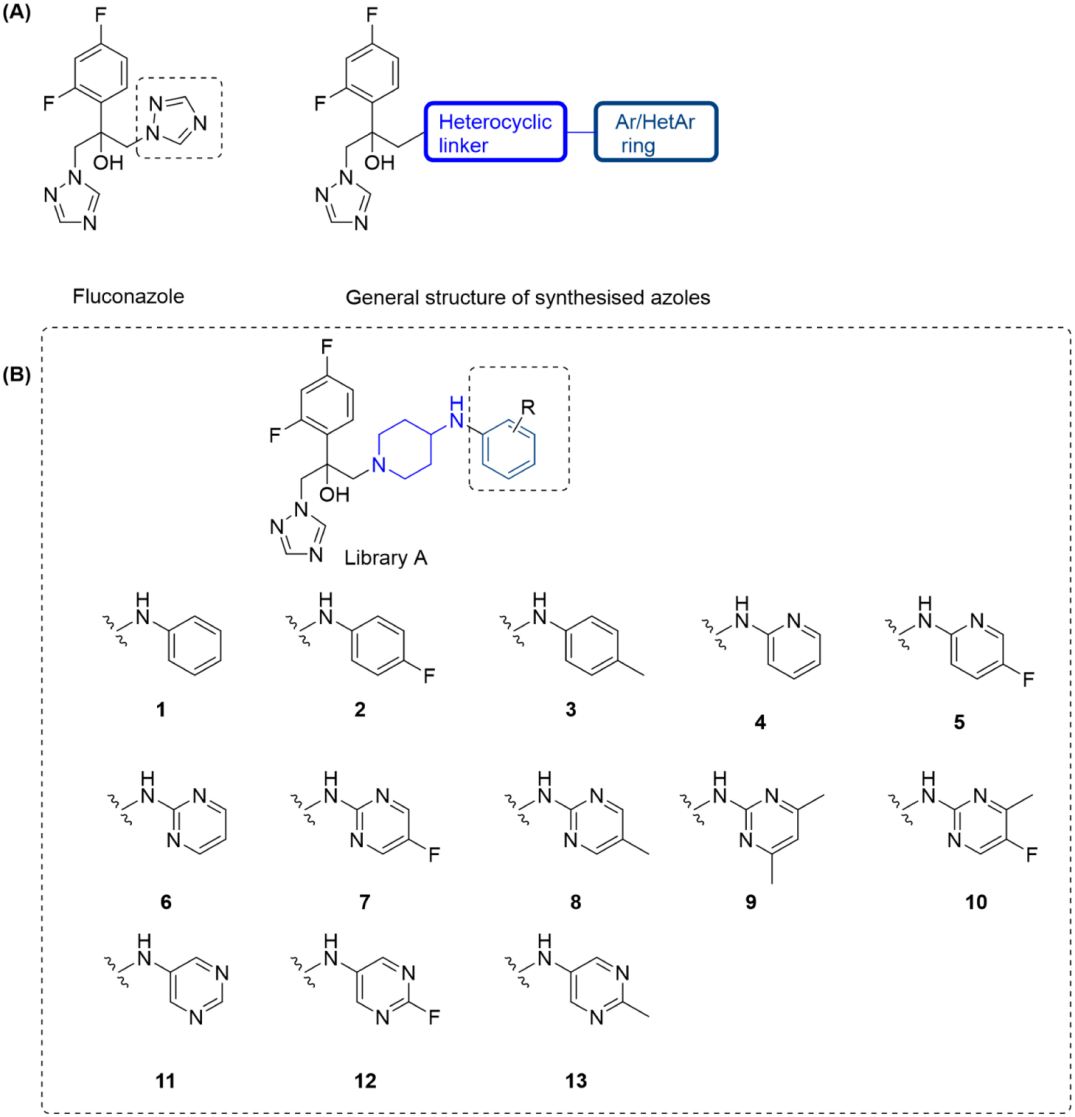
Chemical structures of fluconazole and modified azole compounds. (A) General structure of the modified azole compounds. (B) Compounds of library A containing a piperidine linker and different aromatic or heteroaromatic rings.

## Results and Discussion

### Design of Modified Azole Compounds

We aimed to design fluconazole analogues with improved interaction with the fungal lanosterol 14α-demethylase (LDM), which is the target for azole antifungals and is involved in the biosynthesis of ergosterol.^39^ For all compounds, one of the triazole rings of fluconazole was replaced by a heteroaliphatic linker connected to an aromatic or heteroaromatic ring (Figure 1A). For library A compounds, we designed compounds with a six-membered heterocyclic piperidine ring as the linker as it has previously been used to generate azole modifications, and compounds with this linker have shown MICs comparable to fluconazole.^40,41^ The piperidine ring was substituted at the 4-position with an amino group, which was considered as a point of diversity and was substituted with a number of six-membered aromatic and heteroaromatic rings to establish the structure–activity relationship (Figure 1B).

Designed compounds having phenyl, pyridine, and pyrimidine as terminal aromatic or heteroaromatic fragments were then utilized for the computational analysis against the target LDM from different species along with the clinically relevant drugs fluconazole and voriconazole (Table 1). The data from the computational analysis suggested that the newly designed modified fluconazole compounds showed better binding affinity to the LDM enzyme from both and compared to both fluconazole and voriconazole. In terms of both ChemScore and Gibbs free energy Δ*G*, most of the modified azole compounds showed almost twice the binding affinity compared to fluconazole. The results of the binding affinity remained similar across both species of (Table 1).

**Table 1:** Comparative Binding Affinity of Novel Azole Analogues and Commercially Available Azoles with Fungal Target Lanosterol 14α-Demethylase (LDM)

	calLDM (PDB ID 5TZ1)		carLDM (A0A2H4QC40)	
Compound	Chem Score	Δ*G* (kcal/mol)	Chem Score	Δ*G* (kcal/mol)
Fluconazole	18.16	–18.94	16.07	–18.8
Voriconazole	24.6	–26.72	21.03	–26.48
1	40.46	–42.6	36.16	–39.65
2	41.22	–42.69	37.32	–38.34
3	36.3	–37.52	32.25	–33.7
4	34.39	–37.4	33.68	–34.13
5	31.77	–33.34	30.77	–33.33
6	32.88	–35.63	30.96	–32.1
7	41.22	–42.69	37.32	–38.34
8	39.45	–41.6	35.6	–34.54
9	40.8	–37.6	37.6	–37.4
10	38.6	–36.54	38.45	–38.33
11	34.31	–35.65	30.44	–33.61
12	33.45	–33.66	31.32	–34.7
13	35.74	–37.4	33.22	–35.6

### Synthesis of Library A

This library consisted of 13 compounds in a two-step process; the fragments were synthesized or purchased (compounds **2b**, **4b**–**7b, 11b**) and then connected to the azole core by an epoxide ring-opening reaction to form the final compounds. Fragments were synthesized by following three different approaches based on the electronic environment of the connecting carbon (Scheme 1).

**Scheme 1:**
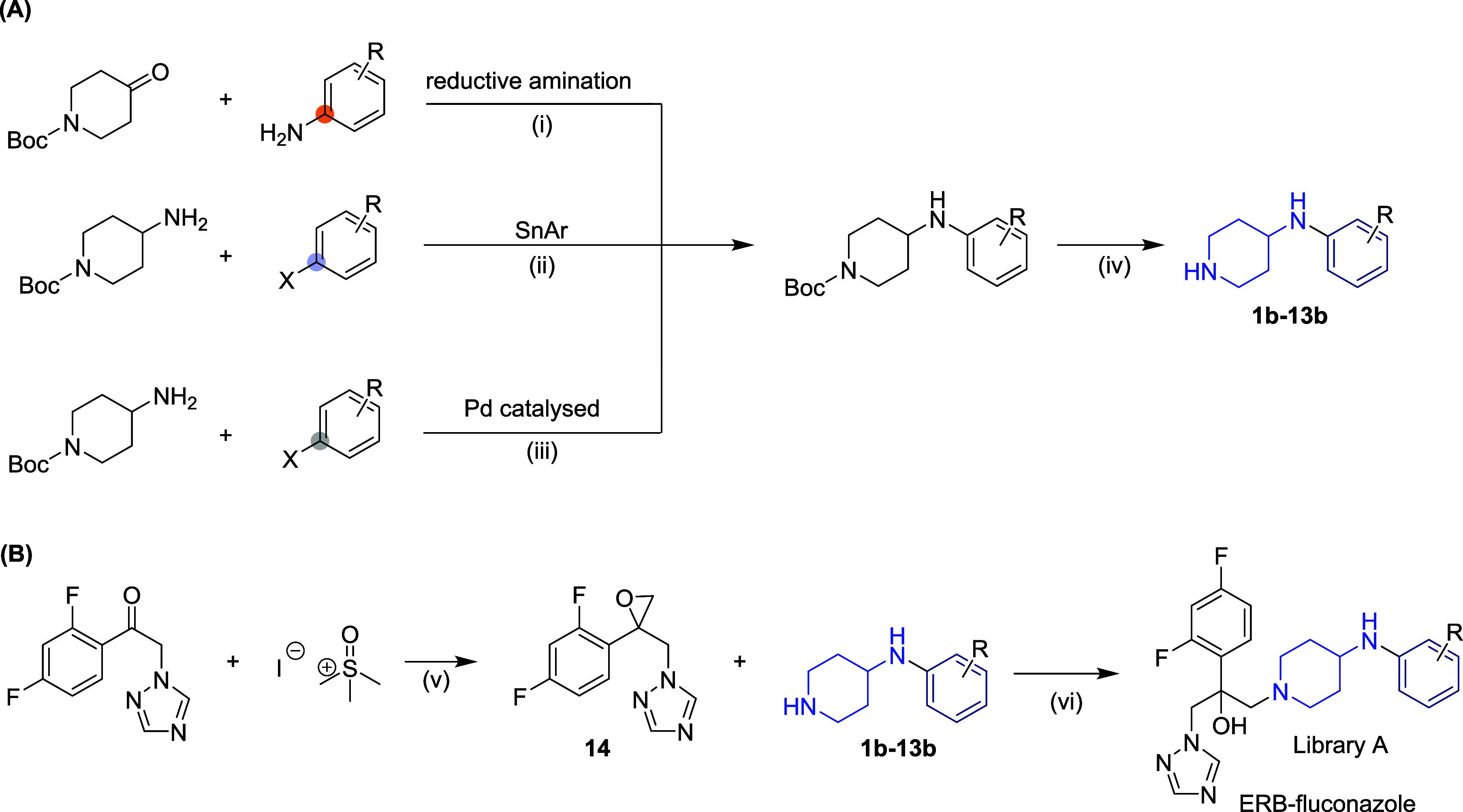
General Synthetic Routes for Library A where Different Aromatic/Heteroaromatic Fragments (A) were Connected to the Fluconazole Core via a Piperidine Linker (B). Conditions: (i) NaBH­(OAc)­3, AcOH, DCM/MeOH, Overnight; (ii) DIPEA, MeCN, DMA, 160–180 °C, 2–3 h or TEA, Ethylene Glycol, 200 °C, 15 min; (iii) rac-BINAP, Pd2­(dba)­3, KOtBu, Toluene, 100 °C, Overnight; (iv) 4 M HCl in 4-Dioxane, r.t., 1–2 h; (v) NaOH (aq.), Toluene, 80 °C, Microwave 50 min; (vi) TEA, EtOH, 80 °C, Overnight

The fragments were synthesized either by reductive amination (**1a, 2a, 12a,** and **13a**) or by simple SNAr reactions depending on the electronic environment of the terminal aromatic/heteroaromatic ring (Scheme 1A). In the case of fragment **10a**, a palladium-catalyzed organometallic reaction was carried out to obtain the fragment. The epoxide core of the azole compounds was synthesized using the classic Corey–Chaykovsky reaction conditions (Scheme 1B). The final compounds were synthesized by reacting the amine fragments **1b**–**13b** with the epoxide core 14 with a nucleophilic epoxide ring-opening reaction. This resulted in racemic final compounds **1** to **13,** which were evaluated for their antifungal activity (Scheme 1).^42,43^

### Antifungal Activity of Library A

All 13 compounds were tested against a panel of 4 strains from different clinically important species and an extended panel of (8 strains), and their antifungal activity and lipophilicity were compared against the reference compound, fluconazole (Table 2). The panel covers a range of azole susceptibility profiles and clades (Clade I; TDG2211, NCPF8971, Presumptive Clade I; TDG2512, Clade II; TDG2506, NCPF8984, Clade III; TDG1102, NCPF8977, and TDG1912). MICs were determined in accordance with the EUCAST guidelines, which specify that for fungistatic agents such as azoles, the MIC is defined as the lowest concentration that inhibits ≥50% of growth compared to the drug-free control. While there is no defined EUCAST or CLSI resistance breakpoint for fluconazole in , there is a presumed breakpoint of 32 μg/mL; therefore, the panel contains both azole “susceptible” and azole “resistant” isolates.

**Table 2:**
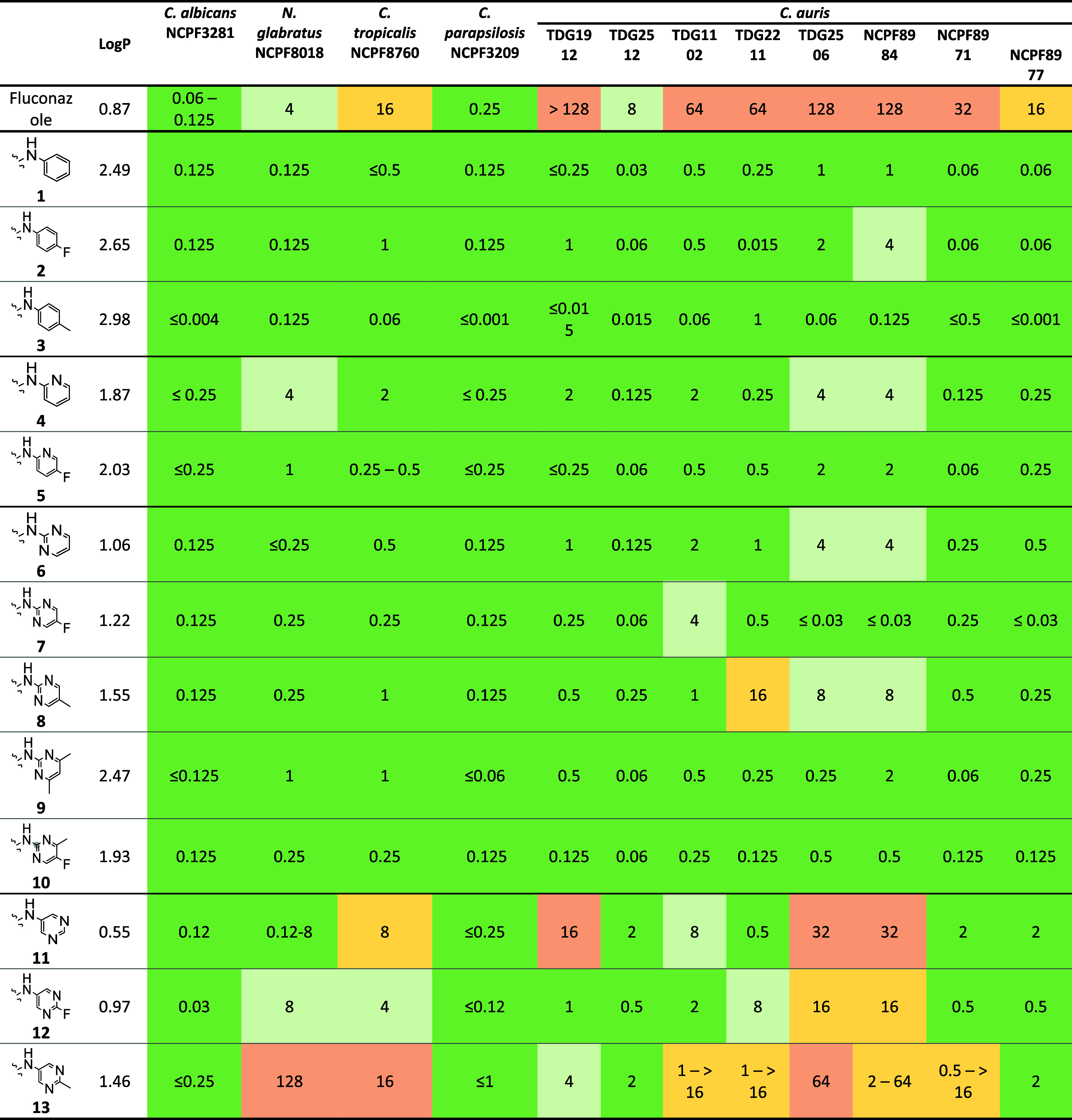
Antifungal Activity (μg/ml) of Modified Fluconazole Compounds Against and Their Corresponding LogP Value

The goals of the structural modification were to understand the role of fluorine and methyl at the 4-position of the terminal aromatic or heteroaromatic ring and the effect of introduction of heteroatoms to the phenyl ring on antifungal activity of these compounds. Compound **1** showed excellent activity across all strains, including the drug-resistant strains. The MICs against all strains were between 0.03 and 1 μg/mL, compared to fluconazole, which was inactive against the majority of the panel. Introduction of F at the 4-position of the phenyl ring maintained activity across all strains with the exception of NCPF 8984 where a 4-fold reduction in activity was observed.

On the other hand, introducing a methyl group at the 4-position of the phenyl ring resulted in significantly improved activity across all strains, including strains. In some cases, the activity increased by as much as 20-fold. Introducing a heteroatom at position 2 in the form of nitrogen maintained activity across all strains, but a noticeable reduction was observed in a few strains, particularly NCPF8018, where a 32-fold reduction was noted. A 4-fold reduction was noted for TDG2506 and NCPF8984. Interestingly, the introduction of a fluorine atom, as seen in compound **5,** restored activity to the levels observed for compound **1** across all strains tested. This suggests that fluorine at the 4-position of the pyridine ring is preferred over an unsubstituted pyridine, a preference not observed in the case of the phenyl ring.

For the next set of compounds, the phenyl ring was replaced with a pyrimidine ring. Compounds **6, 7, 8, 9**, and **10** contained a pyrimidine ring with nitrogen atoms that are *ortho-* to 2°-amine linkage. In the case of the unsubstituted pyrimidine (compound **6**), it maintained excellent activity across all strains tested, with MICs ranging from 0.12 to 4 μg/mL. However, for TDG2506 and NCPF8984, a 4-fold reduction was observed. Similar to what was observed for compound **5**, where the introduction of fluorine restored activity, compound **7**, which contains a 4-substituted fluorine, also showed a significant improvement in activity, with MIC values comparable to or in some cases better than those of compound **1**. Interestingly, only for strain TDG1102 was an 8-fold reduction in activity observed. In contrast, for other strains such as TDG2506 and NCPF8984, a 30-fold improvement in activity was noted.

When the fluorine was replaced by a methyl group, as in compound **8**, the activity was reduced, particularly across all strains. This was surprising, given that in the case of compound **3**, which contains a phenyl ring, replacing fluorine with methyl resulted in improved activity. This suggests that the electronic environment of the terminal heteroaromatic ring, in this case, pyrimidine, played a key role in the interaction of these compounds with the target enzyme, affecting their activity.

In compound **9**, a dimethyl substitution was made at positions 3 and 5 of the pyrimidine ring. Surprisingly, this compound showed a significant improvement in activity, making it one of the most active compounds, with activity comparable to that observed for compounds **1** and **5**. Introducing a methyl substitution at the **3**-position of compound **7,** as seen in compound **10**, maintained activity, suggesting that these hydrophobic substitutions are well tolerated in pyrimidine ring-containing compounds where nitrogen atoms are *ortho*- to 2°-amine linkage.

Finally, for the Library A compounds, three further compounds were synthesized, where the positions of the nitrogens in the pyrimidine ring were flipped compared to the previous series, placing the nitrogens at the *meta*- to 2°-amine linkage. The unsubstituted compound **11** showed relatively poor activity compared to compound **1** (the phenyl compound), particularly against strains, where reductions in activity of 32- to 64-fold were observed. Introducing fluorine at position 4 of this compound restored some activity, but it remained significantly lower compared to both the unsubstituted phenyl ring compound **1** and the unsubstituted pyrimidine compound **6**. Finally, when a methyl substitution was made at the 4 position of this ring, as seen in compound **12**, a further reduction in MIC was observed. The structure–activity relationship observed for the Library A compounds suggests that the effects of fluorine and methyl substitutions on activity are highly dependent on the core aromatic or heteroaromatic ring. These substitutions are well tolerated in phenyl and pyridyl rings, while their effects on pyrimidine rings are more variable.

### Synthesis of Library B

One interesting observation was the hydrophobicity, as measured by LogP, of the members of Library A. Except for compounds **11** and **12**, all other compounds had a higher LogP, indicating they are more hydrophobic compared to fluconazole (Table 2). Almost all of the compounds, except for compound **13**, showed significantly superior activity compared to fluconazole. This suggests that the hydrophobicity of the azole compounds potentially plays an important role in their interaction with the target enzyme Erg11 and in the *in vitro* activity of this compound series against azole-resistant Candida strains.

After reviewing the MIC activity data of Library A compounds, compound **7**, which contains a pyrimidine ring with nitrogens at the ortho positions relative to the amine linker and a fluorine atom at the 4-position, appeared to have the best overall activity profile. Therefore, the terminal heteroaromatic fragment present in compound **7** was selected to design the next set of compounds, where the linker for aminopiperidine was varied. This was done specifically to explore the potential of introducing different types of linkers in the design and development of modified azole compounds that are active against resistant strains—a strategy that has not been previously reported in the literature.

We selected seven different ring structures to modify compound **7**. These included various types of linkers, such as spiro and fused linkers as well as six- and eight-membered linkers to introduce different types of ring strains. For compound **15**, we selected a piperazine linker. A [1,4’-bipiperidin]-4-amine was used as the linker for compound **16**, while 2-azaspiro[4.5]­decan-8-amine was chosen for compound **17**. For compound **18**, we used 2,8-diazaspiro[4.5]­decane as the linker. A fused ring like octahydropyrrolo­[3,4-*c*]­pyrrole was selected for compound **19**, azepan-4-amine was chosen for compound **20**, and, finally, a 1,4-diazepane ring, which is a seven-membered ring, was selected for compound **21**.

The linker-heteroaromatic fragments, **15d** to **21d,** were either commercially obtained or synthesized according to Scheme 2. The final modified azole compounds **15** to **21** were synthesized following Scheme 2.

**Scheme 2:**
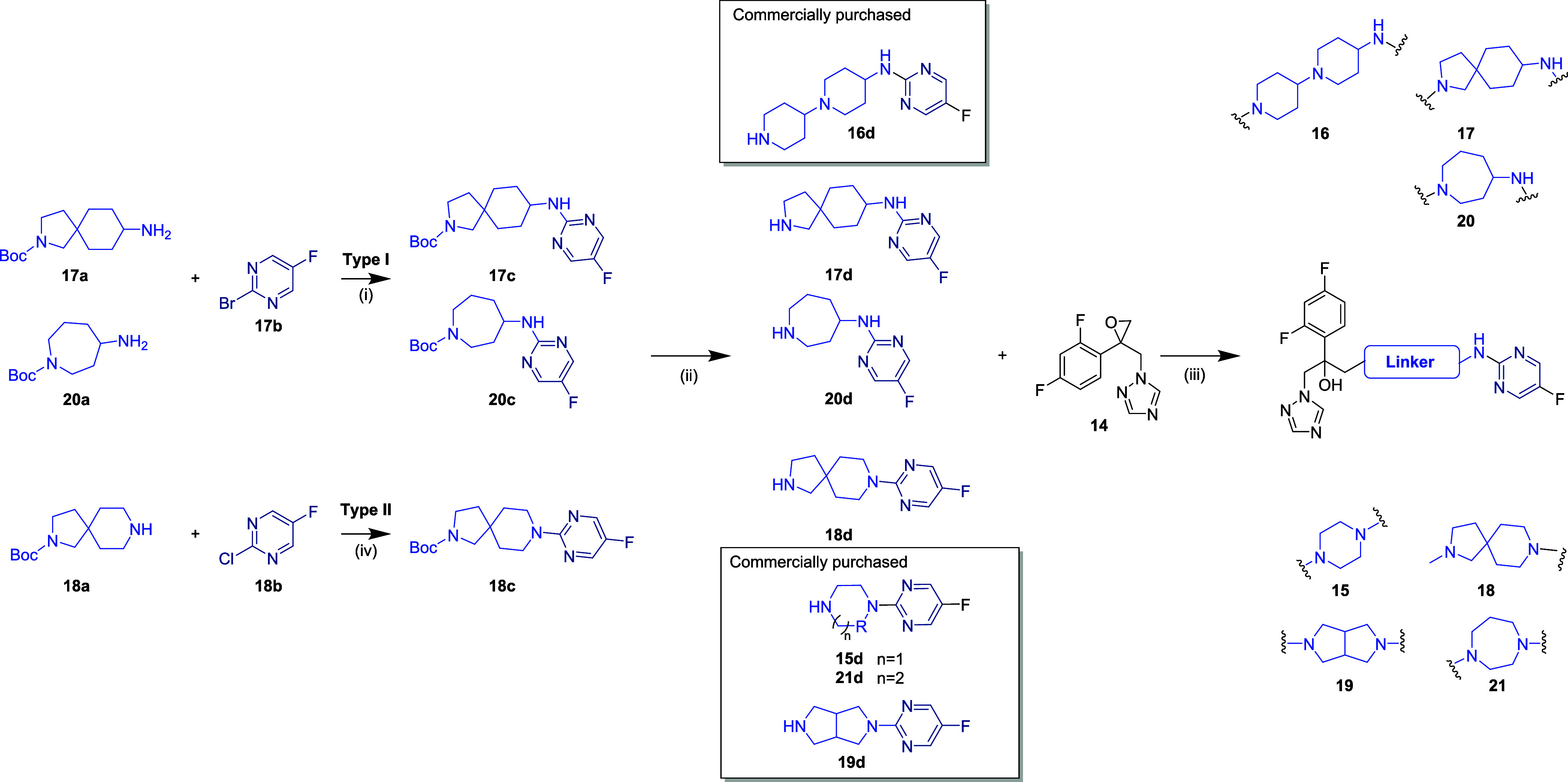
General Synthetic Routes for Library B, where the Terminal Heteroaromatic Fragment of 7 was Connected to the Fluconazole Core via Different Linkers^a^

a Conditions: (i) NaOtBu, JohnPhos, Pd_2_(dba)_3_, dioxane, 80 °C overnight; (ii) 4 M HCl in 4-dioxane, 2 h; (iii) Et_3_N, EtOH, 80 °C, overnight; (iv) K_2_CO_3_, DMSO, 70 °C, overnight or EtN­(Pr-i)_2_, DMF, 130 °C, overnight.

### Antifungal Activity of Library B

The synthesized compounds were evaluated against the same panel, which included clinically important strains and drug-resistant strains. From the activity data shown in Table 3, it can be seen that all linkers, except for the bipiperidine-4-amine linker used in compound **16** and the fused linker octahydropyrrolo­[3,4-*c*]­pyrrole used in compound **19**, were generally well-tolerated. Compounds containing other linkers either maintained their activity against these strains or, in some cases, like compound **18**, showed improved activity compared to compound **7** against most strains tested.

**Table 3:**
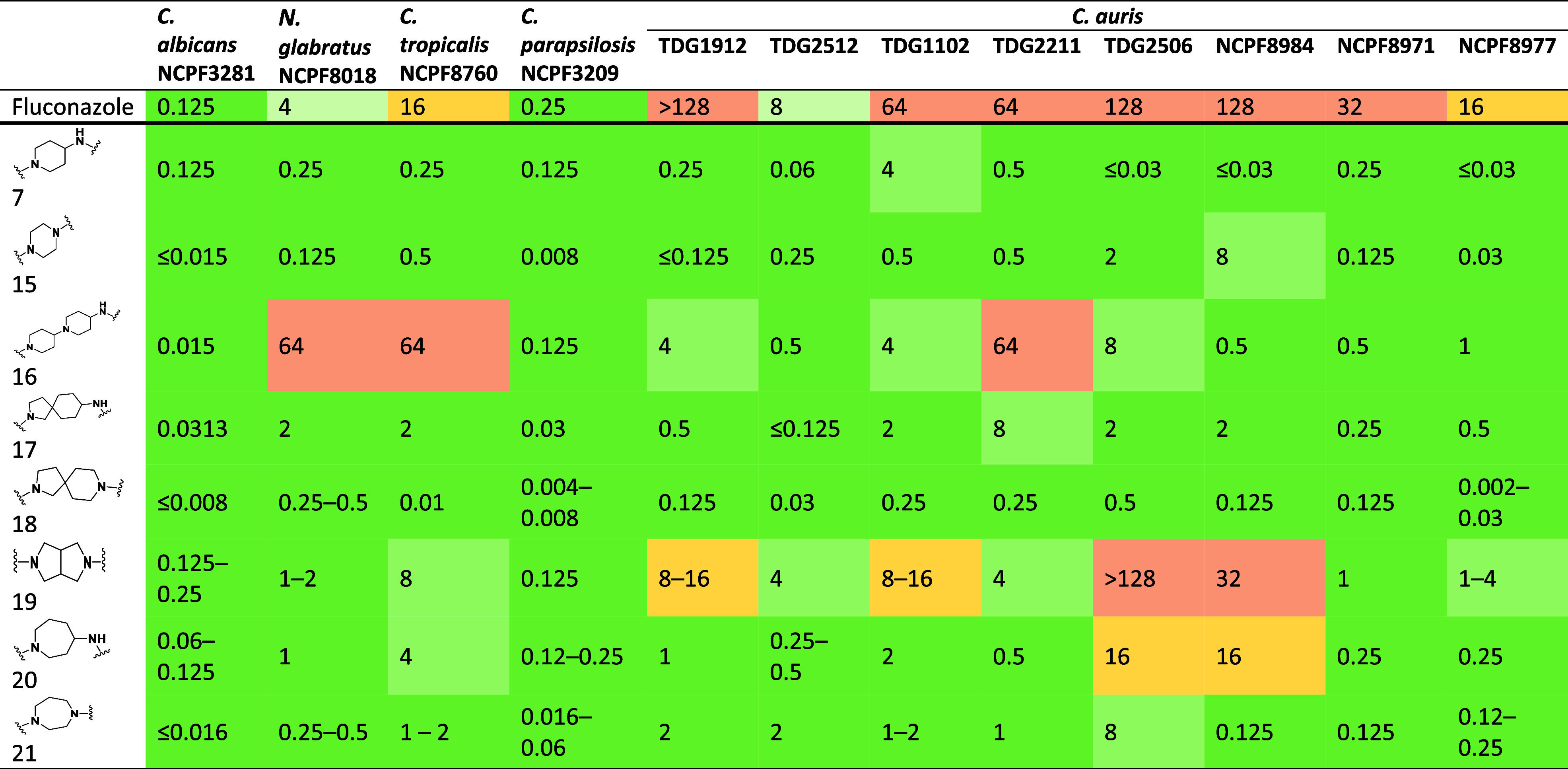
Antifungal Activity (μg/ml) of Modified Fluconazole Compounds Against from Library B Where Different Linker Types Were Utilized

Compound **18**, which contains a 2,8-diazaspiro[4.5]­decane linker, demonstrated a 4- to 8-fold improvement in activity across various strains and had the best overall activity profile among all the compounds synthesized. The compound was highly active against NCPF3281 (MIC ≤ 0.008 μg/mL), NCPF3209 (MIC 0.004 μg/mL), and NCPF8977 (MIC 0.002 μg/mL). The MIC of this compound was less than 0.5 μg/mL against all other strains. Compound **15**, which contains a piperidine linker, also showed good activity across the board, except against NCPF8984, where an approximately 256-fold reduction in activity was observed and TDG2506 with a 64-fold reduction in activity. The compound had an MIC of 0.5 μg/mL or less against all other strains. Similarly, compound **17** maintained activity for most strains, but generally exhibited a 2- to 8-fold reduction in activity (Table 3).

Compound **20**, on the other hand, showed some variability in its activity. Its activity against NCPF8760 was 16-fold lower compared to compound **7**. Interestingly, against two strains, TDG2211 and NCPF8984, compound **20** also showed significantly reduced activity, with more than 512-fold reduction observed. However, it showed comparable activity against all other strains.

Compound **21**, which had a 7-membered diazepane ring, generally maintained good activity across all strains tested. However, the activity was lower against strains, with a 4- to 64-fold reduction observed for most strains, except for TDG2506, which showed a more than 256-fold reduction in activity. It showed comparable activity against other clinically important strains, with MICs ranging from 0.015 to 4 μg/mL (Table 3).

From the activity profile of these second-generation compounds, it appears that linker length did not play a specific role on activity. Compounds **16**, **17**, **18**, and **19**, which had either fused or spiro linkers, generally had longer linkers compared to compounds **7**, **15**, **20**, and **21**, but these lengths did not correlate with activity. For example, compound **18**, with a longer spiro linker, and compound **7**, with a relatively short 4-aminopiperidine linker, showed the overall best activity profile and can be considered as lead compounds. These two compounds showed a more than 128- to 512-fold increase in activity compared to fluconazole against strains. This suggests that the nature of the linker is more important than its length. Overall, when the antifungal activity of compounds from both Library A and Library B was compared, compounds **7**, **18**, and **21** emerged as the most promising, exhibiting significantly superior *in vitro* activity compared to fluconazole against both and a range of strains. Compounds **7** and **18** also demonstrated superior MIC values against these strains compared to voriconazole (Table S1), which possesses a differentcore scaffold than fluconazole. Based on these findings, compound **7** was selected for further mechanistic and efficacy studies to evaluate the therapeutic potential of this modified azole series.

### Investigation of Stereoselective Synthesis of Compound Libraries Using Chiral HPLC

During the synthesis of both library A and library B compounds, it was considered that the nucleophilic ring opening of epoxide intermediate **14**by bulky amine fragments—containing heteroaliphatic linkers and aromatic or heteroaromatic groups—could potentially lead to the formation of racemic mixtures. However, the initial analytical data suggested the preferential formation of a single enantiomer, likely due to the steric constraints imposed by the bulky amine substituents, which could direct the nucleophilic attack in a stereoselective manner. To investigate this hypothesis further, chiral high-performance liquid chromatography (HPLC) analysis was conducted using compound **7**, one of the most potent molecules identified across both libraries. Chiral HPLC studies, performed under multiple solvent ratios, consistently revealed a single peak with no evidence of diastereomeric or enantiomeric impurities (Figure S19a–c). These findings strongly suggest that the synthetic strategy employed favored the stereoselective formation of a single enantiomer, likely driven by the steric and electronic properties of the amine nucleophiles. This outcome aligns with prior reports in the literature where epoxide ring openings with highly hindered amines have shown significant stereochemical preference.^44^

### Mechanism of Action Study of Modified Azole Compounds

#### In silico Study with Lanosterol 14α-Demethylase (LDM) Enzyme in 

To further explore the differences in activity observed between fluconazole and modified compound **7**, particularly in strains, we evaluated the binding of these two compounds to the lanosterol 14α-demethylase (LDM) enzyme in using molecular docking. As shown in Figure 2, interesting differences were observed in the 2D and 3D bindings of both compounds. Fluconazole and compound **7** were found to bind to adjacent binding pockets. However, while fluconazole interacted with a more hydrophilic overall binding pocket, compound **7** interacted with a predominantly hydrophobic pocket and engaged more amino acid residues within the binding site. The length of the molecule may have contributed to these interactions, but the nature of the binding and the differences observed between compound **7** and fluconazole likely played a role in their ability to inhibit the target enzyme and their effectiveness in killing strains. The Chemscore and binding affinity values (Table 1) suggest that compound **7** binds more tightly to the LDM enzyme, and this tight binding, along with the differences in interactions, particularly in the hydrophobicity of key binding residues, may explain compound **7**’s superior ability to kill strains.

**Figure 2:**
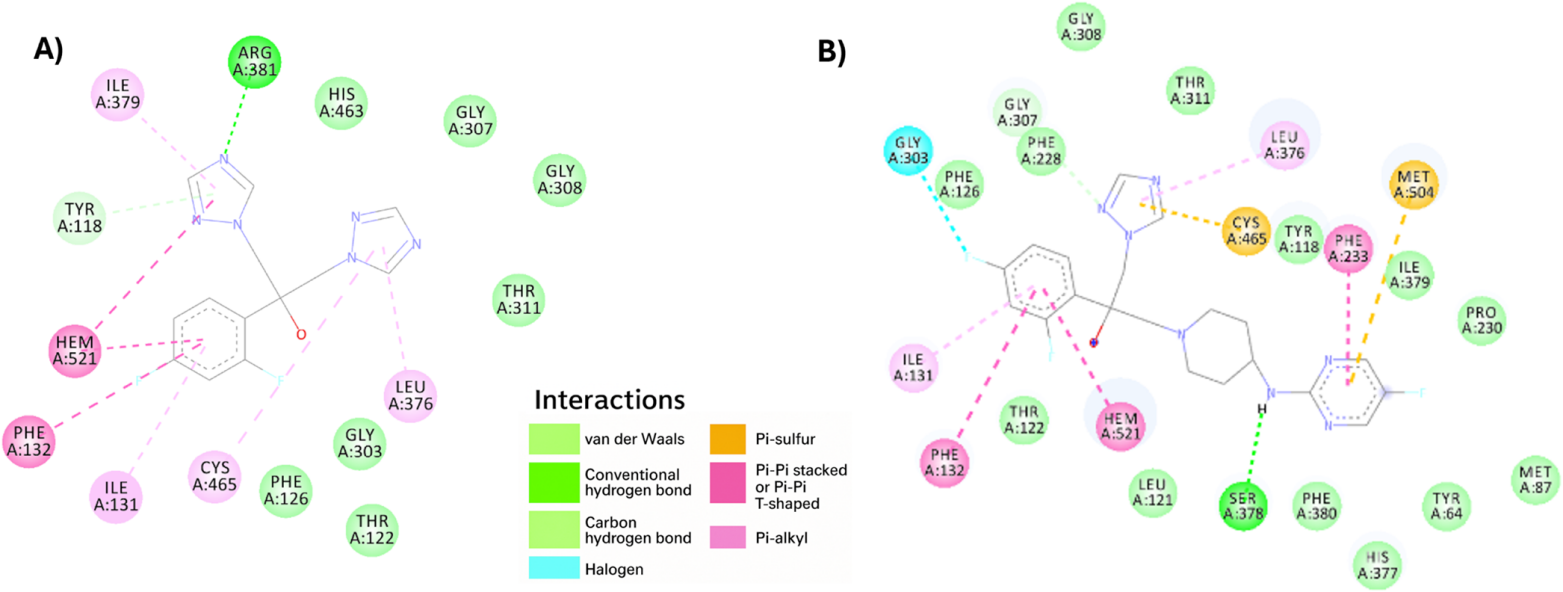
Differences in 2D binding interactions observed for fluconazole (A) and compound **7** (B) with a lanosterol 14α-demethylase (LDM) enzyme in (PDB ID 5TZ1 was used as a template for homology modeling).

####  CYP51 Inhibition Assay

To further explore the antifungal potential of compound **7**, we assessed its ability to inhibit CYP51, a key enzyme involved in ergosterol biosynthesis and a well-established antifungal drug target. Enzymatic inhibition was compared to that of fluconazole. Compound **7** demonstrated potent inhibition of CYP51 activity, with an IC_50_ value of approximately 0.40 μM. In comparison, fluconazole exhibited an IC_50_ of ∼0.6 μM, consistent with values reported in previous studies.^45^ The observed potency of compound **7** highlights its potential as a promising CYP51-targeting agent. These data provide support for the continued evaluation of compound **7** as a selective antifungal candidate for ., including potential applications against drug-resistant clinical isolates.

### Accumulation Assay of Compound 7 in 

To further investigate the superior *in vitro* activity of compound **7** against a broad panel of strains, including those harboring multiple target mutations, a modified intracellular accumulation assay was performed.^46^ We hypothesized that structural modifications in azole compounds, such as those present in compound **7**, enhance their ability to accumulate within fungal cells, thereby improving antifungal efficacy, even in strains exhibiting resistance-conferring mutations. The results demonstrated that compound **7** accumulated to significantly higher levels than fluconazole in strain TDG1912 under identical incubation conditions (*p* = 0.001), suggesting enhanced intracellular retention (Figure 3). Given that compound **7** was derived through rational modification of the fluconazole core scaffold, these findings support the design rationale for the development of next-generation modified azole derivatives with improved pharmacodynamic properties. This likely contributed to the greater antifungal activity of this compound series against resistant isolates.

**Figure 3:**
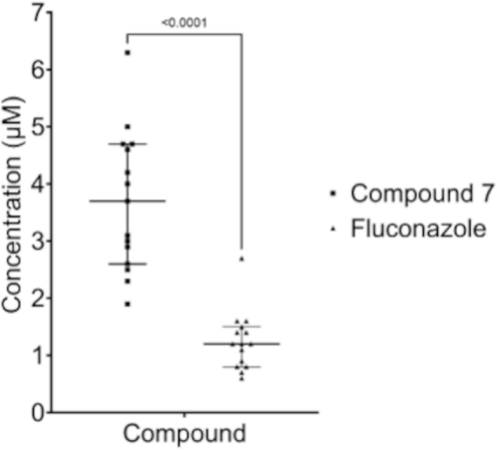
Intracellular accumulation of azole antifungals in strain TDG1912. Compound **7** showed significantly greater accumulation than fluconazole (*p* = < 0.0001).

### Biofilm Eradication Activity of Compound 7 against TDG1912

A biofilm eradication assay was conducted to evaluate the ability of compound **7** to reduce the number of established TDG1912 biofilms. Biomass quantification was performed and expressed as a percentage of untreated controls. Amphotericin B served as a positive control and demonstrated potent biofilm clearance across a range of concentrations, consistent with its known fungicidal activity. Fluconazole exhibited minimal biofilm reduction, confirming its limited efficacy against mature biofilms. Compound **7** significantly reduced biofilm biomass, exhibiting eradication activity comparable to, and in some concentrations slightly greater than, voriconazole (Figure 4). This suggests that compound **7** is effective in disrupting preformed biofilms, and this class of modified azoles may have therapeutic potential in the treatment of biofilm-associated infections caused by this emerging multidrug-resistant pathogen.

**Figure 4:**
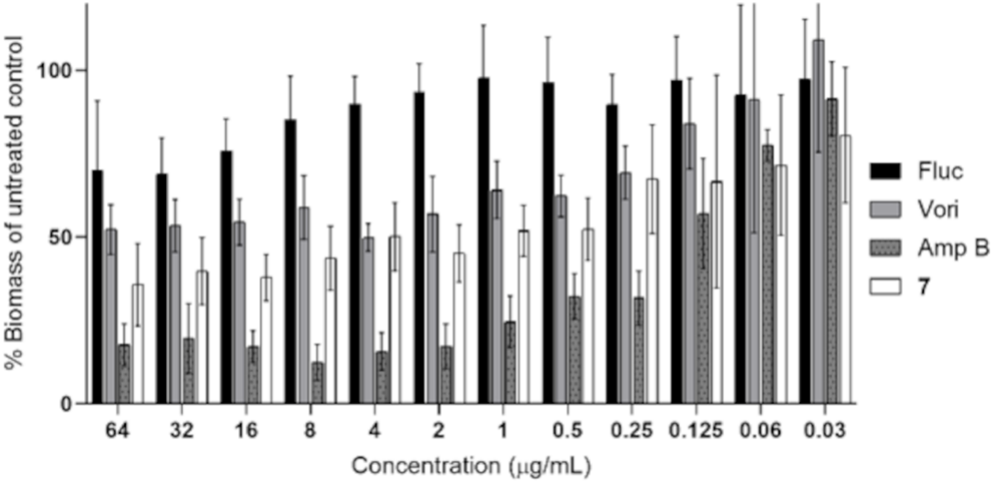
Eradication of preformed TDG1912 biofilms by antifungal agents. Established biofilms were treated with serial dilutions of fluconazole (Fluc), voriconazole (Vori), amphotericin B (Amp B), or Compound **7** for 24 h. Residual biofilm biomass was quantified and normalized to the untreated control (% biomass). Data represent the mean ± SD of three independent experiments.

### Time-Kill Assay of Compound 7

Time-kill analysis evaluates the kinetics and efficacy of antifungal compounds over time. It monitors fungal growth at specific intervals after treatment with antifungals at concentrations relative to the MIC. This method reveals differences in the behavior of compounds with identical MICs, providing insights into their mode of action. A reduction of colonies by ≤3-log indicates fungistatic activity, while >3-log reduction suggests fungicidal action. Azoles are typically fungistatic, but the higher *in vitro* activity observed in modified azoles warranted an evaluation of their mode of action using a time-kill kinetics assay. In this study, Compound **7**, fluconazole, and voriconazole were tested against TDG1912 over 24 h at 4× MIC_50_ (Figure 5).

**Figure 5:**
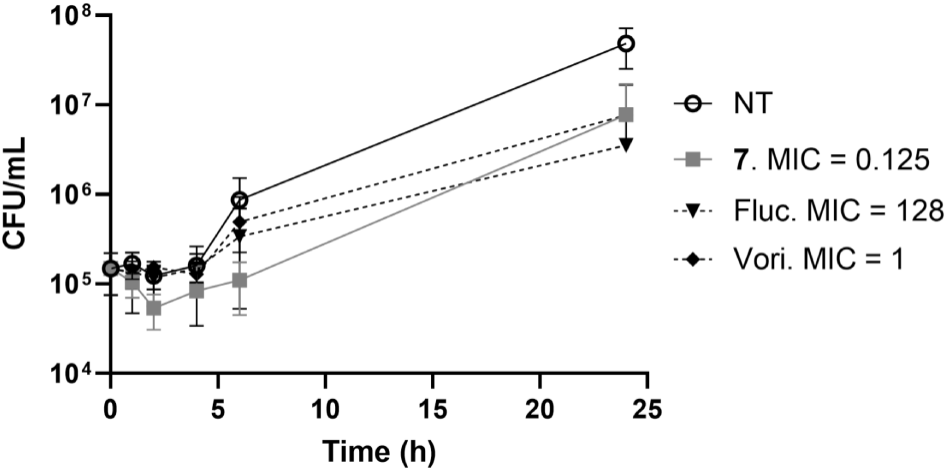
Time-kill analysis of compound **7**, alongside two commercial reference antifungal drugs, fluconazole (Fluc) and voriconazole (Vori) against TDG1912. The limit of detection (LoD) is 1000 CFU/mL. MIC_50_ results are given in μg/mL. NT represents no treatment.

Compared with the untreated control sample, time-kill curves for all drug-treated samples exhibited a slightly smaller fungal population size. Notably, compound **7** stood out among the three tested compounds, demonstrating inhibition of fungal growth for at least 6 hours of incubation. However, regrowth of TDG1912 was observed after the 6 h time point. It is important to note that the MIC value used for the time-kill assay corresponds to drug concentrations inhibiting 50% of fungal growth, as defined by EUCAST guidelines for azoles. All compounds, whether modified or commercially available, exhibited a fungistatic nature, inhibiting fungal growth at varying efficacies but not killing the fungi (Figure 5).

### Efficacy Study in Infection Model

After showing excellent *in vitro* activity against drug-resistant strains, compound **7** was selected to test the *in vivo* efficacy of these modified azoles in a model^47^ infected with (TDG1912). In the infection model, compound **7** demonstrated significant antifungal efficacy at 50 mg/kg and 20 mg/kg doses (Figure 6 and Table S3, *p* < 0.0001), offering significantly superior protection comparable to the standard azole fluconazole. At 10 mg/kg, however, the protective effect of compound **7** was diminished, as shown by a reduction in survival and supported by statistically significant *p*-values. The positive control amphotericin B exhibited potent fungicidal activity at 20 mg/kg and 10 mg/kg, with both doses resulting in highly significant survival benefits (*p* < 0.0001 across tests) (Figure S20). However, treatment at 50 mg/kg was associated with apparent toxicity, as reflected by the drop in survival (Table S3), suggesting a narrow therapeutic window. Fluconazole showed only modest protection at the highest tested dose of 50 mg/kg, with no significant efficacy observed at 20 or 10 mg/kg. These results reinforce the superior performance of compound **7** at equivalent or lower doses compared to fluconazole.

**Figure 6:**
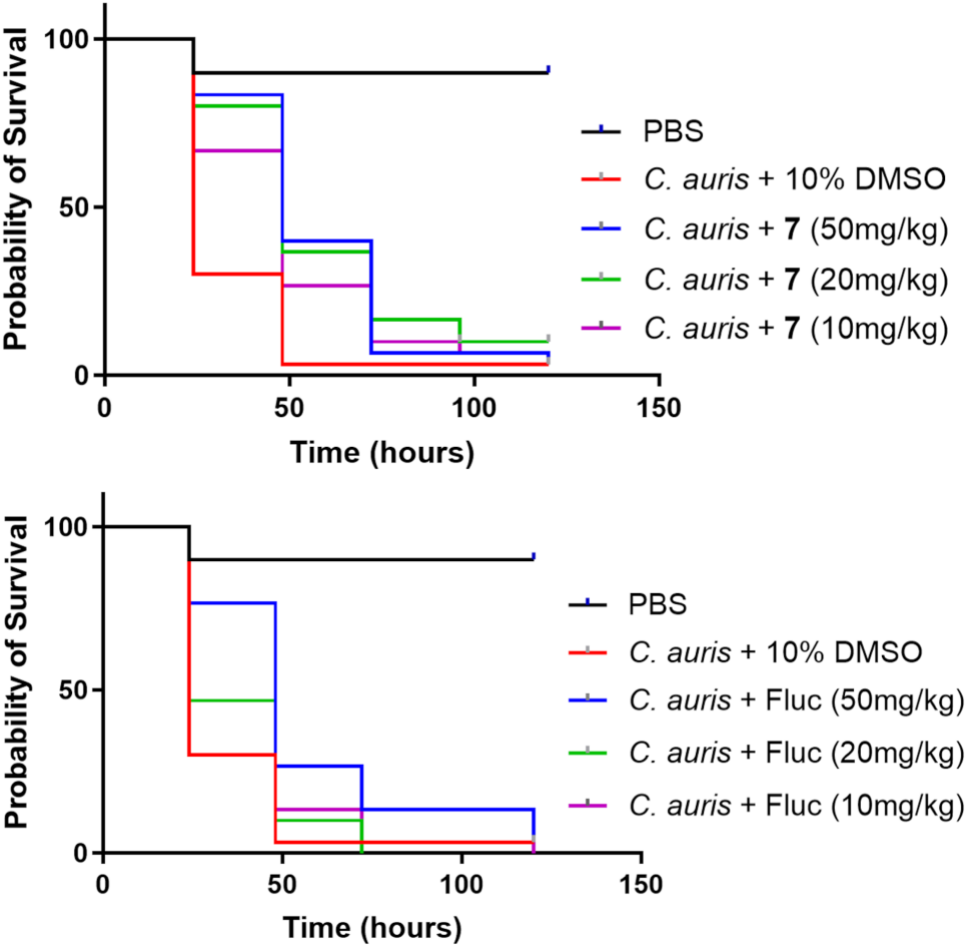
Efficacy of modified fluconazole compound **7** and fluconazole (Fluc) against TDG1912 infection in the *G. mellonella* model.

### Efficacy Study in Infection Model

Compound **7** was further evaluated for its *in vivo* efficacy using a infection model^48^ challenged with (strain TDG1912). Following an approach similar to that of the model, the initial assessment focused on compound toxicity. Compound **7** exhibited no observable toxicity at a dose of 50 mg/kg. Upon infection with 2 × 10^3^ yeast cells per fly, treatment with compound **7** conferred notable protection to adult flies, whereas fluconazole failed to demonstrate any protective effect (Figure 7). Furthermore, treatment with compound **7** at both 50 and 20 mg/kg significantly improved survival compared to the untreated group, with *p*-values of 0.0024 and 0.0377, respectively, as determined by the Mantel–Cox log-rank test (Figure 7).

**Figure 7:**
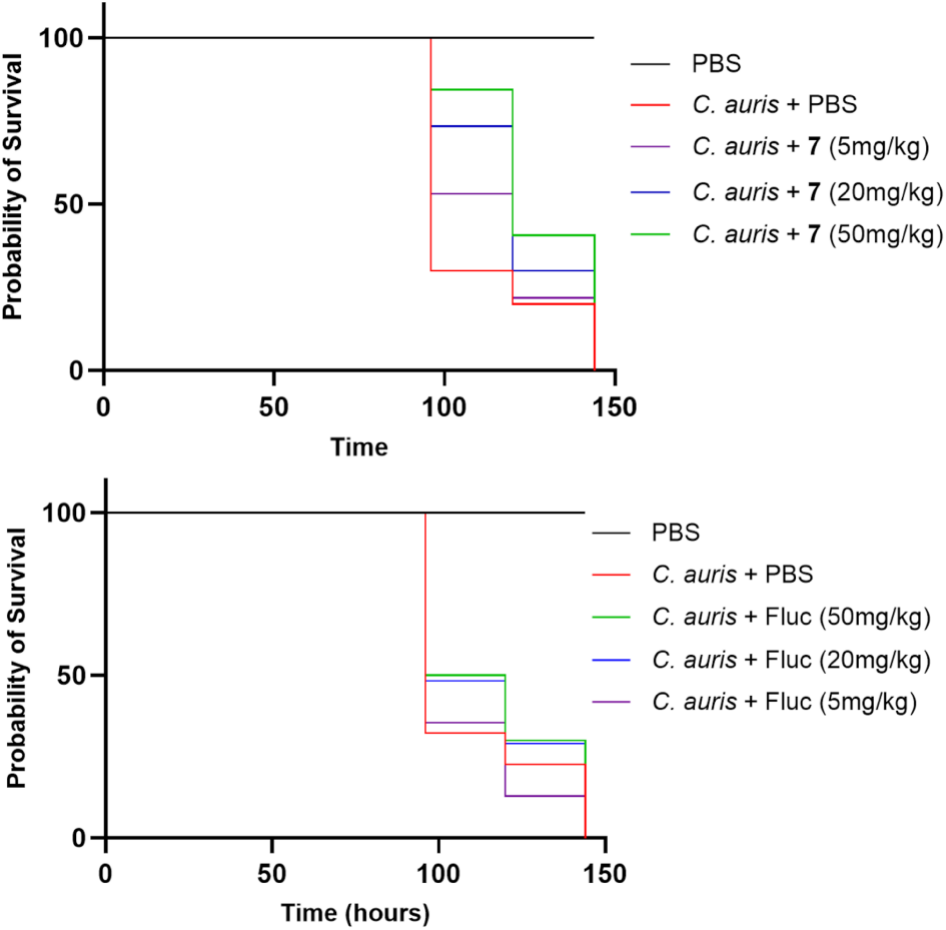
Efficacy of compound **7** and fluconazole (Fluc) against TDG1912 infection in the model at an infection dose of 2 × 10^3^ yeast cells per fly.

At an infection dose of 1 × 10^4^ yeast cells per fly, significant lethality was observed in the untreated group as early as 24 h postinfection, in contrast to infections with lower fungal loads, where lethality typically occurred around 96 h. Notably, even at this high infection level, treatment with compound **7** at 50 mg/kg conferred statistically significant protection compared to the untreated group (*p* = 0.0013, Mantel–Cox log-rank test), and this effect was markedly stronger than that of fluconazole at the same dose (*p* = 0.0269, Mantel–Cox log-rank test) (Figure S22).

## Conclusions

In summary, this study focuses on the design, synthesis, and biological evaluation of a novel series of modified azole compounds. Structural modifications, including variations in linker types and substitution patterns on terminal aromatic or heteroaromatic groups, revealed key features that enhance antifungal activity against drug-resistant . Compound **7** emerged as a lead candidate, showing broad-spectrum antifungal activity and retention of potency across a diverse panel of strains. Mechanistic studies confirmed that compound **7** inhibits CYP51, the established azole target, and demonstrates superior biofilm eradication compared to fluconazole. An intracellular accumulation assay showed significantly improved uptake of compound **7** over fluconazole, supporting the design rationale. *In vivo* efficacy studies in both and models confirmed its ability to protect against infection. These findings validate the potential of this new azole scaffold for further development as a treatment for invasive candidiasis, particularly infections caused by multidrug-resistant .

## Experimental Section

### Chemistry

#### General Chemistry

^1^H NMR and ^13^C NMR spectra were recorded on a Bruker Spectrospin Spectrometer (400 MHz, 25 °C) equipped with a SampleXpress autosampler system. Chemical shifts δH and δC are recorded in parts per million (ppm) and referenced to the residual solvent peak. Coupling constants (J) are recorded to the nearest 0.01 Hz. Assignment of ^1^H and ^13^C NMR spectra was made using the aid of TopSpin 3.5 software from Bruker, ACD/Laboratories, or MestReNova from Mestrelab Research. All compounds tested for biological activity were confirmed to be >95% pure using two independent HPLC analysis methods.

The LC–MS system used is a Waters Alliance 2695 system with an elution in gradient. Low-resolution mass spectra were analyzed and recorded on a Waters QZ instrument using electrospray ionization (ESI) and coupled to a high-performance liquid chromatography (HPLC) system. Selected mass-to-charge ratio peaks (*m*/*z*) are quoted in Daltons. HPLC-grade solvents were used for the mobile phase, and a Phenomenex Monolithic C18 50 mm × 4.60 mm column was used for the stationary phase. The detect method used UV detection performed on a Waters 2996 photo array detector.

Thin-layer chromatography (TLC) was performed using Merck aluminum foil-backed sheets precoated with 0.2 mm Kieselgel 60 F254. Product spots were visualized by UV irradiation (λmax = 254 or 365 nm). Manual flash column chromatography was carried out using Aldrich silica gel 60 Å, 40–63 μm (230–400 mesh). Eluting solvents and retention factors (Rf) were indicated in the text.

The initial optical rotation of compounds was measured with a single wavelength polarimeter ADP440 by Bellingham & Stanley at λ = 589 nm. Further optical rotatory dispersion (ORD) measurements of selected compounds were measured with Chirascan spectrometers by Applied Photophysics and were analyzed with Pro-Data Chirascan.

All reactions were carried out in oven-dried glassware, and all reagents were obtained commercially from Aldrich Chemicals Ltd., Alfa Aesar Ltd., Fluorochem, Fisher Scientific, or Apollo Chemicals Ltd.

### Chemical Synthesis

#### General Procedure for Intermediate Synthesis: (i) Using Reducing Borohydride

The aniline (1.0 equiv) synthesized in the previous step or purchased commercially was dissolved in anhydrous DCM (0.1 mmol/mL) in a round-bottomed flask upon stirring, followed by the addition of the Boc-protected linker fragment (1.5 equiv), Na­(OAc)_3_BH (2.0 equiv), and AcOH (2.0 equiv) successively. After overnight stirring at ambient temperature, the mixture was consecutively quenched with NaOH (aq), extracted with DCM, dried with anhydrous MgSO_4_, and evaporated *in vacuo*. The crude mixture was purified with flash column chromatography gradiently using chloroform and methanol to get the target pure product.

##### *tert*-Butyl 4-(Phenylamino)­piperidine-1-carboxylate **1a**

White solid (56%); ^1^H NMR (400 MHz, 25 degree, CDCl_3_) σ: 1.46 (9H, s), 2.04 (2H, dd, J = 13 Hz, 2 Hz), 2.44 (1H, t, J = 6 Hz), 2.88 (2H, t, J = 12 Hz), 3.42 (1H, tt, J = 10 Hz, 4 Hz), 3.72 (1H, t, J = 6 Hz), 4.05 (2H, d, J = 8 Hz), 5.30 (1H, s), 6.68–6.81 (3H, m), 7.20 (2H, dd, J = 8 Hz, 7 Hz); ^13^C NMR (101 MHz, 25 degree, CDCl_3_) σ: 58.5, 32.0, 41.3, 79.8, 80.6, 115.3, 129.4, 129.6, 154.9, 208.0; *m*/*z*: (ESI^+^) 277.1 ([M + H]^+^); *R*_*f*_: 0.9 (1:1 Hexane: Ethyl acetate); Purity: 96%.

##### *tert*-Butyl 4-(P-Tolylamino)­piperidine-1-carboxylate **3a**

Off-white solid (51%); ^1^H NMR (400 MHz, 25°, CDCl_3_) σ: 1.29–1.39 (2H, m), 1.46 (9H, s), 2.03 (2H, dd, J = 12 Hz, 2 Hz), 2.24 (3H, s), 2.89 (2H, t, J = 12 Hz), 3.38 (1H, tt, J = 10 Hz, 4 Hz), 4.04 (2H, d, J = 9 Hz), 4.04 (1H, s, br), 6.60 (2H, d, J = 8 Hz), 7.00 (2H, d, J = 8 Hz); ^13^C NMR (101 MHz, 25 degree, CDCl_3_) σ: 20.5, 28.6, 32.3, 41.3, 51.4, 79.7, 114.6, 127.9, 130.0, 143.5, 154.9; *m*/*z*: (ESI^+^) 291.2 ([M + H]^+^); Rf: 0.03 (Ethyl acetate); Purity: ≥97%.

##### *tert*-Butyl 4-((2-Fluoropyrimidin-5-Yl)­amino)­piperidine-1-carboxylate **12a**

Off-white solid (49%); ^1^H NMR (400 MHz, 25 degree, CDCl_3_) σ: 1.41 (9H, s), 1.77–1.83 (1H, m), 1.98 (2H, dd, J = 13 Hz, 3 Hz), 2.88 (2H, t, J = 12 Hz), 2.97 (1H, ddd, J = 13 Hz, 10 Hz, 3 Hz), 3.35 (1H, tt, J = 10 Hz, 4 Hz), 3.60 (1H, s, br), 4.03 (2H, d, J = 13 Hz), 7.97 (2H, s); ^1^³C NMR (101 MHz, 25 degree, CDCl_3_) σ: 28.4, 31.7, 34.2, 50.9, 67.6, 79.6, 80.0, 139.0 (d, J = 5 Hz), 144.7 (d, J = 11 Hz), 154.7, 156.3 (d, J = 211 Hz); *Rf*: 0.2 (1:9 Ethyl acetate: DCM); Purity: ≥95%.

##### *tert*-Butyl 4-((2-Methylpyrimidin-5-Yl)­amino)­piperidine-1-carboxylate **13a**

White solid (53%); ^1^H NMR (400 MHz, 25 degree, CDCl_3_) σ: 1.36–1.43 (2H, m), 1.45 (9H, s), 2.01 (2H, dd, J = 13 Hz, 3 Hz), 2.65 (3H, s), 2.95 (2H, t, J = 12 Hz), 3.43 (1H, tt, J = 10 Hz, 4 Hz), 4.03–4.07 (2H, m), 4.42 (1H, s, br), 8.20 (2H, s); ^1^³C NMR (101 MHz, 25 degree, CDCl_3_) σ: 23.7, 28.6, 29.8, 31.9, 50.1, 80.0, 138.9, 141.1, 154.8, 154.8; *m*/*z*: (ESI^+^) 293.1 ([M + H]^+^); Rf: 0.5 (9:1 Ethyl acetate: MeOH); Purity: ≥98%.

#### General Procedure for Intermediate Synthesis: (ii) Using Base

The starting material of pyrimidine (1.0 equiv) in a mixture of anhydrous MeCN (1.315 mmol/mL) and dimethylacetaminde (5.26 mmol/mL) was stirred under inert gas, while the Boc-protected linker (1.2 equiv) and DIPEA (1.2 equiv) were added slowly. The reaction was carried out in a microwave at 160 °C for 1 h and at 180 °C for another 30 min. The crude mixture was obtained by direct solvent evaporation. The mixture was purified by gradient flash column chromatography using ethyl acetate and hexane with 1% TEA.

##### 5-Methyl-N-(piperidin-4-Yl)­pyrimidin-2-amine **8a**

Pale-yellow solid (42%); ^1^H NMR (400 MHz, 25 degree, MeOH-d4) σ: 1.34 (2H, ddd, J = 24 Hz, 12 Hz, 3 Hz), 1.92 (2H, d, J = 12 Hz), 2.10 (3H, s), 2.92 (2H, t, J = 12 Hz), 3.02 (1H, tt, J = 11 Hz, 4 Hz), 4.65 (2H, d, J = 13 Hz), 8.16 (2H, s); ^1^³C NMR (101 MHz, 25 degree, MeOH-d4) σ: 14.4, 34.2, 44.0, 50.1, 119.7, 159.0, 151.6; *m*/*z*: (ESI^+^) 194.1 ([M + H]^+^); *Rf*: 0.1 (10:1:0.1 Ethyl acetate: MeOH: TEA); Purity: ≥96%.

##### *tert*-Butyl 4-((4,6-Dimethylpyrimidin-2-Yl)­amino)­piperidine-1-carboxylate **9a**

Pale-yellow solid (45%); ^1^H NMR (400 MHz, 25 degree, CDCl_3_) σ: 1.20–1.30 (2H, m), 1.35 (9H, s), 1.91 (2H, d, J = 12 Hz), 2.15 (6H, s), 2.85 (2H, t, J = 12 Hz), 4.00–4.05 (1H, m), 4.59 (1H, d, J = 13 Hz), 6.18 (1H, s); ^1^³C NMR (101 MHz, 25 degree, CDCl_3_) σ: 23.7, 28.3, 32.2, 42.5, 47.5, 79.3, 109.6, 154.6, 161.4, 167.3; *m*/*z*: (ESI^+^) 307.2 ([M + H]^+^); *Rf*: 0.3 (2:8 Ethyl acetate: Hexane); Purity: ≥95%.

#### General Procedure for Intermediate Synthesis: (Iii) Using Metal Catalysis

In an oven-dried round-bottomed flask containing anhydrous toluene (0.6 mmol/mL), the pyrimidine fragment (1.0 equiv), BOC-protected linker fragment (1.0 equiv), rac-BINAP (0.04 equiv), Pd_2_(dba)_3_ (0.02 equiv), and KOtBu (1.2 equiv) were added sequentially under inert gas. The mixture was then heated under microwave irradiation at 100 °C for 2 h. After being cooled to room temperature, the mixture was then filtered through Celite. The filtrate was diluted with saturated brine and extracted with ethyl acetate three times before drying over anhydrous MgSO4 and evaporated in vacuo. The crude product was then purified by gradient flash column chromatography using hexane and ethyl acetate.

##### *tert*-Butyl 4-((5-Fluoro-4-methylpyrimidin-2-Yl)­amino)­piperidine-1-carboxylate **10a**

Off-white solid (52%); ^1^H NMR (400 MHz, 25 degree, CDCl_3_) σ: 1.26–1.40 (2H, m), 1.44 (9H, s), 1.99 (2H, d, J = 12 Hz), 2.31 (3H, d, J = 2 Hz), 2.92 (2H, t, J = 12 Hz), 3.83–4.01 (3H, m), 4.96 (1H, d, J = 7 Hz), 7.98 (1H, d, J = 1 Hz); ^13^C NMR (101 MHz, 25 degree, CDCl_3_) σ: 17.8, 28.5, 32.2, 48.7, 79.7, 144.0 (d, J = 23 Hz), 151.1 (d, J = 246 Hz), 154.9, 155.8 (d, J = 16 Hz), 158.1 (d, J = 3 Hz); *m*/*z*: (ESI^+^) 255.1 ([M – tBu + H]^+^); *Rf*: 0.8 (Ethyl acetate); Purity: ≥95%.

#### Synthesis of Fluconazole Core

##### 1-((2-(2,4-Difluorophenyl)­oxiran-2-Yl)­methyl)-1h-1,2,4-triazole (**14**)

Trimethylsulfoxonium iodide (2 equiv) was added to toluene (0.12 mmol/mL) containing 1-(2,4-difluorophenyl)-2-(1H–1,2,4-triazol-1-yl)­ethan-1-one (1 equiv) and sodium hydroxide 30% (w/w) aqueous solution (10 equiv). The mixture was heated under microwave (MW) radiation for 50 min at 80 °C. Then, the mixture was diluted with water and extracted with ethyl acetate. The organic layer was combined and washed with saturated brine, dried over anhydrous magnesium sulfate, and concentrated in vacuo. Pale yellow solid (52%); ^1^H NMR (400 MHz, 25 degree, CDCl_3_) σ: 2.83 (1H, d, J = 5 Hz), 2.90 (1H, d, J = 5 Hz), 4.47 (1H, d, J = 15 Hz), 4.79 (1H, d, J = 15 Hz), 6.73–6.82 (2H, m), 7.11–7.16 (1H, m), 7.81 (1H, s), 8.03 (1H, s); ^13^C NMR (101 MHz, 25 degree, CDCl_3_) σ: 52.1, 53.4 (d, J = 3 Hz), 56.1 (d, J = 1 Hz), 104.0 (dd, J = 26 Hz, J = 25 Hz), 111.6 (dd, J = 21 Hz, J = 3 Hz), 119.4 (dd, J = 15 Hz, 4 Hz), 129.5 (dd, J = 15 Hz, J = 10 Hz, 5 Hz), 144.0, 151.7, 160.5 (dd, J = 249 Hz, 12 Hz), 163.0 (dd, J = 251 Hz, 12 Hz); *m*/*z*: (ESI^+^) 238.1 ([M + H]^+^); *R*_*f*_: 0.24 (1:1 Ethyl acetate: Hexane); Purity: ≥95%.

##### *tert*-Butyl 8-(5-Fluoropyrimidin-2-Yl)-2,8-diazaspiro[4.5]­decane-2-carboxylate **18c**

The Boc-protected linker fragment (1.0 equiv) was dissolved in anhydrous DMF under inert gas before the addition of the pyrimidine fragment (1.275 equiv) and DIPEA (3.125 equiv). The mixture was heated at 130 °C overnight and cooled to room temperature upon reaction completion. After diluted with water, the mixture was extracted by ethyl acetate for three times and dried over anhydrous MgSO_4_ before evaporated *in vacuo*. The crude product was purified with gradient *flash* column chromatography in a mixture of DCM and ethyl acetate. White solid (39%); ^1^H NMR (400 MHz, 25 degree, CDCl_3_) σ: 1.45 (9H, s), 1.58 (4H, dd, J = 11 Hz, 6 Hz), 1.75 (2H, t, J = 7 Hz), 3.19 (1H, s), 3.27 (1H, s), 3.38–3.43 (2H, m), 3.59–3.69 (2H, m), 3.80–3.92 (2H, m), 8.16 (2H, s); ^13^C NMR (101 MHz, 25 degree, CDCl_3_) σ: 28.7, 34.5, 36.5, 40.3, 42.1, 44.3 (d, J = 26 Hz), 55.6 (d, J = 75 Hz), 79.3, 145.2 (d, J = 22 Hz), 151.5 (d, J = 248 Hz), 154.9, 158.9; *m*/*z*: (ESI^+^) 281.1 ([M – *t*Bu + H]^+^); *R*_*f*_: 0.8 (1:1 DCM: Ethyl acetate); Purity: ≥95%.

##### *tert*-Butyl 4-((5-Fluoropyrimidin-2-Yl)­amino)­azepane-1-carboxylate **20c**

Synthesis was performed by following the protocol described for **18c**. White solid (36%); ^1^H NMR (400 MHz, 25 degree, CDCl_3_) σ: 1.46 (9H, s), 1.55–1.73 (3H, m), 1.83–1.99 (2H, m), 2.10–2.14 (1H, m), 3.24 (1H, tdd, J = 15 Hz, 9 Hz, 3 Hz), 3.39–3.48 (2H, m), 3.51–3.68 (1H, m), 3.94 (1H, s), 5.78 (1H, s, br), 8.20 (2H, s); ^13^C NMR (101 MHz, 25 degree, CDCl_3_) σ: 24.5 (d, J = 16 Hz), 33.1 (d, J = 36 Hz), 35.0 (d, J = 4 Hz), 42.7 (d, J = 41 Hz), 52.08 (d, J = 24 Hz), 79.6, 105.5 (d, J = 8 Hz), 134.1 (d, J = 64 Hz), 145.5 (d, J = 23 Hz), 155.7; *m*/*z*: (ESI^+^) 311.2 ([M + H]^+^); *R*_*f*_: 0.6 (98:2 Chloroform: MeOH); Purity: ≥95%.

##### *tert*-Butyl 8-((5-Fluoropyrimidin-2-Yl)­amino)-2-azaspiro[4.5]­decane-2-carboxylate **17c**

Off-white solid (33%); ^1^H NMR (400 MHz, 25 degree, CDCl_3_) σ: 1.27–1.39 (2H, m), 1.45 (9H, s), 1.61–1.68 (4H, m), 1.74 (1H, t, J = 7 Hz), 1.97 (2H, t, J = 13 Hz), 3.15 (2H, t, J = 32 Hz), 3.34–3.40 (2H, m), 3.77 (2H, m), 5.22 (1H, s, br), 8.18 (2H, s); *m*/*z*: (ESI^+^) 295.1 ([M – *t*Bu + H]^+^); *R*_*f*_: 0.8 (1:1 DCM: Ethyl acetate); Purity: ≥95%.

#### General Procedure for Final Compounds Synthesis

Fluconazole core (1 equiv) and triethylamine (1.5 equiv) were added into the solution of amine (1.5 equiv) in ethanol (0.07 mmol/mL) under stirring. The mixture was heated under stirring at 80 °C overnight until all starting material disappeared. The solvent was removed under reduced pressure, and the product was purified by flash column chromatography (MeOH in EtOAc, 0–20%).

##### 2-(2,4-Difluorophenyl)-1-(4-(phenylamino)­piperidin-1-Yl)-3-(1h-1,2,4-triazol-1-Yl)­propan-2-Ol **1**

Light yellow liquid (58%); [α]­D24: 0 (c = 1.5 in methanol); ^1^H NMR (400 MHz, 25 degree, MeOH-d4) σ: 1.37–1.47 (2H, m), 1.80 (1H, d, J = 15 Hz), 1.91 (1H, d, J = 11 Hz), 2.25 (1H, td, J = 9 Hz, 3 Hz), 2.42 (1H, td, J = 2 Hz), 2.54–2.59 (1H, m), 2.73–2.78 (1H, m), 2.81 (1H, d, J = 13 Hz), 3.02 (1H, d, J = 14 Hz), 3.15–3.22 (1H, m), 4.58 (1H, d, J = 11 Hz), 4.68 (1H, d, J = 15 Hz), 6.56–6.61 (3H, m), 6.84 (1H, td, J = 9 Hz, 2 Hz), 6.90–6.96 (1H, m), 7.06 (2H, t, J = 8 Hz), 7.49 (1H, td, J = 8 Hz, 6 Hz), 7.75 (1H, s), 8.35 (1H, s); ^13^C NMR (101 MHz, 25 degree, MeOH-d4) σ: 33.2, 50.8, 54.9, 57.6, 64.4, 74.5 (d, J = 6 Hz), 104.9 (dd, J = 28 Hz, 26 Hz), 114.9, 118.2, 127.5 (dd, J = 14 Hz, 4 Hz), 130.1, 130.8 (dd, J = 10 Hz, 6 Hz), 146.1, 149.0, 151.1, 152.0, 161.1 (dd, J = 246 Hz, 12 Hz), 164.2 (dd, J = 248 Hz, 12 Hz); *m*/*z*: (ESI^+^) 414.2 ([M + H]^+^); HRMS: (ESI^+^) found 414.2088, ([M + H]^+^) requires 414.2100; *Rf*: 0.4 (Ethyl acetate); Purity: ≥99%.

##### 2-(2,4-Difluorophenyl)-1-(4-((4-fluorophenyl)­amino)­piperidin-1-Yl)-3-(1h-1,2,4-triazol-1-Yl)­propan-2-Ol **2**

Light yellow solid (50%); M.P. = 93–102 °C; [α]­D24: 14 (c = 0.7 in methanol); ^1^H NMR (400 MHz, 25 degree, MeOH-d4) σ: 2.38 (3H, m), 2.47–2.48 (4H, m), 2.79 (1H, d, J = 14 Hz), 2.99 (1H, dd, J = 14 Hz, 1 Hz), 3.43–3.50 (2H, m), 4.59 (1H, d, J = 14 Hz), 4.68 (1H, d, J = 14 Hz), 6.84 (1H, td, J = 8 Hz, 3 Hz), 6.88–6.94 (1H, m), 6.97 (1H, td, J = 8 Hz, 2 Hz), 7.06 (1H, dt, J = 10 Hz, 2 Hz), 7.09 (1H, d, J = 8 Hz), 7.30 (1H, td, J = 8 Hz, 7 Hz), 7.46 (1H, td, J = 9 Hz, 7 Hz), 7.74 (1H, s), 8.33 (1H, s); ^13^C NMR (101 MHz, 25 degree, MeOH-d4) σ: 54.1, 55.4, 57.5 (d, J = 5 Hz), 63.2 (d, J = 2 Hz), 64.5 (d, J = 3 Hz), 74.9 (d, J = 4 Hz), 104.9 (td, J = 28 Hz, 2 Hz), 112.0 (dd, J = 21 Hz, 3 Hz), 115.0 (d, J = 19 Hz), 117.0 (d, J = 20 Hz), 126.3 (d, J = 3 Hz), 127.2 (dd, J = 12 Hz, 4 Hz), 130.8 (dd, J = 3 Hz, 1 Hz), 131.0 (d, J = 8 Hz), 141.5 (d, J = 7 Hz), 146.1, 151.1, 159.7 (d, J = 28 Hz), 162.9 (dd, J = 246 Hz, 12 Hz), 164.3 (dd, J = 246 Hz, 12 Hz); *m*/*z*: (ESI^+^) 432.2 ([M + H]^+^); HRMS: (ESI^+^) found 432.1998, ([M + H]^+^) requires 432.2006; *Rf*: 0.4 (Ethyl acetate); Purity: ≥98%.

##### 2-(2,4-Difluorophenyl)-1-(4-(P-tolylamino)­piperidin-1-Yl)-3-(1h-1,2,4-triazol-1-Yl)­propan-2-Ol **3**

Transparent liquid (60%); [α]­D26: −4 (c = 2.5 in methanol); ^1^H NMR (400 MHz, 25 degree, MeOH-d4) σ: 1.25–1.44 (2H, m), 1.76–1.81 (1H, m), 1.87–1.91 (1H, m), 2.17 (3H, s), 2.23 (1H, td, J = 12 Hz, 3 Hz), 2.40 (1H, td, J = 12 Hz, 3 Hz), 2.53–2.57 (1H, m), 2.72–2.76 (1H, m), 2.80 (1H, d, J = 14 Hz), 3.00 (1H, dd, J = 14 Hz, 2 Hz), 3.13 (1H, tt, J = 10 Hz, 4 Hz), 4.57 (1H, d, J = 14 Hz), 4.67 (1H, d, J = 14 Hz), 6.55 (2H, ddd, J = 8 Hz, 3 Hz, 2 Hz), 6.84 (1H, tdd, J = 9 Hz, 3 Hz, 1 Hz), 6.89–6.95 (3H, m), 7.48 (1H, td, J = 9 Hz, 7 Hz), 7.74 (1H, s), 8.34 (1H, s); ^13^C NMR (101 MHz, 25 degree, MeOH-d4) σ: 20.5, 33.2, 33.5, 51.4, 54.9, 55.7, 57.6 (d, J = 5 Hz), 64.3 (d, J = 4 Hz), 74.4 (d, J = 6 Hz), 104.9 (dd, J = 28 Hz, 26 Hz), 112.0 (dd, J = 21 Hz, 3 Hz), 115.6, 127.5 (dd, J = 13 Hz, 4 Hz), 127.8, 130.5, 130.8 (dd, J = 10 Hz, 6 Hz), 146.1, 146.4, 151.1, 160.7 (dd, J = 246 Hz, 12 Hz), 164.1 (dd, J = 248 Hz, 12 Hz); *m*/*z*: (ESI^+^) 428.2 ([M + H]^+^); HRMS: (ESI^+^) found 428.2254, ([M + H]^+^) requires 428.22564; *Rf*: 0.4 (Ethyl acetate); Purity: ≥98%.

##### 2-(2,4-Difluorophenyl)-1-(4-(pyridin-2-Ylamino)­piperidin-1-Yl)-3-(1h-1,2,4-triazol-1-Yl)­propan-2-Ol **4**

Light yellow solid (52%); M.P. = 54–65 °C; [α]­D26: 0 (c = 1.2 in methanol); ^1^H NMR (400 MHz, 25 degree, MeOH-d4) σ: 1.33–1.52 (2H, m), 1.79 (1H, d, J = 12 Hz), 1.90 (1H, d, J = 12 Hz), 2.28 (1H, td, J = 12 Hz, 1 Hz), 2.44 (1H, td, J = 13 Hz, 2 Hz), 2.57 (1H, d, J = 11 Hz), 2.75 (1H, d, J = 10 Hz), 2.81 (1H, d, J = 15 Hz), 3.02 (1H, d, J = 14 Hz), 3.57 (1H, tt, J = 10 Hz, 3 Hz), 4.59 (1H, d, J = 11 Hz), 4.68 (1H, d, J = 9 Hz), 6.46–6.50 (2H, m), 6.84 (1H, td, J = 7 Hz, 4 Hz), 6.92 (1H, td, J = 10 Hz, 2 Hz), 7.37 (1H, td, J = 8 Hz, 3 Hz), 7.49 (1H, dd, J = 12 Hz, 8 Hz), 7.75 (1H, s), 7.86 (1H, d, J = 7 Hz), 8.35 (1H, s); ^13^C NMR (101 MHz, 25 degree, MeOH-d4) σ: 33.2, 33.4, 49.9, 54.9, 55.6, 57.6 (d, J = 7 Hz), 64.4 (d, J = 7 Hz), 74.5 (d, J = 8 Hz), 104.9 (dd, J = 28 Hz, J = 26 Hz), 110.4, 112.0 (dd, J = 21 Hz, 5 Hz), 113.0, 126.7 (dd, J = 17 Hz, 6 Hz), 130.8 (dd, J = 9 Hz, 5 Hz), 138.7, 146.1, 147.8, 151.1, 159.5, 160.7 (dd, J = 256 Hz, 12 Hz), 164.1 (dd, J = 248 Hz, 12 Hz); *m*/*z*: (ESI^+^) 415.2 ([M + H]^+^); HRMS: (ESI^+^) found 415.2039, ([M + H]^+^) requires 415.2052; *Rf*: 0.4 (10:1 Ethyl acetate: MeOH); Purity: ≥95%.

##### 2-(2,4-Difluorophenyl)-1-(4-((5-fluoropyridin-2-Yl)­amino)­piperidin-1-Yl)-3-(1h-1,2,4-triazol-1-Yl)­propan-2-Ol **5**

Light yellow solid (52%); M.P. = 59–74 °C; [α]­D24: 7.4 (c = 1.4 in methanol); (400 MHz, 25 degree, MeOH-d4) σ: 1.31–1.50 (2H, m), 1.79 (1H, d, J = 17 Hz), 1.90 (1H, d, J = 17 Hz), 2.26 (1H, td, J = 8 Hz, 8 Hz), 2.42 (1H, td, J = 13 Hz, 3 Hz), 2.56 (1H, d, J = 19 Hz), 2.74 (1H, d, J = 14 Hz), 2.81 (1H, d, J = 20 Hz), 3.01 (1H, d, J = 16 Hz), 3.54 (1H, tt, J = 11 Hz, 4 Hz), 4.60 (1H, d, J = 15 Hz), 4.68 (1H, d, J = 14 Hz), 6.46 (1H, dd, J = 11 Hz, 5 Hz), 6.84 (1H, td, J = 10 Hz, 4 Hz), 6.92 (1H, td, J = 11 Hz, 3 Hz), 7.22 (1H, td, J = 11 Hz, 2 Hz), 7.48 (1H, dd, J = 15 Hz, 12 Hz), 7.75 (1H, s), 7.76 (1H, m), 8.35 (1H, s); ^13^C NMR (101 MHz, 25 degree, MeOH-d4) σ: 33.1, 33.4, 49.9, 54.9, 55.6, 57.6 (d, J = 7 Hz), 64.4 (d, J = 6 Hz), 74.5 (d, J = 5 Hz), 104.9 (dd, J = 26 Hz, J = 23 Hz), 110.8 (d, J = 4 Hz), 112.0 (dd, J = 21 Hz, 5 Hz), 126.7 (d, J = 22 Hz), 127.4 (dd, J = 11 Hz, 6 Hz), 130.8 (dd, J = 12 Hz, 7 Hz), 134.0 (d, J = 19 Hz), 146.1, 151.1, 154.2 (d, J = 249 Hz), 156.7, 160.7 (dd, J = 253 Hz, 12 Hz), 164.1 (dd, J = 246 Hz, 12 Hz); *m*/*z*: (ESI^+^) 433.1 ([M + H]^+^); HRMS: (ESI^+^) found 433.1948, ([M + H]^+^) requires 433.1958; *Rf*: 0.1 (Ethyl acetate); Purity: ≥95%.

##### 2-(2,4-Difluorophenyl)-1-(4-(Pyrimidin-2-Ylamino)­piperidin-1-Yl)-3-(1h-1,2,4-triazol-1-Yl)­propan-2-Ol **6**

Light orange solid (52%); M.P. = 134–139 °C; [α]­D24: 20 (c = 1.0 in methanol); ^1^H NMR (400 MHz, 25 degree, MeOH-d4) σ: 1.39–1.58 (2H, m), 1.84 (2H, dd, J = 49 Hz, 11 Hz), 2.26 (1H, td, J = 10 Hz, 5 Hz), 2.44 (1H, td, J = 11 Hz, 4 Hz), 2.58 (1H, d, J = 8 Hz), 2.77 (1H, d, J = 9 Hz), 2.81 (1H, d, J = 19 Hz), 3.02 (1H, d, J = 22 Hz), 3.69 (1H, tt, J = 12 Hz, 3 Hz), 4.60 (1H, d, J = 9 Hz), 4.69 (1H, d, J = 16 Hz), 6.55 (1H, t, J = 4 Hz), 6.85 (1H, td, J = 12 Hz, 3 Hz), 6.93 (1H, td, J = 10 Hz, 3 Hz), 7.49 (1H, dd, J = 16 Hz, 8 Hz), 7.75 (1H, s), 8.21 (1H, s), 8.23 (1H, s), 8.36 (1H, s); ^13^C NMR (101 MHz, 25 degree, MeOH-d4) σ: 33.1, 32.9, 49.9, 54.9, 55.6, 57.6 (d, J = 5 Hz), 64.4 (d, J = 8 Hz), 74.6 (d, J = 7 Hz), 104.9 (dd, J = 33 Hz, 17 Hz), 111.2, 112.0 (dd, J = 24 Hz, 6 Hz), 127.4 (dd, J = 12 Hz, J = 4 Hz), 130.8 (dd, J = 10 Hz, 3 Hz), 146.1, 151.1, 159.3, 160.7 (dd, J = 250 Hz, 12 Hz), 162.85, 164.14 (dd, J = 248 Hz, 12 Hz); *m*/*z*: (ESI^+^) 416.2 ([M + H]^+^); HRMS: (ESI^+^) found 416.1994, ([M + H]^+^) requires 416.2005; *Rf*: 0.4 (9:1 Ethyl acetate: MeOH); Purity: ≥95%.

##### 2-(2,4-Difluorophenyl)-1-(4-((5-fluoropyrimidin-2-Yl)­amino)­piperidin-1-Yl)-3-(1h-1,2,4-triazol-1-Yl)­propan-2-Ol **7**

Pale yellow crystals (58%); M.P. = 119 °C; [α]­D23: 24 (c = 0.9 in methanol); ^1^H NMR (400 MHz, 25 degree, MeOH-d4) σ: 1.38–1.56 (2H, m), 1.79 (1H, d, J = 12 Hz), 1.90 (1H, d, J = 11 Hz), 2.25 (1H, td, J = 12 Hz, 1 Hz), 2.42 (1H, td, J = 12 Hz, 2 Hz), 2.57 (1H, d, J = 9 Hz), 2.75 (1H, d, J = 9 Hz), 2.81 (1H, d, J = 16 Hz), 3.01 (1H, d, J = 10 Hz), 3.64 (1H, tt, J = 9 Hz, 4 Hz), 4.59 (1H, d, J = 11 Hz), 4.68 (1H, d, J = 12 Hz), 6.82–6.96 (2H, m), 7.49 (1H, dd, J = 15 Hz, 8 Hz), 7.75 (1H, s), 8.17 (1H, s), 8.35 (1H, s); ^13^C NMR (101 MHz, 25 degree, MeOH-d4) σ: 32.8, 33.1, 49.3, 54.9, 55.6, 57.6 (d, J = 5 Hz), 64.3 (d, J = 3 Hz), 74.54 (d, J = 4 Hz), 104.9 (t, J = 24 Hz), 112.0 (dd, J = 21 Hz, 3 Hz), 127.4 (dd, J = 13 Hz, 4 Hz), 130.8 (dd, J = 8 Hz, 5 Hz), 146.6 (d, J = 21 Hz), 151.1, 152.0, 154.4, 160.4 (d, J = 1 Hz), 164.2 (dd, J = 246 Hz, 12 Hz), 160.7 (dd, J = 243 Hz, 12 Hz); *m*/*z*: (ESI^+^) 434.2 ([M + H]^+^); HRMS: (ESI^+^) found 434.1902, ([M + H]^+^) requires 434.1911; *Rf*: 0.2 (Ethyl acetate); Purity: ≥98%.

##### 2-(2,4-Difluorophenyl)-1-(4-((5-methylpyrimidin-2-Yl)­amino)­piperidin-1-Yl)-3-(1h-1,2,4-triazol-1-Yl)­propan-2-Ol **8**

Transparent liquid (51%); [α]­D24: 0 (c = 1.5 in methanol); ^1^H NMR (400 MHz, 25 degree, methanol-d4) σ: 1.23 (2H, t, J = 10 Hz), 1.87 (2H, t, J = 13 Hz), 2.12 (3H, s), 2.62 (1H, tt, J = 9 Hz, 4 Hz), 2.92 (1H, t, J = 12 Hz), 3.08 (1H, d, J = 12 Hz), 3.22 (1H, d, J = 12 Hz), 4.52 (2H, s), 4.65 (1H, d, J = 14 Hz), 4.72 (1H, d, J = 14 Hz), 6.86 (1H, t, J = 9 Hz), 6.94 (1H, t, J = 10 Hz), 7.48 (1H, dd, J = 14 Hz, 8 Hz), 7.78 (1H, s), 8.16 (2H, s), 8.34 (1H, s); ^13^C NMR (101 MHz, 25 degree, MeOH-d4) σ: 17.6, 32.9, 33.2, 54.9, 55.6, 57.6 (d, J = 5 Hz), 64.3 (d, J = 4 Hz), 74.5 (d, J = 6 Hz), 104.9 (dd, J = 28 Hz, 26 Hz), 112.0 (dd, J = 21 Hz, 3 Hz), 127.4 (dd, J = 13 Hz, 4 Hz), 130.8 (dd, J = 9 Hz, 6 Hz), 144.9 (d, J = 24 Hz), 146.1, 151.1, 151.9 (d, J = 244 Hz), 157.0 (d, J = 16 Hz), 160.7 (dd, J = 247 Hz, 12 Hz), 159.7 (d, J = 3 Hz), 164.1 (dd, J = 248 Hz, 12 Hz); *m*/*z*: (ESI^+^) 430.2 ([M + H]^+^); HRMS: (ESI^+^) found 430.2151, ([M + H]^+^) requires 430.2161; *Rf*: 0.4 (9:1 Ethyl acetate: MeOH); Purity: ≥98%.

##### 2-(2,4-Difluorophenyl)-1-(4-((4,6-dimethylpyrimidin-2-Yl)­amino)­piperidin-1-Yl)-3-(1h-1,2,4-triazol-1-Yl)­propan-2-Ol **9**

Light orange solid (52%); M.P. = 160–162 °C; [α]­D24: −11 (c = 0.9 in methanol); ^1^H NMR (400 MHz, 25 degree, methanol-d4) σ: 1.36–1.56 (2H, m), 1.76–1.82 (1H, m), 1.86–1.93 (1H, m), 2.23 (6H, s), 2.28 (1H, td, J = 11.4 Hz, 3 Hz), 2.44 (1H, td, J = 12 Hz, 3 Hz), 2.53–2.59 (1H, m), 2.72–2.77 (1H, m), 2.81 (1H, d, J = 14 Hz), 3.02 (1H, dd, J = 14 Hz, 2 Hz), 3.78 (1H, tt, J = 11 Hz, 4 Hz), 4.59 (1H, d, J = 14 Hz), 4.69 (1H, d, J = 14 Hz), 6.37 (1H, s), 6.85 (1H, tdd, J = 8 Hz, 3 Hz, 1 Hz), 6.93 (1H, ddd, J = 12 Hz, 9 Hz, 3 Hz), 7.49 (1H, td, J = 9 Hz, 7 Hz), 7.75 (1H, s), 8.36 (1H, s); ^13^C NMR (101 MHz, 25 degree, MeOH-d4) σ: 23.6, 33.1, 33.4, 54.9, 55.6, 57.6 (d, J = 5 Hz), 64.4 (d, J = 4 Hz), 75.6 (d, J = 6 Hz), 104.9 (dd, J = 28 Hz, 26 Hz), 110.3, 112.0 (dd, J = 21 Hz, 3 Hz), 127.4 (dd, J = 13 Hz, 4 Hz), 130.8 (dd, J = 9 Hz, 6 Hz), 146.1, 151.1, 160.7 (dd, J = 246 Hz, 12 Hz), 162.9, 164.2 (dd, J = 247 Hz, 12 Hz), 169.1; *m*/*z*: (ESI^+^) 444.2 ([M + H]^+^); HRMS: (ESI^+^) found 444.2311, ([M + H]^+^) requires 444.2318; *Rf*: 0.5 (9:1 Ethyl acetate: MeOH); Purity: ≥98%.

##### 2-(2,4-Difluorophenyl)-1-(4-((5-fluoro-4-methylpyrimidin-2-Yl)­amino)­piperidin-1-Yl)-3-(1h-1,2,4-triazol-1-Yl)­propan-2-Ol **10**

White solid (52%); M.P. = 79–130 °C; [α]­D24: 17 (c = 0.6 in methanol); ^1^H NMR (400 MHz, 25 degree, methanol-d4) σ: 1.38–1.56 (2H, m), 1.80 (1H, d, J = 12 Hz), 1.90 (1H, d, J = 12 Hz), 2.26 (1H, t, J = 11 Hz), 2.30 (3H, s), 2.43 (1H, t, J = 11 Hz), 2.58 (1H, d, J = 11 Hz), 2.76 (1H, d, J = 11 Hz), 2.82 (1H, d, J = 13 Hz), 3.03 (1H, d, J = 13 Hz), 3.66 (1H, tt, J = 10 Hz, 4 Hz), 4.60 (1H, d, J = 14 Hz), 4.70 (1H, d, J = 14 Hz), 6.86 (1H, td, J = 7 Hz, 2 Hz), 6.94 (1H, td, J = 9 Hz, 2 Hz), 7.50 (1H, dd, J = 16 Hz, 9 Hz), 7.77 (1H, s), 8.01 (1H, s), 8.37 (1H, s); ^13^C NMR (101 MHz, 25 degree, MeOH-d4) σ: 17.6, 32.9, 33.2, 54.9, 55.6, 57.6 (d, J = 5 Hz), 64.3 (d, J = 4 Hz), 74.5 (d, J = 6 Hz), 104.9 (dd, J = 28 Hz, 26 Hz), 112.0 (dd, J = 21 Hz, 3 Hz), 127.4 (dd, J = 13 Hz, 3 Hz), 130.8 (dd, J = 9 Hz, 6 Hz), 145.0 (d, J = 24 Hz), 146.1, 151.1, 151.9 (d, J = 244 Hz), 157.0 (d, J = 15 Hz), 159.7 (d, J = 3 Hz), 160.7 (dd. J = 246 Hz, 12 Hz), 164.2 (dd, J = 248 Hz, 12 Hz); *m*/*z*: (ESI^+^) 448.2 ([M + H]+); HRMS: (ESI^+^) found 448.2057, ([M + H]^+^) requires 448.2067; *Rf*: 0.3 (Ethyl acetate); Purity: ≥97%.

##### 2-(2,4-Difluorophenyl)-1-(4-(pyrimidin-5-Ylamino)­piperidin-1-Yl)-3-(1h-1,2,4-triazol-1-Yl)­propan-2-Ol **11**

Light yellowish solid (49%); M.P. = 141–198 °C; [α]­D26: 17 (c = 0.6 in methanol); ^1^H NMR (400 MHz, 25 degree, MeOH-d4) σ: 1.38–1.50 (2H, m), 1.87 (2H, dt, J = 37 Hz, 2 Hz), 2.31 (1H, td, J = 12 Hz, 4 Hz), 2.45 (1H, td, J = 12 Hz, 3 Hz), 2.62 (1H, d, J = 11 Hz), 2.78 (1H, H, J = 12 Hz), 2.83 (1H, d, J = 14 Hz), 3.04 (1H, dd, J = 12 Hz, 3 Hz), 3.29 (1H, tt, J = 12 Hz, 3 Hz), 4.60 (1H, d, J = 10 Hz), 4.69 (1H, d, J = 14 Hz), 6.85 (1H, td, J = 9 Hz, 3 Hz), 6.93 (1H, td, J = 10 Hz, 3 Hz), 7.49 (1H, td, J = 9 Hz, 7 Hz), 7.76 (1H, s), 8.09 (1H, s), 8.32 (1H, s), 8.35 (1H, s); ^13^C NMR (101 MHz, 25 degree, MeOH-d4) σ: 32.8, 54.5, 57.6, 64.3, 64.4, 74.7 (d, J = 5 Hz), 104.9 (dd, J = 28 Hz, 21 Hz), 112.0 (dd, J = 21 Hz, 4 Hz), 127.3 (dd, J = 13 Hz, 4 Hz), 130.8 (dd, J = 9 Hz, 6 Hz), 141.4, 143.7, 146.1, 146.8, 151.1, 160.7 (dd, J = 246 Hz, 12 Hz), 164.15 (dd, J = 245 Hz, 12 Hz); *m*/*z*: (ESI^+^) 416.2 ([M + H]^+^); HRMS: (ESI^+^) found 416.1994, ([M + H]^+^) requires 416.2005; *Rf*: 0.1 (9:1 Ethyl acetate: MeOH); Purity: ≥95%.

##### 2-(2,4-Difluorophenyl)-1-(4-((2-fluoropyrimidin-5-Yl)­amino)­piperidin-1-Yl)-3-(1h-1,2,4-triazol-1-Yl)­propan-2-Ol **12**

Light white liquid (54%); [α]­D25: 28 (c = 0.4 in methanol); ^1^H NMR (400 MHz, 25 degree, MeOH-d4) σ: 1.66–1.92 (2H, m), 2.17–2.34 (1H, m), 2.42 (1H, td, J = 11 Hz, 3 Hz), 2.53–2.60 (1H, m), 2.65–2.82 (2H, m), 3.02 (1H, dd, J = 14 Hz, 1 Hz), 3.15 (1H, tt, J = 10 Hz, 4 Hz), 3.87 (2H, s), 4.60 (1H, d, J = 7 Hz), 4.66 (1H, d, J = 4 Hz), 6.85 (1H, td, J = 8 Hz, 2 Hz), 6.92 (1H, ddt, J = 12 Hz, 9 Hz, 2 Hz), 7.49 (1H, dddd, J = 12 Hz, 7 Hz, 7 Hz, 3 Hz), 7.75 (1H, d, J = 4 Hz), 7.95 (2H, s), 8.35 (1H, s); ^13^C NMR (101 MHz, 25 degree, MeOH-d4) σ: 32.9, 35.1, 35.3, 55.2, 57.6 (d, J = 5 Hz), 64.3 (d, J = 4 Hz), 74.6 (d, J = 6 Hz), 104.9 (dd, J = 28 Hz, 26 Hz), 112.0 (dd, J = 21 Hz, 3 Hz), 127.4 (dd, J = 9 Hz, 4 Hz), 130.8 (dd, J = 10 Hz, 6 Hz), 138.9, 145.1, 146.1, 151.1, 159.5, 160.7 (dd, J = 246 Hz, 12 Hz), 164.13 (dd, J = 248 Hz, 12 Hz); *m*/*z*: (ESI^+^) 446.2; *Rf*: 0.3 (9:1 Ethyl acetate: MeOH); Purity: ≥95%.

##### 2-(2,4-Difluorophenyl)-1-(4-(5-fluoropyrimidin-2-Yl)­piperazin-1-Yl)-3-(1h-1,2,4-triazol-1-Yl)­propan-2-Ol **15**

Light yellow solid (67%); M.P. = 140–149 °C; [α]­D26: 0 (c = 0.8 in methanol); ^1^H NMR (400 MHz, 25 degree, MeOH-d4) σ: 2.51 (4H, td, J = 4 Hz, 3 Hz), 2.84 (1H, d, J = 14 Hz), 3.02 (dd, J = 14 Hz, 2 Hz), 3.65 (4H, t, J = 5 Hz), 4.65 (1H, d, J = 14 Hz), 4.72 (1H, d, J = 14 Hz), 6.87 (1H, tdd, J = 8 Hz, 3 Hz, 1 Hz), 6.94 (1H, ddd, J = 12 Hz, 9 Hz, 3 Hz), 7.50 (1H, td, J = 9 Hz, 7 Hz), 7.76 (1H, s), 8.23 (2H, d, J = 6 Hz), 8.35 (1H, s); ^1^³C NMR (101 MHz, 25 degree, MeOH-d4) σ: 45.5, 55.6, 57.5 (d, J = 5 Hz), 64.77 (d, J = 4 Hz), 75.3 (d, J = 6 Hz), 104.9 (dd, J = 28 Hz, 26 Hz), 112.0 (dd, J = 21 Hz, 3 Hz), 127.2 (dd, J = 13 Hz, 4 Hz), 130.9 (dd, J = 10 Hz, 6 Hz), 146.1, 146.3 (d, J = 22 Hz), 151.1, 153.2 (d, J = 247 Hz), 160.2 (d, J = 1 Hz), 160.7 (dd, J = 247 Hz, 12 Hz), 164.2 (dd, J = 248 Hz, 12 Hz); HRMS: (ESI^+^) found 420.1750, ([M + H]^+^) requires 420.17542; *Rf*: 0.4 (Ethyl acetate); Purity: ≥99%.

##### 2-(2,4-Difluorophenyl)-1-(4-((5-fluoropyrimidin-2-Yl)­amino)-[1,4’-bipiperidin]-1’-Yl)-3-(1h-1,2,4-triazol-1-Yl)­propan-2-Ol **16**

Yellowish liquid (50%); [α]­D27: 8 (c = 2.5 in methanol); ^1^H NMR (400 MHz, 25 degree, methanol-d4) σ: 1.45–1.93 (6H, m), 2.09–2.20 (3H, m), 2.35 (1H, td, J = 12 Hz, 2 Hz), 2.55–2.69 (4H, m), 2.79 (1H, d, J = 14 Hz), 2.90 (1H, m), 3.00 (1H, dd, J = 14 Hz, 1 Hz), 3.14–3.20 (2H, m), 3.81 (1H, tt, 10 Hz, 4 Hz), 4.60 (1H, d, J = 14 Hz), 4.7 (1H, d, J = 14 Hz), 6.86 (1H, ddd, J = 8 Hz, 8 Hz, 2 Hz), 6.93 (1H, ddd, J = 12 Hz, 9 Hz, 3 Hz), 7.49 (1H, ddd, J = 9 Hz, 9 Hz, 7 Hz), 7.76 (1H, s), 8.22 (1H, d, J = 1 Hz), 8.34 (1H, s); ^13^C NMR (101 MHz, 25 degree, MeOH-d4) σ: 28.6 (d, J = 30 Hz), 31.5, 55.0, 55.7, 57.5 (d, J = 5 Hz), 63.6, 64.0 (d, J = 4 Hz), 75.0 (d, J = 5 Hz), 79.3, 104.9 (dd, J = 28 Hz, 26 Hz), 112.03 (dd, J = 21 Hz, 3 Hz), 127.3 (dd, J = 12 Hz, 4 Hz), 130.9 (dd, J = 9 Hz, 6 Hz), 146.0, 146.7 (d, J = 22 Hz), 151.1, 153.4 (d, J = 246 Hz), 160.4 (d, J = 1 Hz), 160.6 (dd, J = 246 Hz, 12 Hz), 164.2 (dd, J = 247 Hz, 12 Hz); *m*/*z*: (ESI^+^) 517.2 ([M + H]+); HRMS: (ESI^+^) found 517.2643, ([M + H]^+^) requires 517.2646; *Rf*: 0.4 (9:1:0.1 Ethyl acetate: MeOH: TEA); Purity: ≥95%.

##### 2-(2,4-Difluorophenyl)-1-(8-((5-fluoropyrimidin-2-Yl)­amino)-2-azaspiro­[4.5]­decan-2-Yl)-3-(1h-1,2,4-triazol-1-Yl)­propan-2-Ol **17**

Transparent liquid (38%); [α]­D25: 36 (c = 0.6 in methanol); ^1^H NMR (400 MHz, 25 degree, MeOH-d4) σ: 1.11–1.25 (1H, m), 1.31–1.39 (3H, m), 1.51 (1H, td, J = 7 Hz, 2 Hz), 1.54–1.64 (3H, m), 1.82–1.85 (2H, m), 2.27 (1H, dd, J = 14 Hz, 9 Hz), 2.40 (1H, dd, J = 24 Hz, 9 Hz), 2.51–2.58 (2H, m), 2.95 (1H, dd, J = 13 Hz, 10 Hz), 3.05 (1H, ddd, J = 23 Hz, 13 Hz, 2 Hz), 3.58–3.66 (1H, m), 4.59 (1H, dd, J = 14 Hz, 2 Hz), 4.68 (1H, d, J = 14 Hz), 6.86 (1H, td, J = 8 Hz, 2 Hz), 6.93 (1H, dddd, J = 11 Hz, 9 Hz, 2 Hz, 2 Hz), 7.46–7.53 (1H, m), 7.76 (1H, d, J = 3 Hz), 8.18 (2H, s), 8.35 (1H, s); *m*/*z*: (ESI^+^) 488.2 ([M + H]^+^); HRMS: (ESI^+^) found 488.2382, ([M + H]^+^) requires 488.23802; *Rf*: 0.3 (Ethyl acetate); Purity: ≥96%.

##### 2-(2,4-Difluorophenyl)-1-(8-(5-fluoropyrimidin-2-Yl)-2,8-diazaspiro­[4.5]­decan-2-Yl)-3-(1h-1,2,4-triazol-1-Yl)­propan-2-Ol **18**

White solid (55%); M.P. = 110–112 °C; [α]­D24: 31 (c = 0.7 in methanol); ^1^H NMR (400 MHz, 25 degree, methanol-d4) σ: 1.48 (4H, t, J = 6 Hz), 1.61 (2H, t, J = 7 Hz), 2.37 (1H, d, J = 9 Hz), 2.43 (1H, d, J = 9 Hz), 2.62 (2H, t, J = 7 Hz), 2.98 (1H, d, J = 13 Hz), 3.10 (1H, dd, J = 13 Hz, 2 Hz), 3.58–3.70 (4H, m), 6.86 (1H, tdd, J = 8 Hz, 3 Hz, 1 Hz), 6.93 (1H, ddd, J = 12 Hz, 9 Hz, 3 Hz), 7.50 (1H, td, J = 9 Hz, 7 Hz), 7.76 (1H, s), 8.22 (2H, d, J = 1 Hz), 8.35 (1H, s); ^13^C NMR (101 MHz, 25 degree, MeOH-d4) σ: 37.4, 37.9 (d, J = 13 Hz), 41.7, 43.3 (d, J = 2 Hz), 55.8, 57.5 (d, J = 5 Hz), 62.8 (d, J = 4 Hz), 67.7, 75.2 (d, J = 6 Hz), 104.8 (dd, J = 28 Hz, 26 Hz), 112.0 (dd, J = 21 Hz, 3 Hz), 127.3 (dd, J = 13 Hz, 4 Hz), 131.1 (dd, J = 9 Hz, 6 Hz), 146.1, 146.2 (d, J = 22 Hz), 151.1, 152.9 (d, J = 246 Hz), 160.3 (d, J = 1 Hz), 160.8 (dd, J = 246 Hz, 12 Hz), 164.3 (dd, J = 247 Hz, 12 Hz); *m*/*z*: (ESI^+^) 474.2 ([M + H]+); HRMS: (ESI^+^) found 474.2220, ([M + H]^+^) requires 474.2224; *Rf*: 0.2 (Ethyl acetate); Purity: ≥95%.

##### 2-(2,4-Difluorophenyl)-1-(4-((5-fluoropyrimidin-2-Yl)­amino)­azepan-1-Yl)-3-(1h-1,2,4-triazol-1-Yl)­propan-2-Ol **20**

Yellowish liquid (40%); [α]­D26: −14 (c = 1.5 in methanol); ^1^H NMR (400 MHz, 25 degree, MeOH-d4) σ: 1.46–1.68 (4H, m), 1.81–1.89 (2H, m), 2.54–2.76 (4H, m), 2.90 (1H, dd, J = 14 Hz, 10 Hz), 3.23 (1H, ddd, J = 14 Hz, 3 Hz, 2 Hz), 3.93 (1H, dddd, J = 17 Hz, 13 Hz, 9 Hz, 4 Hz), 4.57 (1H, dd, J = 14 Hz, 7 Hz), 4.69 (1H, dd, J = 14 Hz, 1 Hz), 6.84 (1H, td, J = 8 Hz, 3 Hz), 6.89–6.96 (1H, m), 7.53 (1H, tdd, J = 9 Hz, 11 Hz, 7 Hz), 7.75 (1H, s), 8.19 (2H, s), 8.37 (1H, d, J = 9 Hz); ^13^C NMR (101 MHz, 25 degree, MeOH-d4) σ: 26.1 (d, J = 7 Hz), 34.1 (d, J = 8 Hz), 35.5 (d, J = 35 Hz), 52.3 (d, J = 30 Hz), 54.8 (d, J = 52 Hz), 57.6 (dd, J = 5 Hz, 4 Hz), 58.5 (d, J = 43 Hz), 65.3 (dd, J = 30 Hz, 3 Hz), 74.9 (dd, J = 16 Hz, 6 Hz), 104.9 (dd, J = 8 Hz, 2 Hz), 105.1 (d, J = 8 Hz), 112.0 (dd, J = 21 Hz, 3 Hz), 127.4 (dd, J = 13 Hz, 4 Hz), 131.2 (dd, J = 9 Hz, 6 Hz), 134.5 (d, J = 64 Hz), 140.2 (d, J = 116 Hz), 146.1 (d, J = 7 Hz), 146.6 (d, J = 22 Hz), 151.1 (d, J = 2 Hz), 153.1 (dd, J = 245 Hz, 1 Hz), 160.2 (dd, J = 5 Hz, 1 Hz), 164.2 (dd, J = 249 Hz, 12 Hz); *m*/*z*: (ESI^+^) 448.2 ([M + H]^+^); HRMS: (ESI^+^) found 448.2063, ([M + H]^+^) requires 448.20672; *Rf*: 0.2 (Ethyl acetate); Purity: ≥98%.

##### 2-(2,4-Difluorophenyl)-1-(4-(5-fluoropyrimidin-2-Yl)-1,4-diazepan-1-Yl)-3-(1h-1,2,4-triazol-1-Yl)­propan-2-Ol **21**

Transparent liquid (52%); [α]­D26: 15 (c = 0.7 in methanol); ^1^H NMR (400 MHz, 25 degree, methanol-d4) σ: 1.64–1.71 (2H, m), 2.54–2.69 (2H, m), 2.79 (2H, td, J = 6 Hz, 1 Hz), 2.88 (1H, d, J = 14 Hz), 3.20 (1H, dd, J = 14 Hz, 2 Hz), 3.62–3.76 (4H, m), 4.52 (1H, d, J = 14 Hz), 4.64 (1H, d, J = 14 Hz), 6.83 (1H, tdd, J = 9 Hz, 3 Hz, 1 Hz), 6.90 (1H, ddd, J = 12 Hz, 9 Hz, 3 Hz), 7.42 (1H, td, J = 9 Hz, 7 Hz), 7.75 (1H, s), 8.24 (2H, d, J = 1 Hz), 8.31 (1H, s); ^13^C NMR (101 MHz, 25 degree, MeOH-d4) σ: 27.9, 47.7, 57.4 (d, J = 5 Hz), 57.4, 57.5, 63.3 (d, J = 4 Hz), 75.1 (d, J = 6 Hz), 104.8 (dd, J = 28 Hz, 26 Hz), 112.0 (dd, J = 21 Hz, 3 Hz), 127.2 (dd, J = 13 Hz, 4 Hz), 131.0 (dd, J = 10 Hz, 6 Hz), 146.0, 146.4 (d, J = 22 Hz), 151.1, 153.0 (d, J = 245 Hz), 159.9 (d, J = 1 Hz), 160.6 (dd, J = 246 Hz, 12 Hz), 164.1 (dd, J = 248 Hz, 12 Hz); *m*/*z*: (ESI^+^) 434.1 ([M + H]^+^); HRMS: (ESI^+^) found 434.1907, ([M + H]^+^) requires 434.1911; *Rf*: 0.4 (Ethyl acetate); Purity: ≥96%.

### Purity Determination of Synthesized Final Compounds

The purity of the synthesized final compounds was evaluated by using LC–MS analysis with two gradient methods (Methods A and B). Analyses were performed on a Waters Alliance 2695 LC system with gradient elution, and UV detection was conducted by using a Waters 2996 photodiode array detector. A Monolithic C18 column (50 × 4.6 mm, Phenomenex) served as the stationary phase, and a gradient of water (mobile phase A) and acetonitrile (mobile phase B), both containing 0.1% formic acid, was used as the mobile phase. The injection volume was set to 10 μL. Compounds were dissolved in either H_2_O/acetonitrile (50:50, v/v) or DMSO/acetonitrile (50:50, v/v) depending on solubility. Peak areas corresponding to each compound were automatically calculated by the system software, and solvent-related UV signals were subtracted to determine the final purity. All compounds exhibited greater than 95% purity in both methods.

In Method A, the flow rate was 0.5 mL/min. The gradient began at 5% B, increased to 90% B at 3 min, and reached 95% B at 3.5 min. This was maintained for 1 min, followed by a return to initial conditions (95% A, 5% B) at 5 min.

In Method B, the flow rate was 1.0 mL/min. The gradient started at 5% B, held until 2 min, increased to 50% B at 5 min, and then ramped to 95% B at 7.5 min. This was maintained for 1.5 min before re-equilibrating to the initial 5% B by 10 min.

### Chiral HPLC Analysis

Samples were analyzed by reverse-phase chiral HPLC using isocratic methods with different ratios of water and acetonitrile (MeCN). 0.1% formic acid was added as an additive. Samples were run at 0.8 mL min^–1^ and monitored at 280 nm. A Daicel CHIRALPAK AD-H Analytical 250 × 4.6 mm, 5 μm column was used for this analysis. The flow rate was maintained at 0.8 mL min^–1^, and elutes were detected by the UV detector at a wavelength of 280 nm.

### Molecular Modeling

The protein structures of lanosterol 14-alpha demethylase from were obtained from the protein data bank (PDB ID 5TZ1), and lanosterol 14-alpha demethylase from (UniProt ID: A0A2H4QC40) was developed using AlphaFold3. AutoDock SMINA was initially used to identify the preferred binding pockets,^49^ and PyMOL was used in parallel to visualize all inhibitors’ or substrates’ binding poses. The parameters were kept at the default settings. After locating the most favored binding sites, GOLD was used for molecular docking of the drug molecules into the selected binding sites of target proteins. GOLD was used for final experiments due to its flexible docking and more reliable and precise binding energy and scoring estimation.

### MIC Susceptibility Tests

Microbroth dilution MICs were carried out by following EUCAST guidelines. A 96-well plate was filled with 100 μL of RPMI with 2% glucose in each well from column 2 to column 12. 200 μL of the compound diluted down in media from a DMSO stock was then added to the first column of the 96-well plate and diluted 2-fold by each column until column 11. Then, 100 μL of fungi strains from overnight cultures backdiluted to a starting concentration of ∼1 × 10^5^ CFU/mL was added to each well except a blank control row. The plate was incubated at 37 °C for 24 h in the incubator. Fungal growth was measured with a BMG plate reader (FLUOstar Microplate Reader, BMG Labtech) at OD530 nm. For azole antifungals, the MIC was defined as the lowest concentration that was able to inhibit ≥50% of the drug-free control. DMSO and fluconazole controls were run alongside. All MICs were conducted in triplicate or more until a modal MIC value was obtained or a range was stated if a modal value was not defined.

###  CYP51 Inhibition Study

The *in vitro* sterol 14α-demethylase activity of purified CYP51 (CaCYP51) and its inhibition by test compounds were assessed as previously described^45^ with minor modifications. The standard assay was performed in a total volume of 200 μL and contained 1.0 μM recombinant CaCYP51 and 2.0 μM cytochrome P450 reductase (CPR). Test inhibitors, including compound **7** and fluconazole, were dissolved in DMSO and added to the reaction mixture in a final volume of 2.5 μL, ensuring a final DMSO concentration not exceeding 1% (v/v) to avoid nonspecific effects on enzyme activity.

The enzymatic reaction was initiated by the addition of 4 mM NADPH and incubated at 37 °C with continuous shaking for 15 min. Reactions were quenched by rapid cooling on ice, and sterol metabolites were extracted by using an organic solvent system. The extracts were then dried and analyzed by gas chromatography–mass spectrometry (GC-MS) for the detection and quantification of demethylated sterol products. Each assay was performed in triplicate, and IC_50_ values were determined by plotting percent inhibition against log inhibitor concentration using nonlinear regression analysis.

### Time-Kill Assay

Overnight cultures of fungal strains were back-diluted to a starting concentration of ∼1 × 10^5^ CFU/mL into glass universals containing 3 mL of RPMI with 2% glucose media and the drug at a concentration of 4× MIC_50_. The glass universals were incubated at 37 °C while being shaken at 200 rpm for 24 h. Aliquots (20 μL) were taken out of the glass universals for each tested compound and nontreated (NT) control at six-time points (0, 1, 2, 4, 6, and 24 h), and Miles-Misra was performed to estimate the total number of colony-forming units (CFUs) per mL. The tests were conducted in triplicate, and the average value of log CFU/mL was reported. Along with the synthesized compounds, commercially available antifungal drugs were also tested for comparison. Tested compounds were defined as fungicidal if the loss of fungal population was more than a 3-log reduction in CFU/mL compared to time point 0 h.

### Biofilm Eradication Assay

Overnight cultures of fungal strains were back-diluted to a starting concentration of ∼1 × 10^5^ CFU/mL into 96-well plates in RPMI with 2% glucose and incubated for 24 h at 37 °C. The following day, the supernatant was carefully removed, biofilms were washed once with PBS, and then media was replaced with drug diutions, which were prepared in triplicate in separate plates. After a further 24 h incubation at 37 °C, biofilms were washed twice with PBS, fixed for 1 h at 80 °C, and then stained with 0.05% crystal violet stain for 15 min at room temperature. Crystal violet stain was then removed, biofilms were rinsed several times with dH_2_O, and then destained in 100% ethanol for 20 min. Absorbance was then measured on a CLARIOstar Plus platereader (BMG LabTech).

### Accumulation Assay and LC–MS/MS Method for Analysis of the Lystaes

The Hergenrother accumulation assay^1^ was performed in triplicate batches of five samples, with each batch containing fluconazole, voriconazole, or compound **7**. TDG 1912 was used as a model for compound accumulation in .

Minor adjustments were made to the protocol laid out by Geddes and Hergenrother. Yeast Peptone Digest (YPD) broth was used to culture , as this provided optimal growth.

After samples had been centrifuged in oil to remove excess compound, pellets were dissolved in 200 μL of DMSO and incubated at 37 °C while being shaken for 10 min to lyse the cells or release the intracellular contents. Miles Misra viable counts determined the number of colony-forming units (CFUs) for each replicate; this was completed for the 0.55 OD inoculum, postcompound incubation, solvent-only control, and lysates.

Quantification of fluconazole and compound **7** in cell lysates was performed by using an external calibration method. Stock solutions of each compound were prepared in methanol and used to generate calibration standards in a methanol:DMSO mixture (60:40, v/v). Lysate samples were diluted 1:20 in the same solvent mixture prior to analysis. A 5 μL aliquot of either a standard or diluted sample was injected for LC–MS/MS analysis.

Chromatographic separation of analytes was carried out using a Shimadzu Nexera XR ultrahigh-performance liquid chromatography (UHPLC) system, comprising two LC-20AD pumps, a DGC degasser, a SIL-30AC autosampler, a CBM-20A controller, and a column oven. The system was fitted with an Ascentis Express C18 column (5 cm × 2.1 mm, 2.7 μm particle size), and elution was performed using a binary solvent system: eluent A was 0.1% formic acid in water, and eluent B was 0.1% formic acid in methanol. The gradient program started at 5% B (0 min), increased to 85% B over 5 min, and returned to 5% B from 5 to 6 min for re-equilibration. The mobile phase was delivered at a flow rate of 0.21 mL/min. The column oven was maintained at 40 °C, and the autosampler was maintained at 10 °C.

The UHPLC system was coupled to a Shimadzu 8060 triple quadrupole mass spectrometer equipped with an electrospray ionization (ESI) source and operated in a positive ion mode. The instrument settings were as follows: nebulizing gas flow rate, 3.0 L/min; drying gas and heating gas flow rates, 10 L/min each; interface voltage, 4.5 kV; interface temperature, 300 °C; desolvation temperature, 526 °C; desolvation line temperature, 250 °C; and heat block temperature, 400 °C. Multiple reaction monitoring (MRM) transitions were used for quantification, with a dwell time of 100 ms per transition. Details of the specific MRM transitions for each compound are provided in Table S2.

The nonparametric Mann–Whitney test was used to determine a significant difference (*p* < 0.0001) between the median fluconazole and compound **7** concentrations

### Efficacy Assay in Model

*G. mellonella* larvae were injected with 10 μL of strain TDG1912 at ∼1 × 10^7^ CFU/mL into the first left proleg. Then, were injected with antimicrobial agent/10% DMSO in PBS in the first right proleg 30 min after infection. Controls were injected with PBS alone. Ten larvae were treated per condition, per repeat for a total of 30 larvae per condition across 3 independent repeats. were stored at 4 °C, allowed to come to room temperature for at least an hour before the procedure and were used within 2 weeks of the receipt date. were incubated at 37 °C and assessed for survival every day for 5 days. This method was adapted from Wand et al.^50^

###  survival test

Wild-type Oregon R (stock #4269) were obtained from the Bloomington Drosophila Stock Center and maintained in the laboratory for several generations. Flies were reared on Nutri-Fly Bloomington Formulation (Genesee Scientific) in bottles at 25 °C with 70% relative humidity under a 12:12 h light/dark cycle prior to infection. As reported previously,^51^ can tolerate liquid injection volumes of up to 32 nL. For each test compound and concentration, 10 nL was injected into 30 five-day-old, mated female adult flies at a consistent time of day by the same experimenter. Injections were administered into the dorsolateral thorax just below the right wing hinge using a handheld microinjector (Drummond Nanoject III) fitted with a fine glass needle. An average fly weight of 1 mg was assumed for dosing calculations. Following injection, flies were maintained at 30 °C—a temperature selected to balance proliferation with fly viability.^51^ Fly survival was monitored every 24 h over a 7-day period. This methodology was adapted from Glittenberg et al.^52^

### Efficacy Assay in Model

 adults were injected with 10 nL of strain TDG1912 at a concentration of 2 × 10^11^ CFU/mL (corresponding to ∼2 × 10^3^ yeast cells per fly) or 1 × 10^12^ CFU/mL (∼1 × 10^4^ yeast cells per fly) into the dorsolateral thorax below the right wing hinge. One hour postinfection, flies received a second injection at the same site with either the antimicrobial agent diluted in 10% DMSO/PBS or vehicle control (PBS alone). For each condition, 10 adult flies were treated per biological replicate, with three independent replicates conducted, totaling 30 flies per condition. Fly survival was monitored every 24 h for 7 days postinfection.

## Supplementary Material









## Notes

LC–MS method, NMR spectra, and HRMS (PDF)
Molecular modeling images, figures for and efficacy assays, and molecular formula strings (CSV)
(PDB)
(PDB)

## Abbreviations Used

AcOH: Acetic acid
CDC: Centers for Disease Control and Prevention
calLDM: Lanosterol 14α-demethylase of
carLDM: Lanosterol 14α-demethylase of
CLSI: Clinical and Laboratory Standards Institute
DCM: Dichloromethane
DIPEA: *N*, *N*-Diisopropylethylamine
DMA: Dimethylacetamide
DMSO: Dimethylsulfoxide
DMF: *N*,*N*-Dimethylformamide
EtN­(Pr-i)_2_: *N*,*N*-Diisopropylethylamine
ERB: Efflux resistant breaker
EtOH: Ethanol
Equiv.: Equivalent
EUCAST: European Committee on Antimicrobial Susceptibility Testing
Et_3_N: Triethylamine
ESI: Electrospray ionization
Fluc: Fluconazole
HCl: Hydrochloric acid
Hz: Hertz
HPLC: High-pressure liquid chromatography
J: Coupling constants
KO*t*Bu: Potassium *tert*-butoxide
K_2_CO_3_: Potassium carbonate
LDM: lanosterol 14α-demethylase
LogP: Lipophilicity
LC–MS: Liquid chromatography–mass spectroscopy
MIC: Minimum inhibitory concentration
MeOH: Methanol
MeCN: Acetonitrile
MgSO_4_: Magnesium sulfate
*m*/*z*: Mass-to-charge ratio peaks
NaBH­(OAc)_3_: Sodium triacetoxyborohydride
NaO*t*Bu: Sodium *tert*-butoxide
NaOH: Sodium hydroxide
Na­(OAc)_3_BH: Sodium triacetoxyborohydride
NMR: Nuclear magnetic resonance
ORD: Optical rotatory dispersion
Pd_2_(dba)_3_: Tris­(dibenzylideneacetone)­dipalladium (0)
PBS: Phosphate-buffered saline
ppm: parts per million
rac-BINAP: 2,2′-bis­(diphenylphosphino)-1,1′-binaphthyl
Rf: Retention factors
SAR: Structure–activity relationship
SNAr: Nucleophilic aromatic substitution
TEA: Triethylamine
TLC: Thin-layer chromatography
UV: Ultraviolet
Vori: Voriconazole
YPD: Yeast peptone digest
WHO: World Health Organization
Δ*G*: Delta G
