# Supplementary material for: New Generation Modified Azole Antifungals against Multidrug-Resistant
Source: J Med Chem. 2025 Jun 23;68(13):14054–71. doi: 10.1021/acs.jmedchem.5c01253 (PMC12257509; doi:10.1021/acs.jmedchem.5c01253)
Supplement: Supplementary file 1 [file jm5c01253_si_001.pdf]

Supporting Information

New generation modified azole antifungals against multidrug-resistant *Candida auris*

Yiyuan Chen[1], Yunxiao Li[1], Kazi S. Nahar[1,3], Md. Mahbub Hasan[1, 4], Caleb Marsh[2], Melanie Clifford[2], Godwin A. Aleku[1], Steven L. Kelly[5], David C. Lamb[5], Chengetai Diana Mpamhanga[6], Ilias Kounatidis[6], Ajit J. Shah[3], Charlotte K. Hind[2]*, J. Mark Sutton[1, 2] *, Khondaker Miraz Rahman[1] *

[1]Institute of Pharmaceutical Science, King's College London, 150 Stamford Street, London. SE1 9NH, United Kingdom.

[2]UK Health Security Agency, Countermeasures, Development, Evaluation and Preparedness, Manor Farm Road, Salisbury. SP4 0JG, United Kingdom.

[3]Department of Natural Sciences, University of Middlesex, The Burroughs, Hendon, London NW4 4BT, United Kingdom

[4]Department of Genetic Engineering and Biotechnology, Faculty of Biological Sciences, University of Chittagong, Chattogram - 4331, Bangladesh

[5]Centre for Cytochrome P450 Biodiversity, Faculty of Medicine, Health and Life Science, Swansea University, Swansea, SA2 8PP, United Kingdom

[6]School of Life Health and Chemical Sciences, The Open University, Milton Keynes, MK7 6AE, United Kingdom

Corresponding authors.

KMR: e-mail k.miraz.rahman@kcl.ac.uk, JMS: e-mail mark.sutton@ukhsa.gov.uk,

CH: e-mail charlotte.hind@ukhsa.gov.uk,

Content:

Antifungal activity (µg/mL) of fluconazole and voriconazole against Candida spp.	S2
The m/z of precursor, product ion and collision energy used for detection of each compound used in the accumulation assay	S3
NMR and MS Spectra of Final Compounds	S4
Representative Example of HPLC Profile of Some Compounds	S96
Chiral HPLC Analysis of Compound 7	S100
3D binding interactions for fluconazole and compound 7 with lanosterol 14α-demethylase (LDM) enzyme in C. auris.	S101
Efficacy of amphotericin B (Amp B) against C. auris TDG1912 infection in G. mellonella model	S102
Efficacy of modified azole compound **7** and fluconazole against *C. auris* TDG1912 infection in *D. melanogaster* model	S104

Table S1. Antifungal activity (µg/mL) of fluconazole and voriconazole against *Candida Spp.,* and their corresponding LogP value.

	**Log P**	***C. albicans NCPF3281***	***C. glabrata NCPF8018***	***C. tropicalis NCPF8760***	***C. parapsilosis NCPF3209***	***C. auris***							
						TDG1912	***TDG2512***	***TDG1102***	***TDG2211***	***TDG2506***	***NCPF8984***	***NCPF8971***	***NCPF8977***
Fluconazole	0.87	0.06 – 0.125	4	16	0.25	> 128	8	64	64	128	128	32	16
Voriconazole	2.28	≤0.007	0.125	0.5	≤0.007	1	0.125	4	0.5	1	1	0.25	0.03-0.06

Table S2. The *m/z* of precursor, product ion and collision energy used for detection of each compound used in the accumulation assay.

Compound	Precursor *m/z*	Product *m/z*	Collision Energy (eV)
Fluconazole	307.3	169.1	-22
	307.3	220.1*	-19
	307.3	238.1	-16
Compound **7**	434.4	127.0	-49
	434.4	321.2*	-21
	434.4	365.1	-22

*Transition used for quantitation the other two product ions were used as qualifiers

NMR and MS Spectra of Final Compounds

2-(2,4-difluorophenyl)-1-(4-(phenylamino)piperidin-1-yl)-3-(1H-1,2,4-triazol-1-yl)propan-2-ol (1)

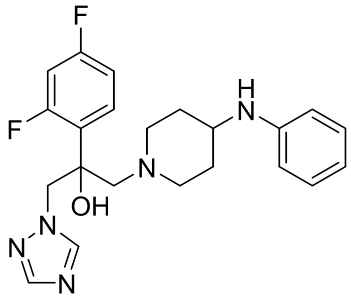

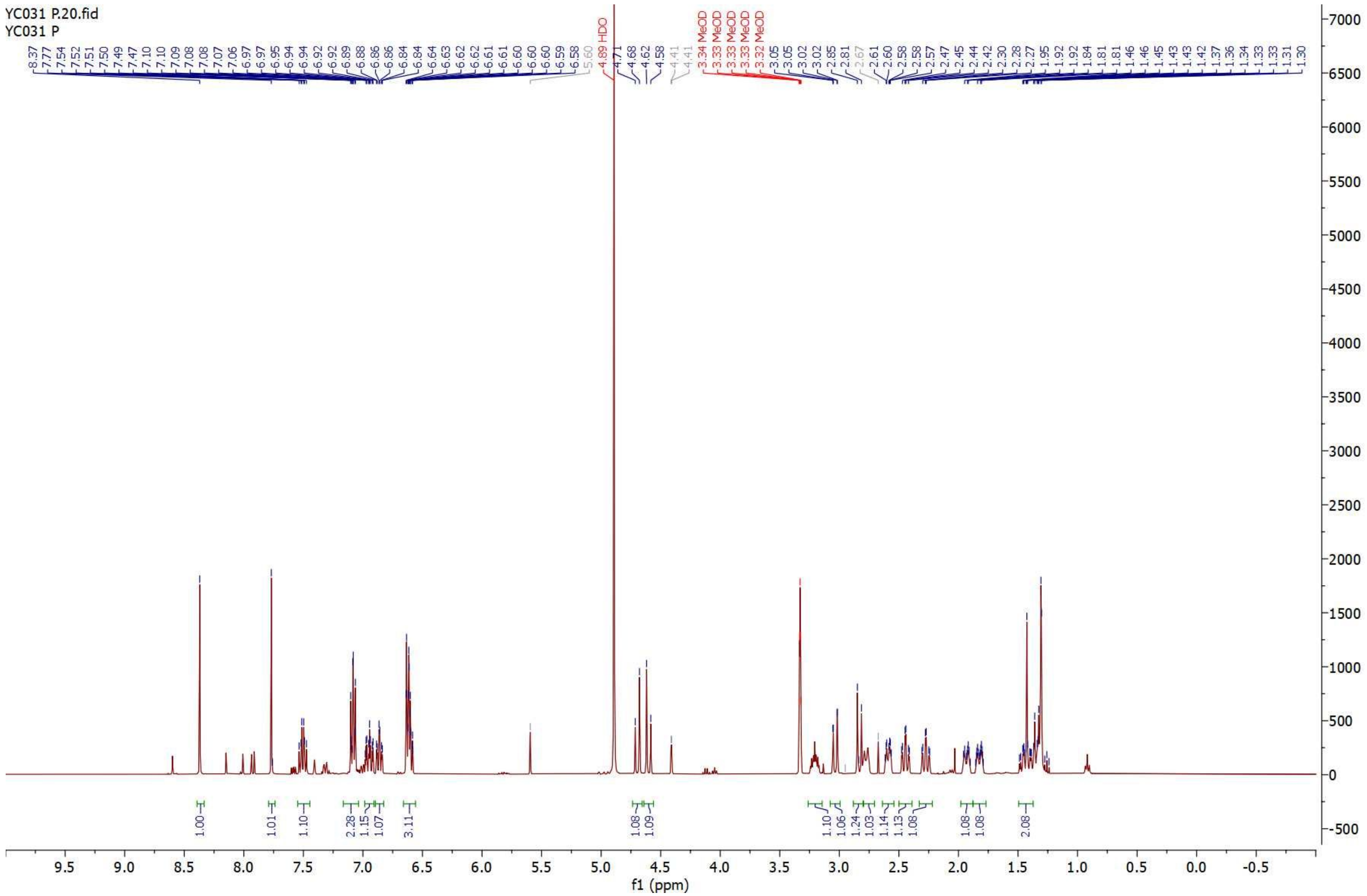

Figure S1-1: Proton ^{1}H NMR spectrum of Compound **1.**

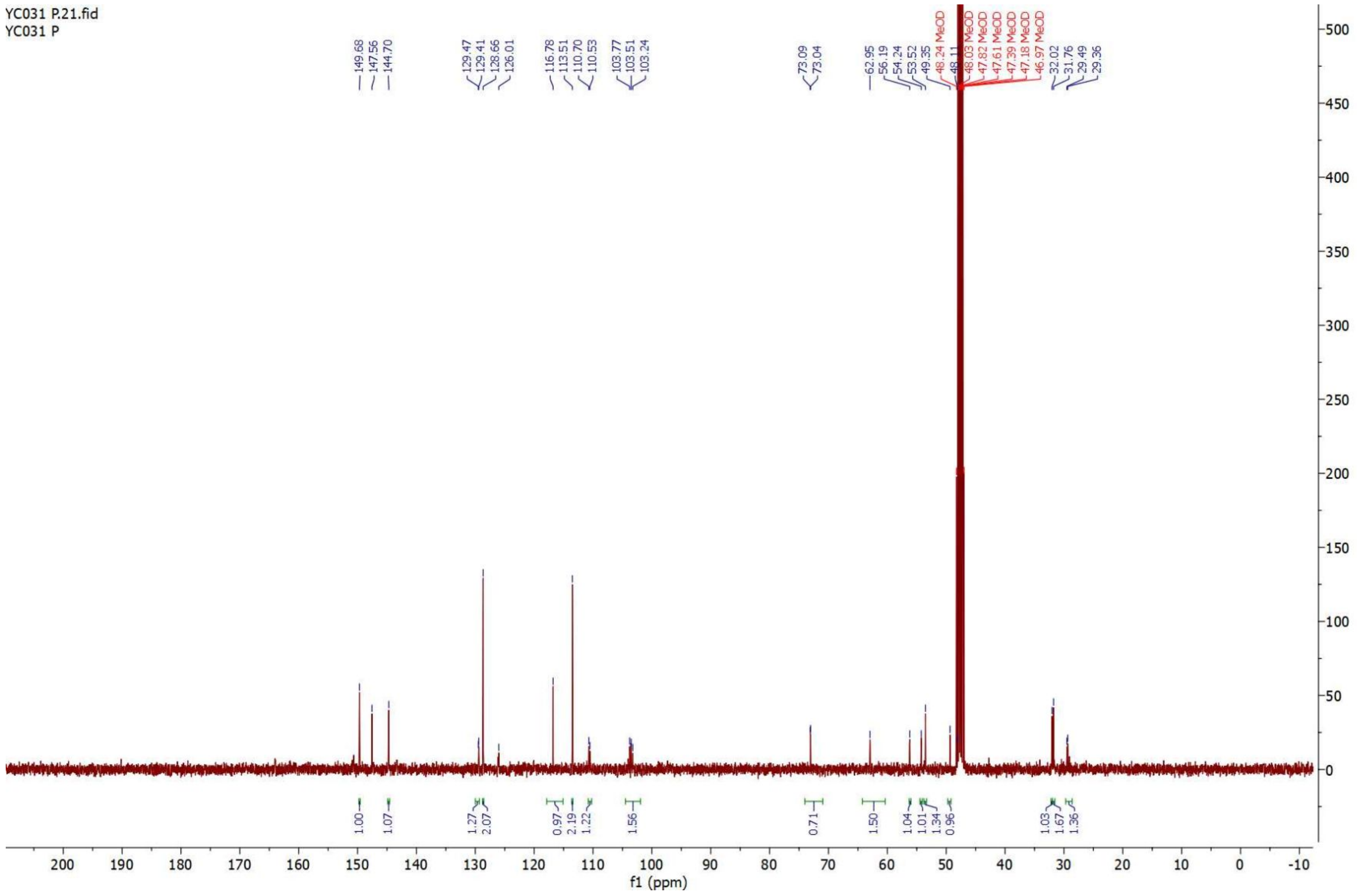

Figure S1-2: Carbon ^{13}C NMR spectrum of Compound **1.**

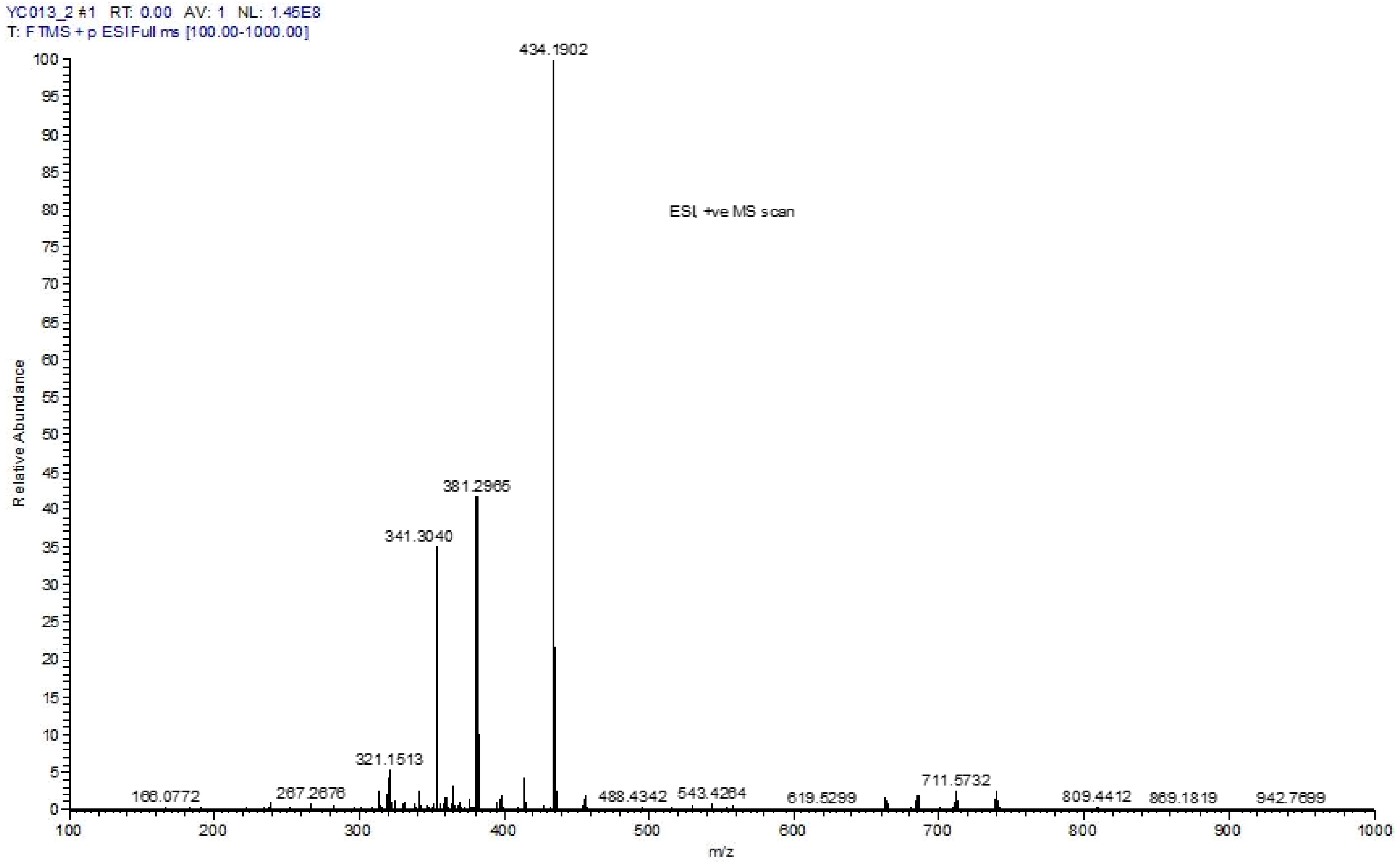

Figure S1-3: HRMS spectrum of Compound **1.**

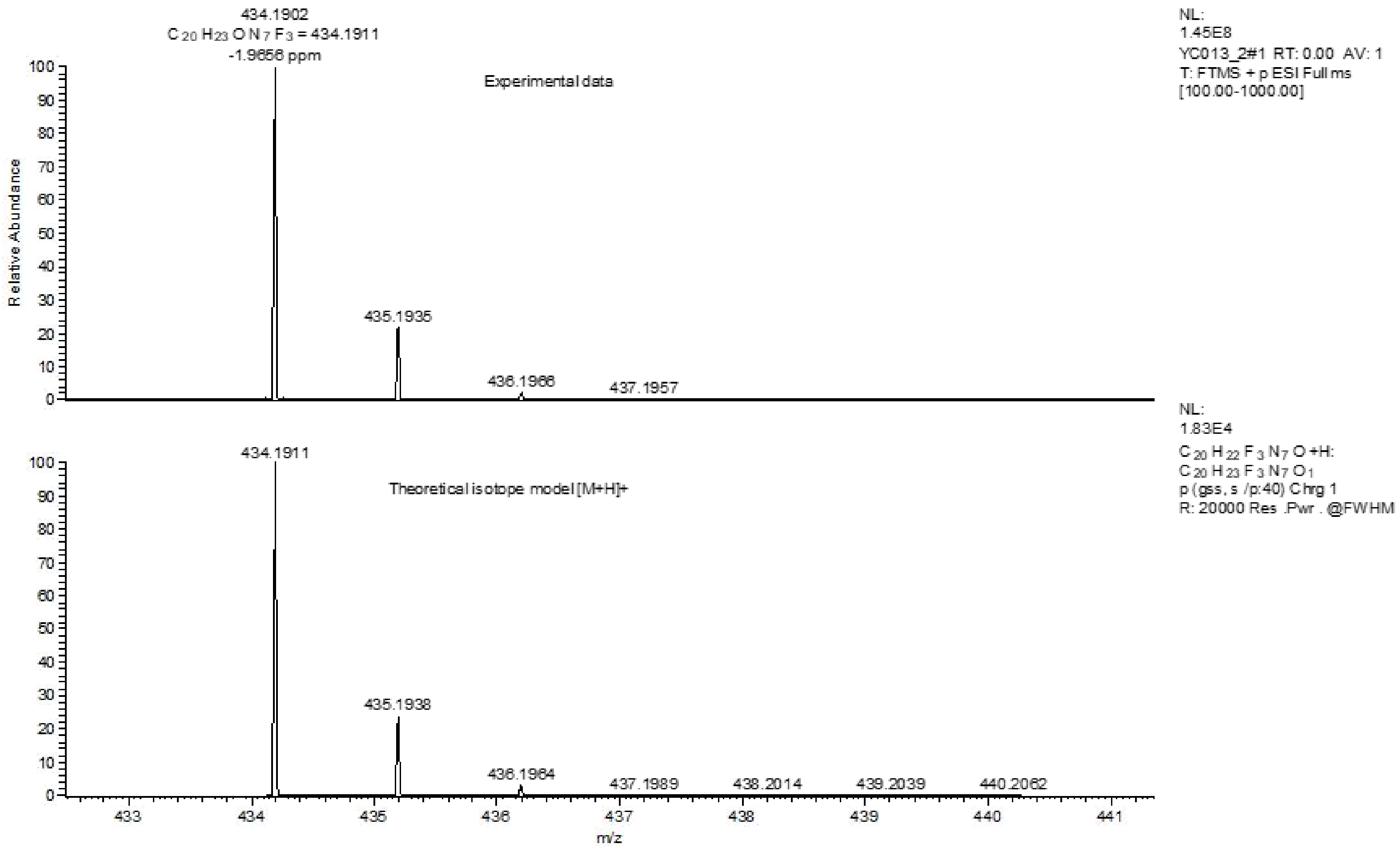

Figure S1-4: HRMS spectrum of Compound **1.**

2-(2,4-difluorophenyl)-1-(4-((4-fluorophenyl)amino)piperidin-1-yl)-3-(1H-1,2,4-triazol-1-yl)propan-2-ol (2)

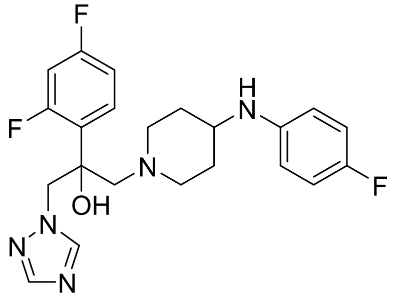

F
F
OH
N
N
N
N
H
N
F

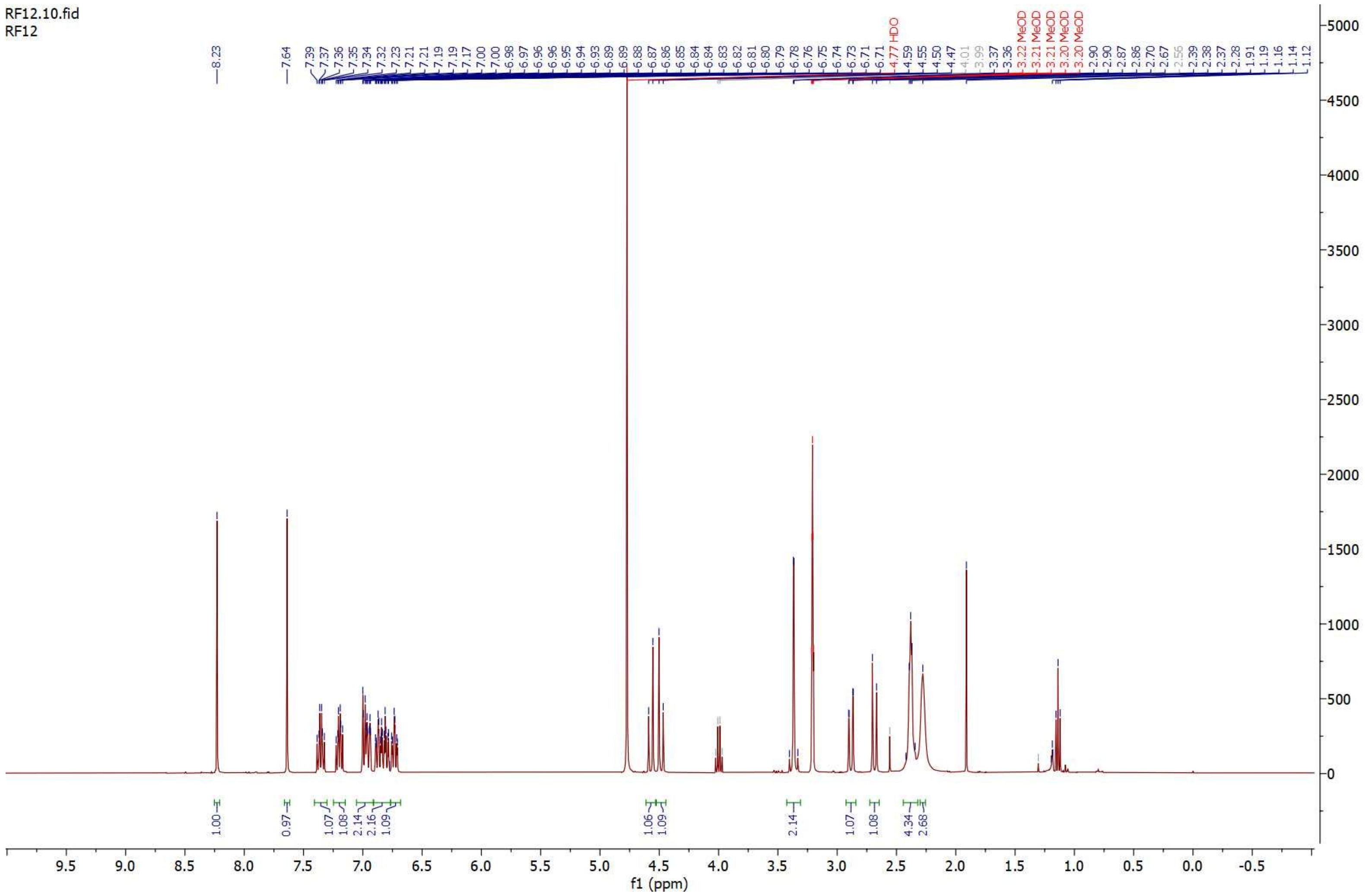

Figure S2-1: Proton ^{1}H NMR spectrum of Compound **2.**

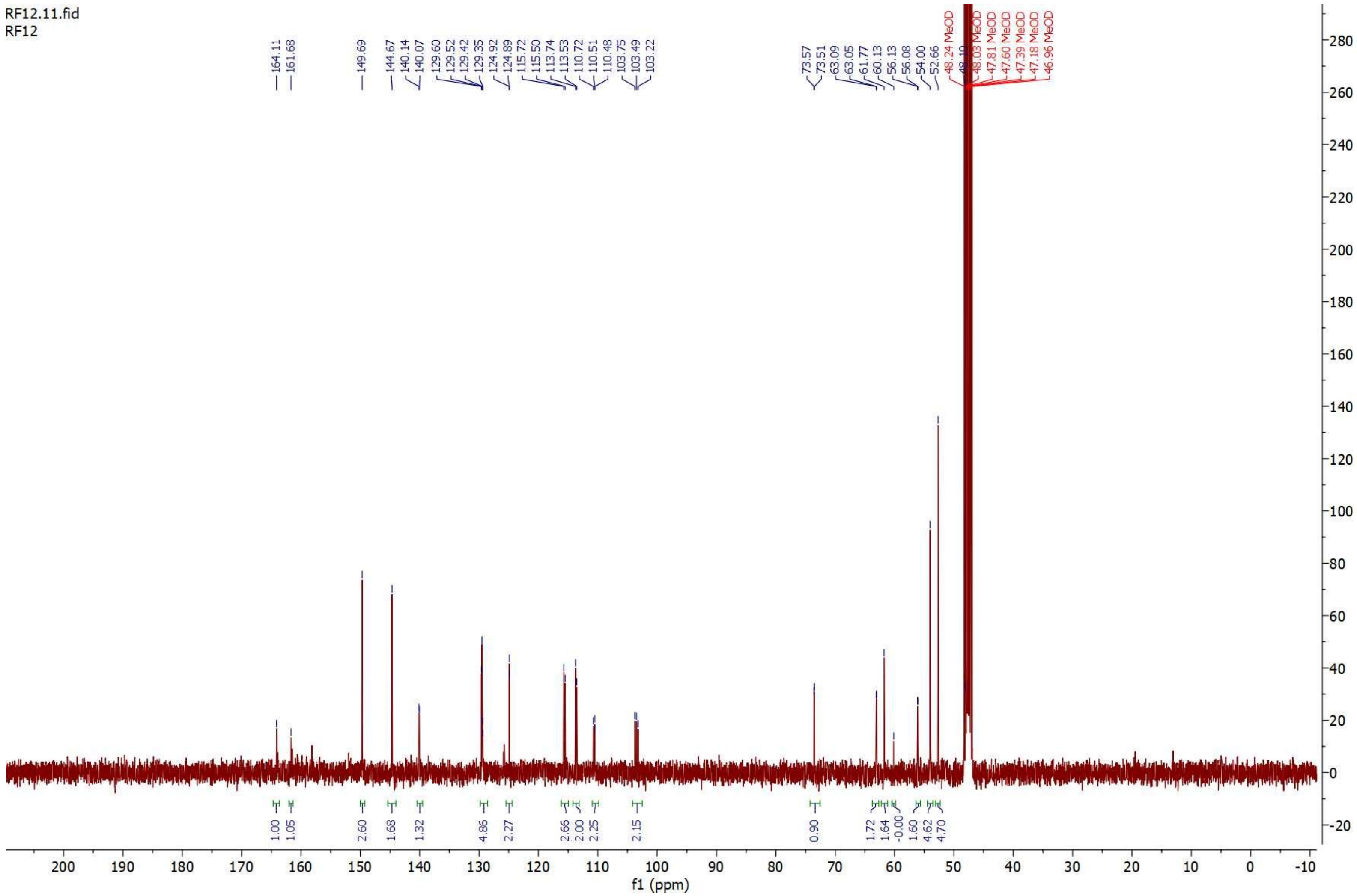

Figure S2-2: Carbon ^{13}C NMR spectrum of Compound **2.**

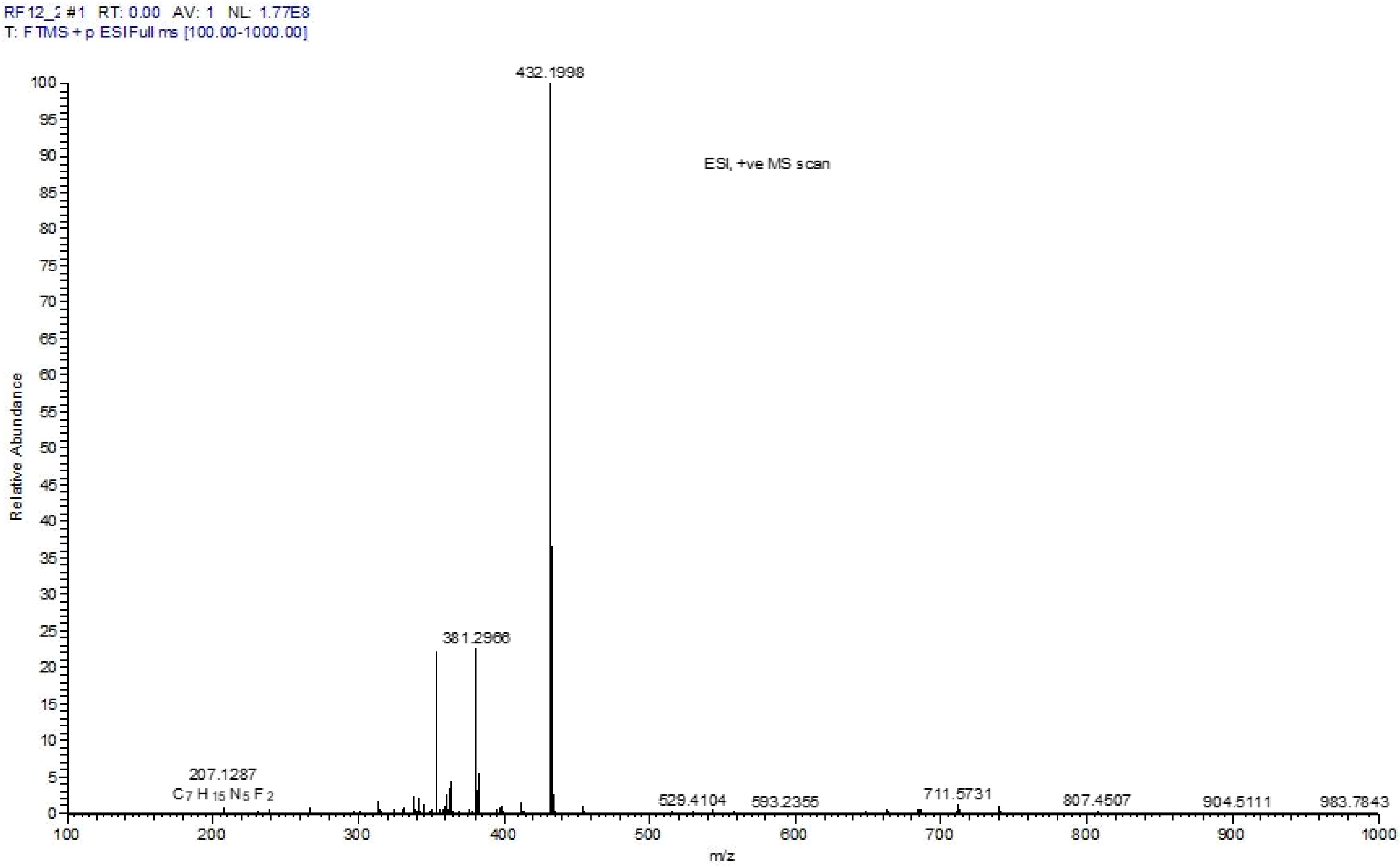

Figure S2-3: HRMS spectrum of Compound **2.**

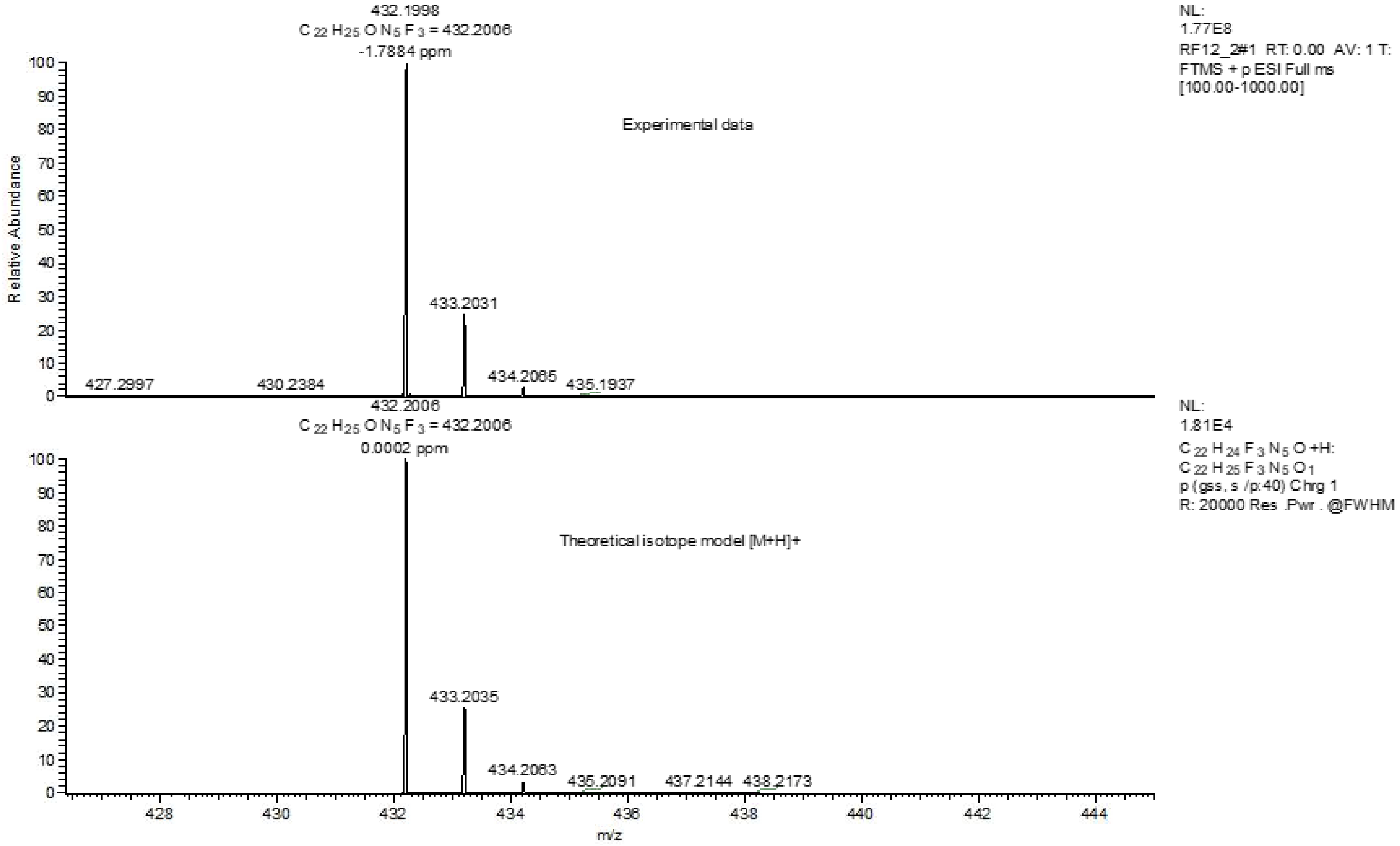

Figure S2-4: HRMS spectrum of Compound **2.**

2-(2,4-difluorophenyl)-1-(4-(p-tolylamino)piperidin-1-yl)-3-(1H-1,2,4-triazol-1-yl)propan-2-ol (3)

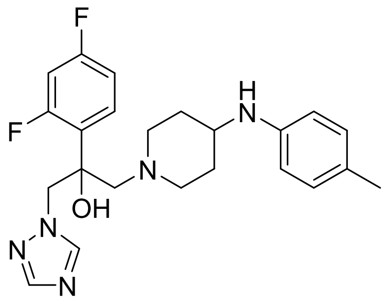

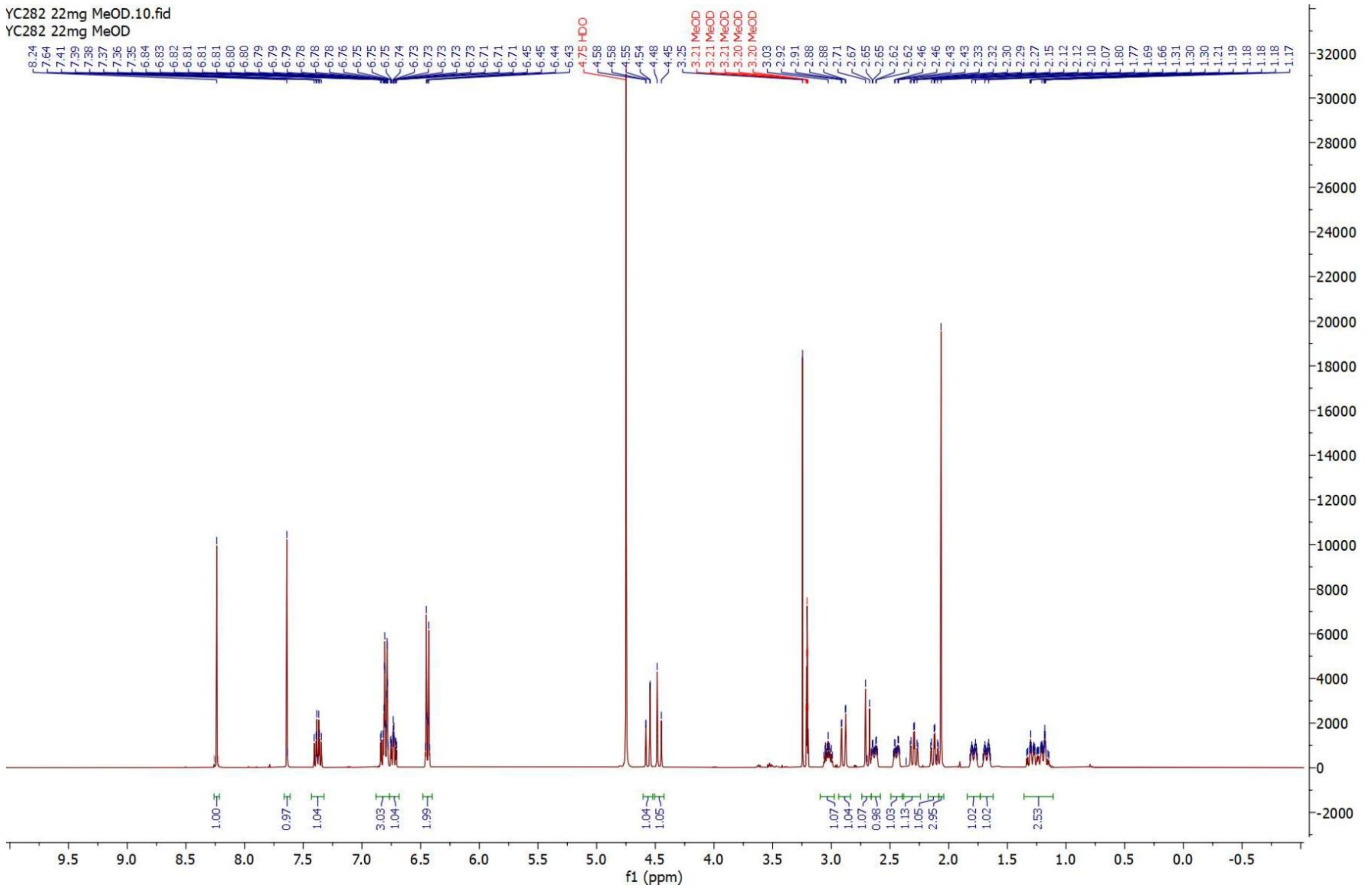

Figure S3-1: Proton ^{1}H NMR spectrum of Compound **3.**

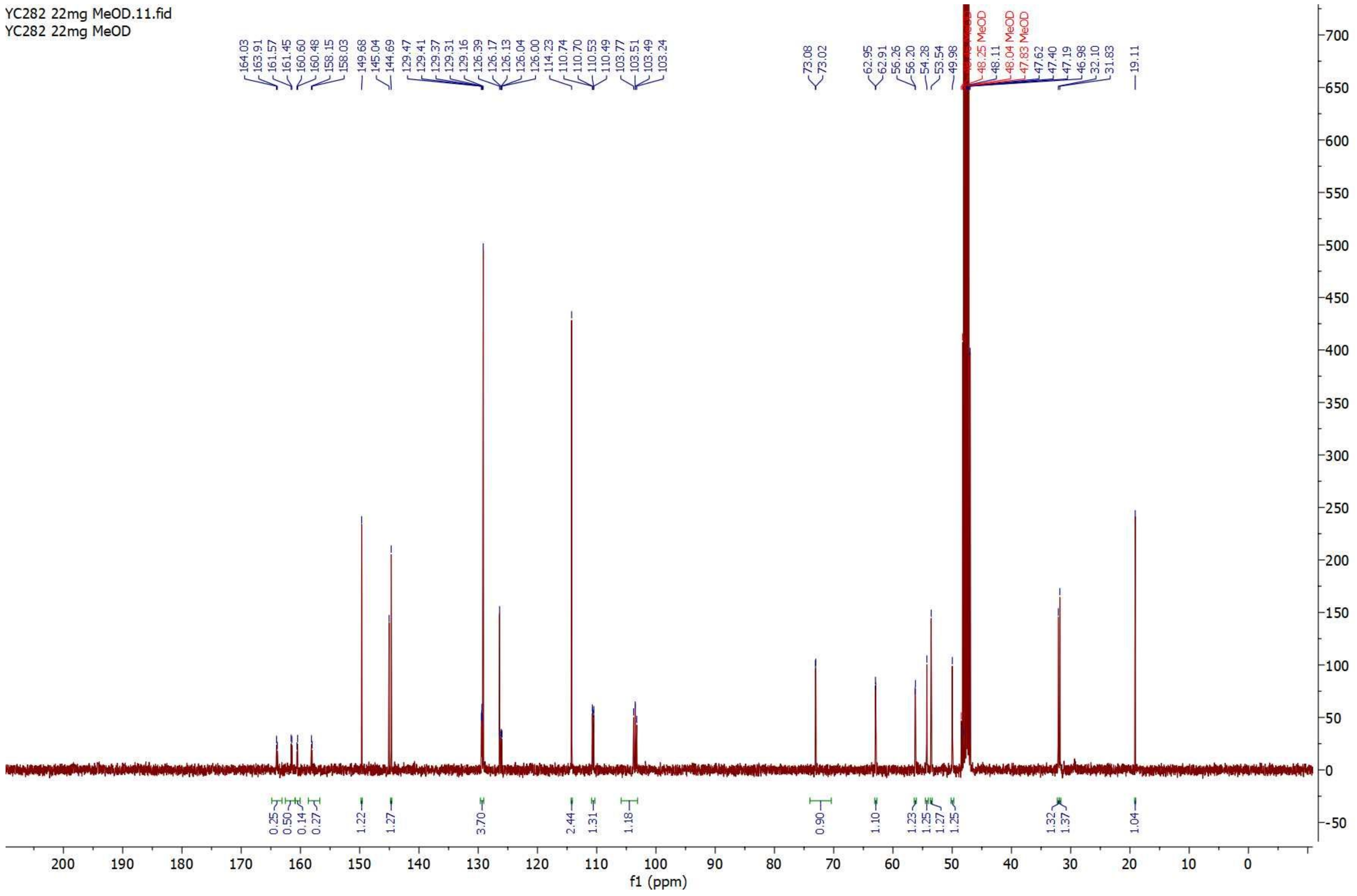

Figure S3-2: Carbon ^{13}C NMR spectrum of Compound **3.**

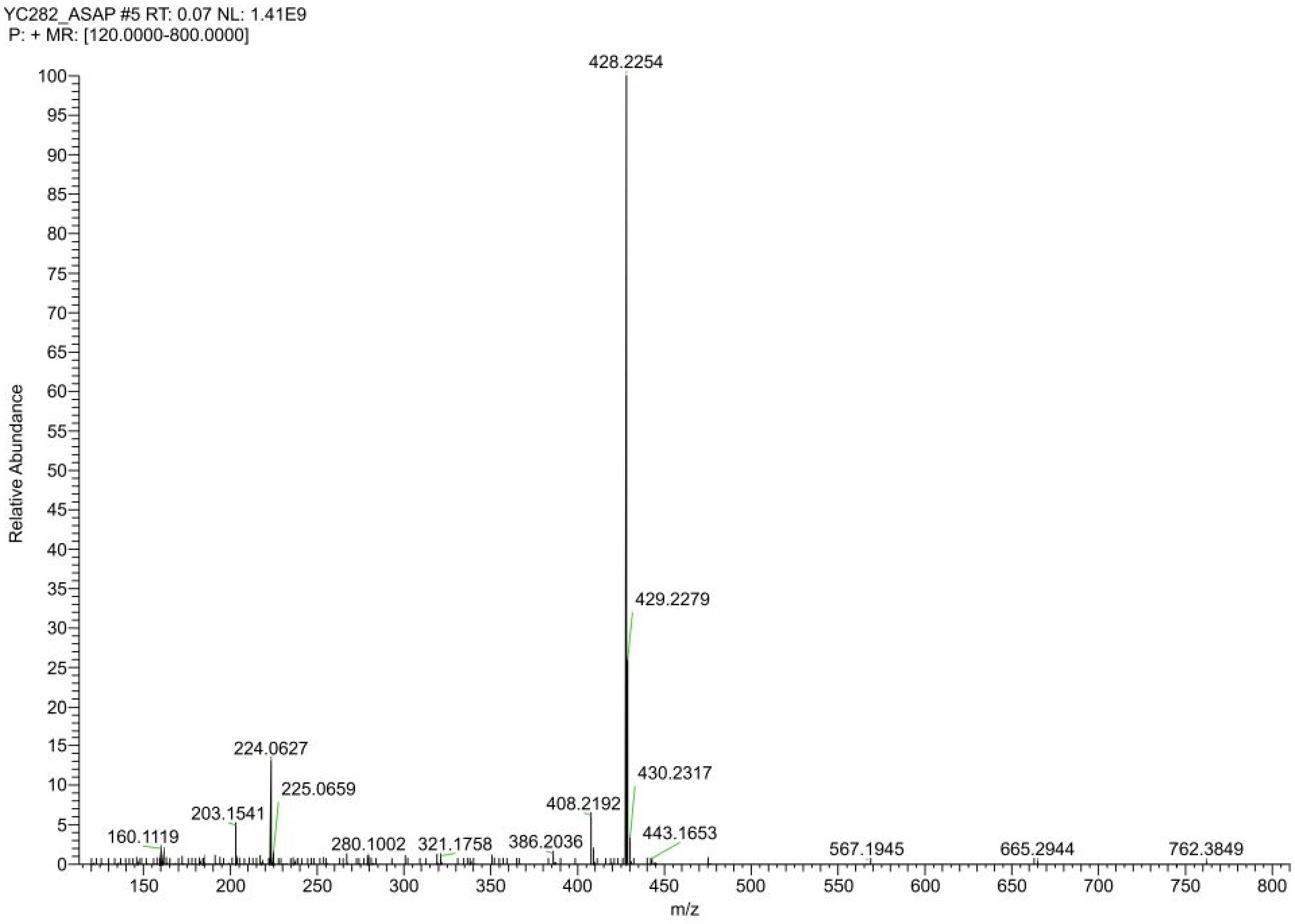

Figure S3-3: HRMS spectrum of Compound **3.**

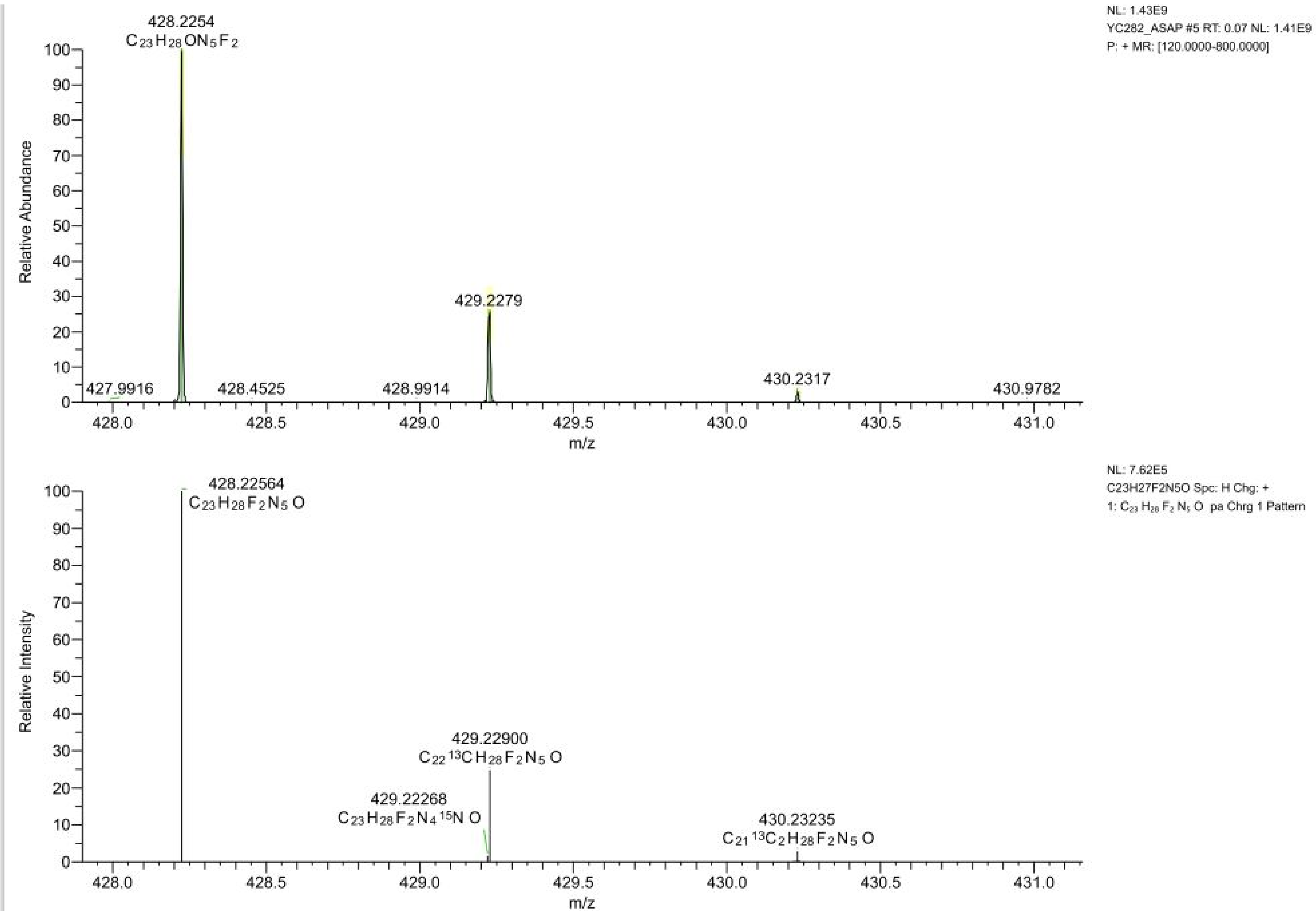

Figure S3-4: HRMS spectrum of Compound **3.**

2-(2,4-difluorophenyl)-1-(4-(pyridin-2-ylamino)piperidin-1-yl)-3-(1H-1,2,4-triazol-1-yl)propan-2-ol (4)

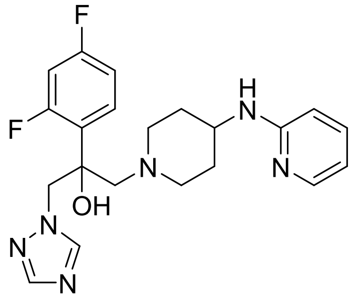

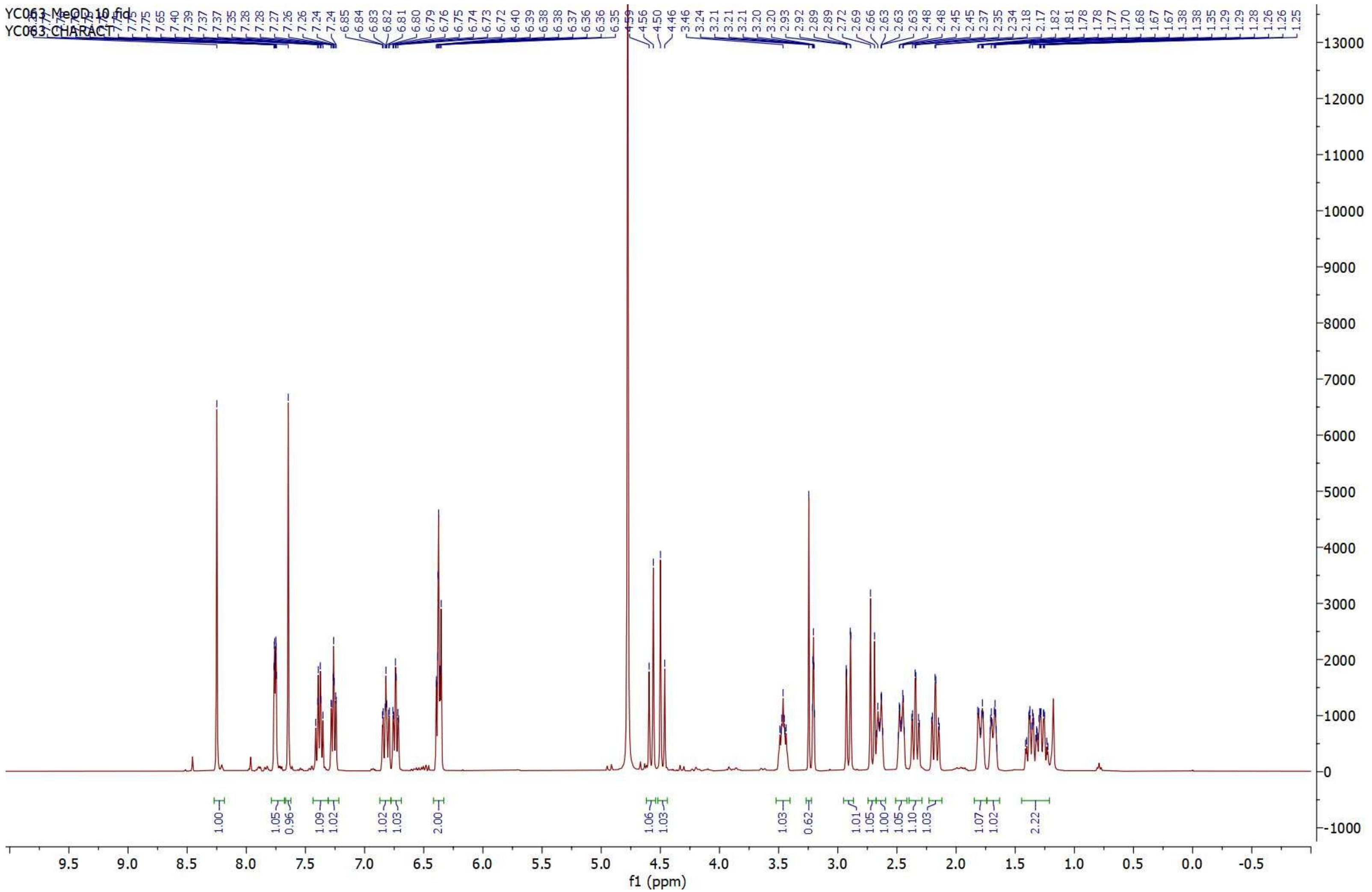

Figure S4-1: Proton ^{1}H NMR spectrum of Compound **4.**

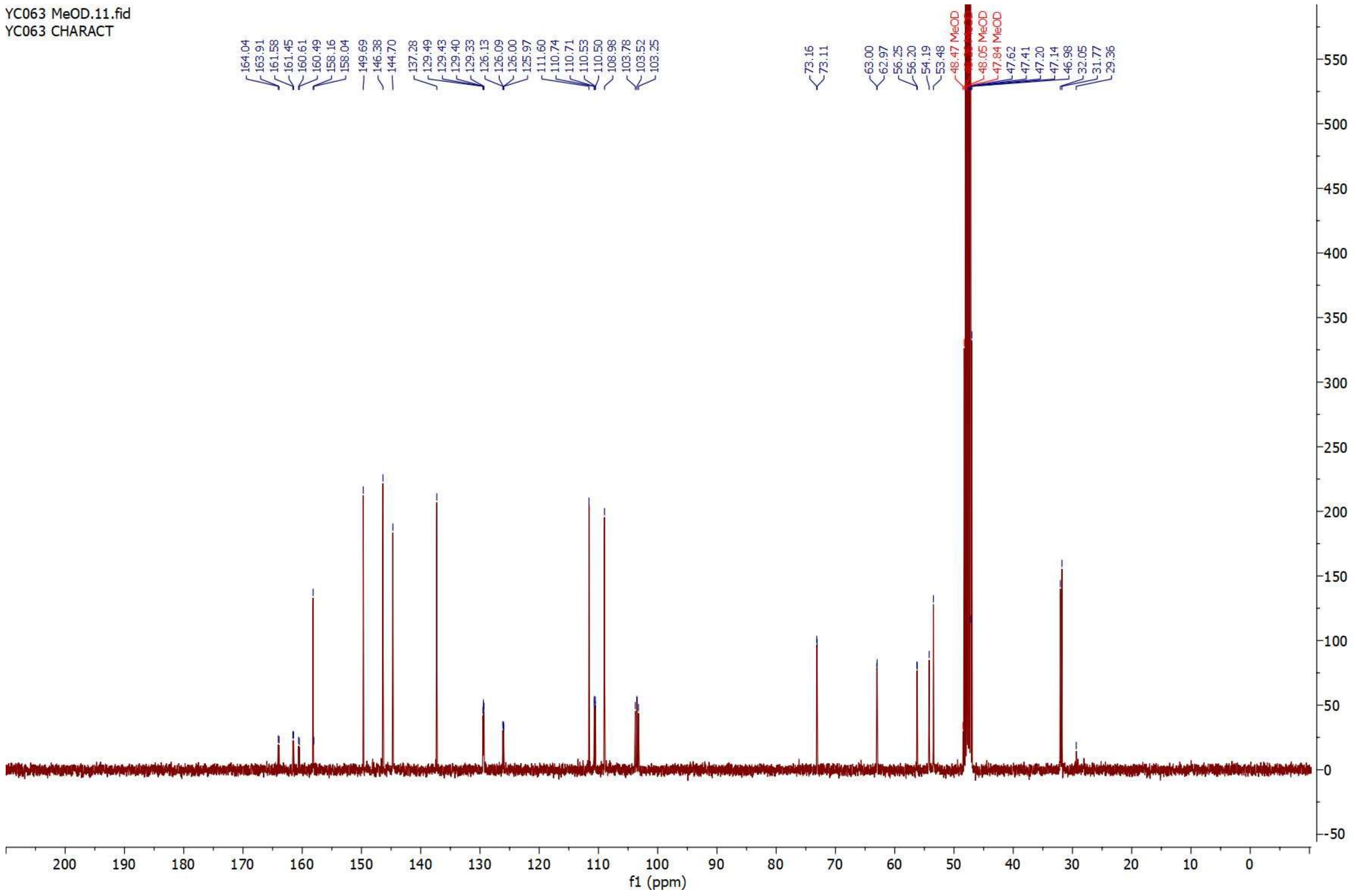

Figure S4-2: Carbon ^{13}C NMR spectrum of Compound **4.**

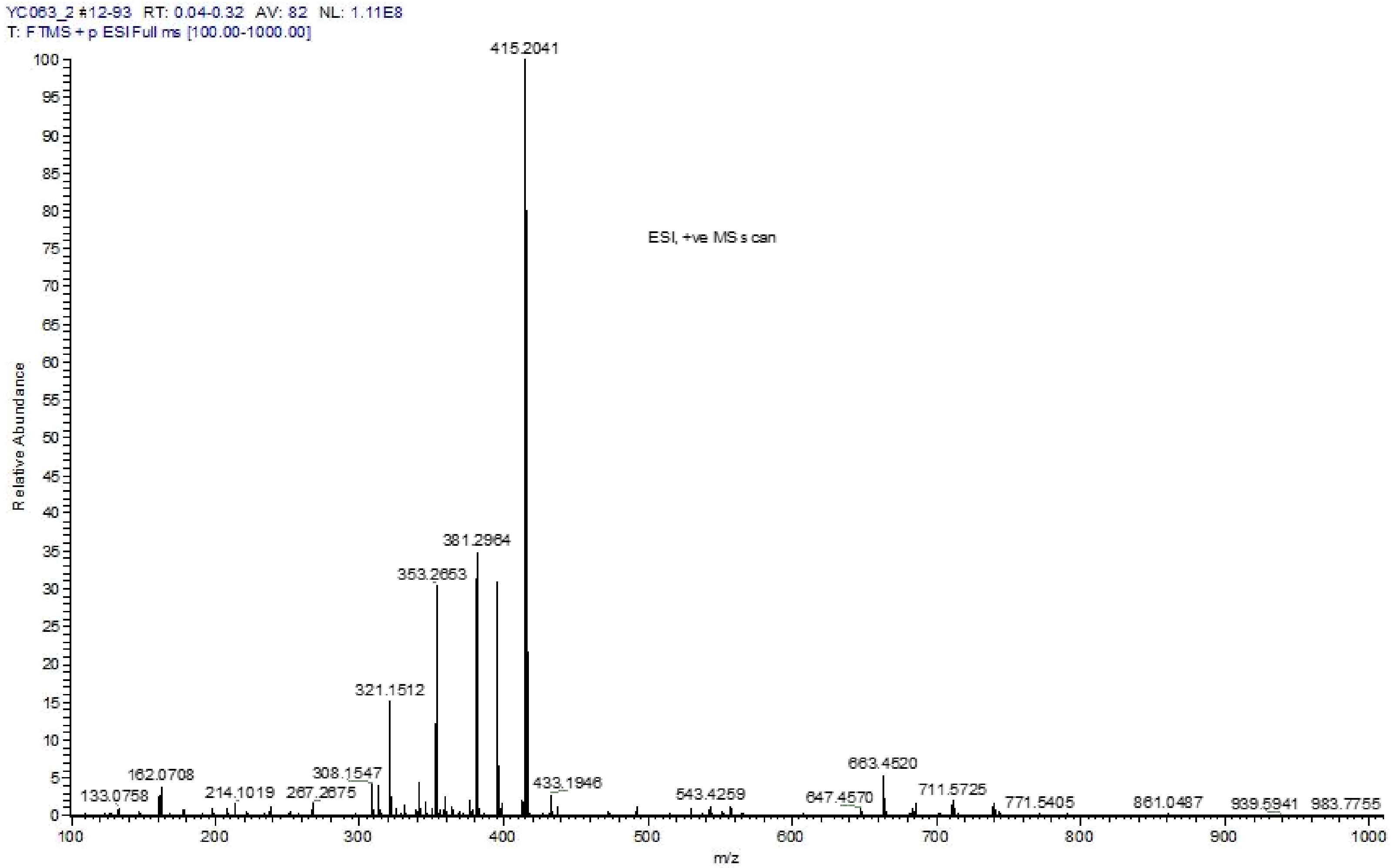

Figure S4-3: HRMS spectrum of Compound **4.**

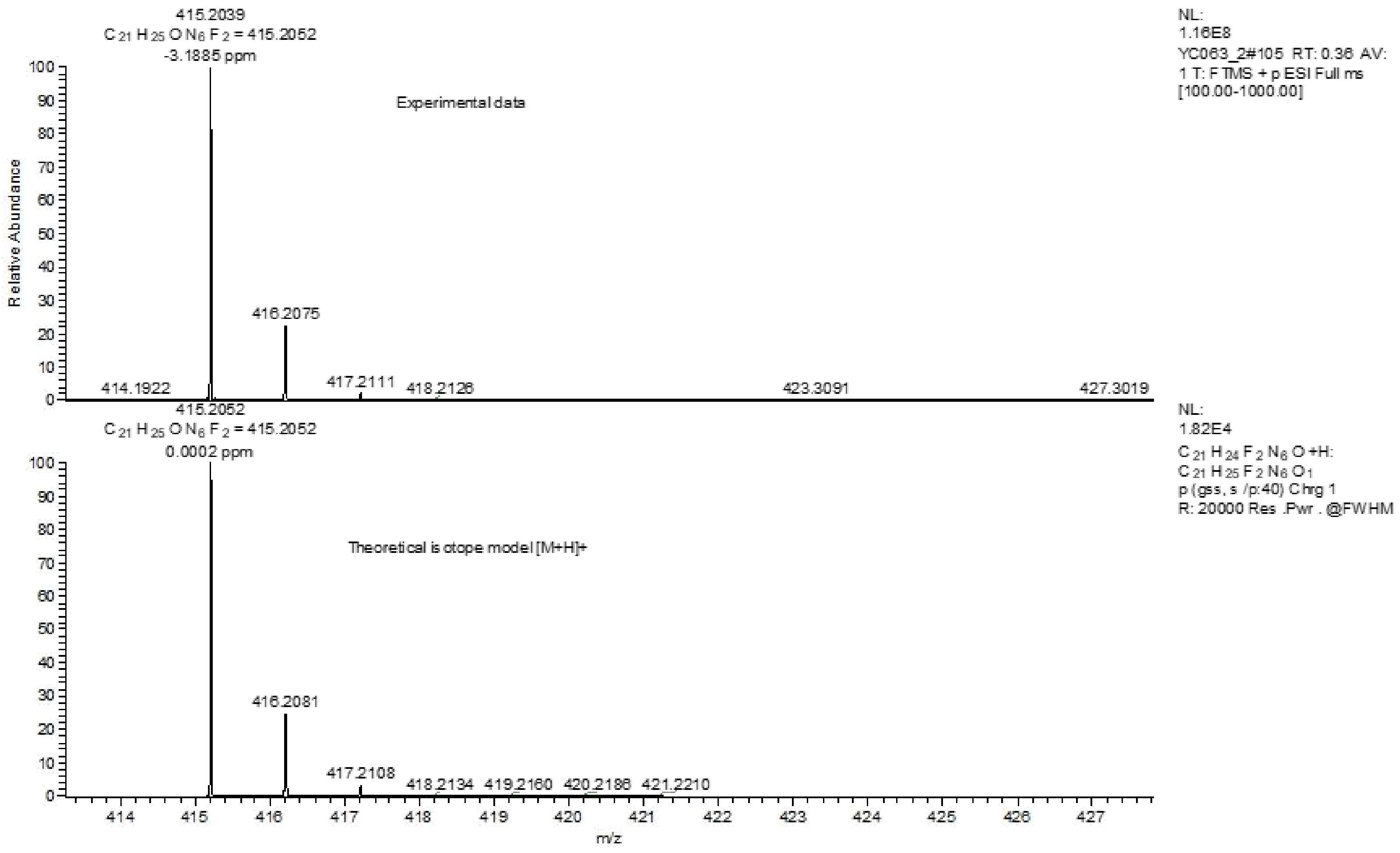

Figure S4-4: HRMS spectrum of Compound **4.**

2-(2,4-difluorophenyl)-1-(4-((5-fluoropyridin-2-yl)amino)piperidin-1-yl)-3-(1H-1,2,4-triazol-1-yl)propan-2-ol (5)

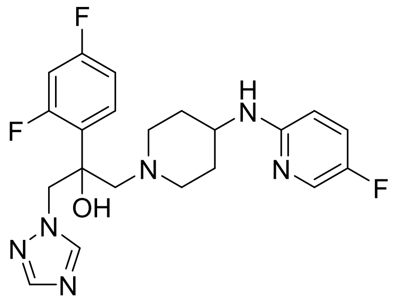

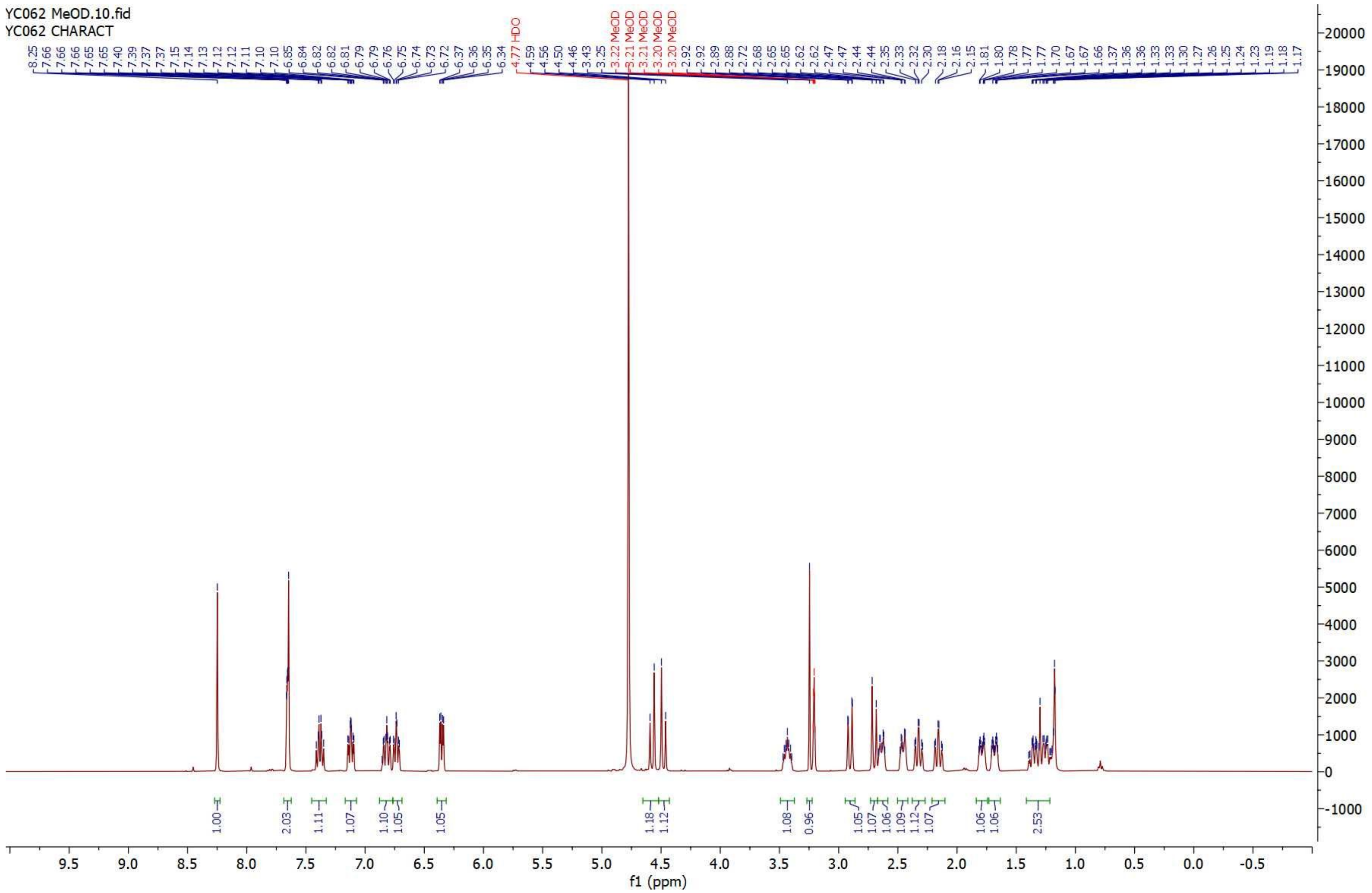

Figure S5-1: Proton ^{1}H NMR spectrum of Compound **5.**

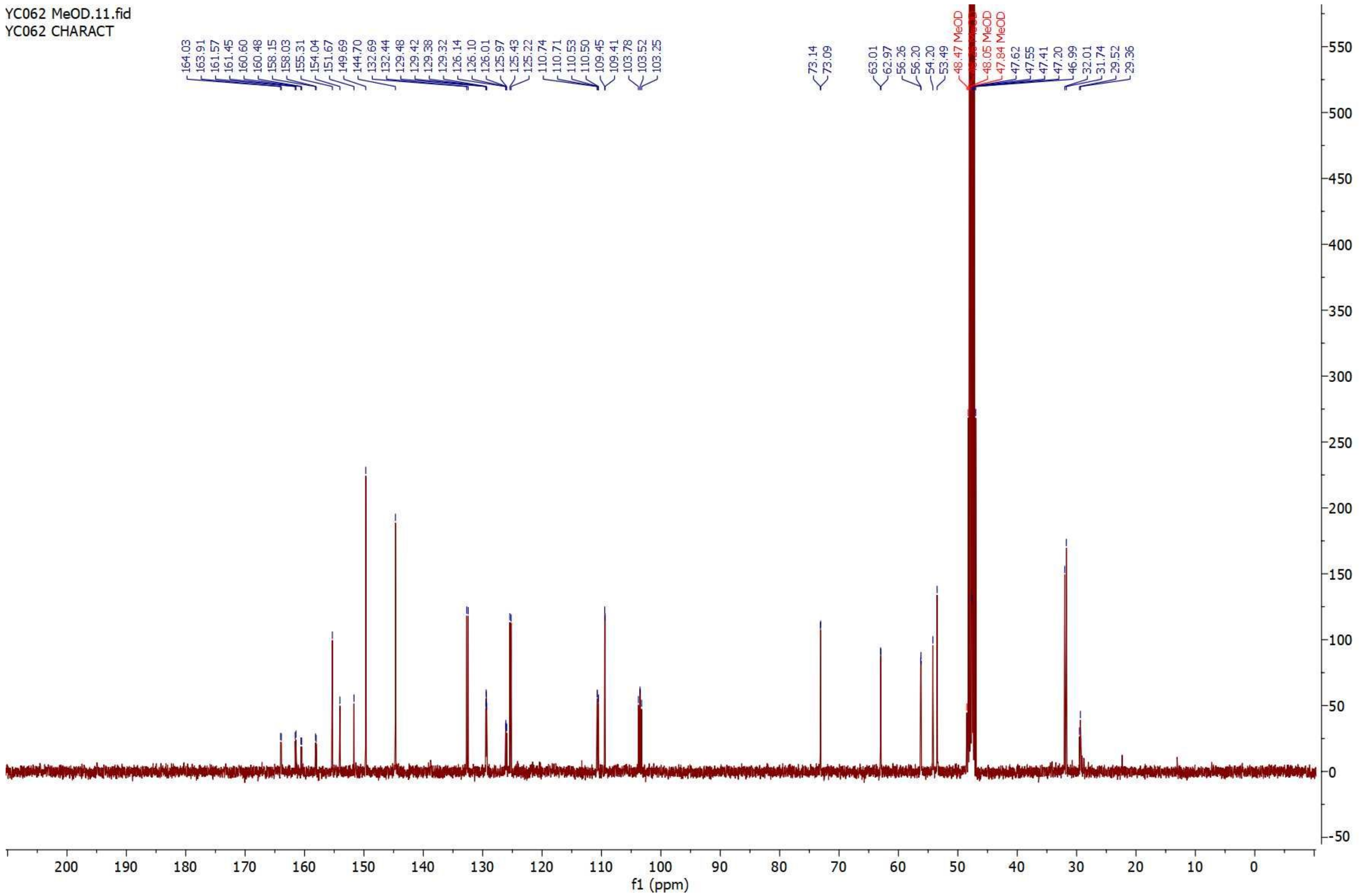

Figure S5-2: Carbon ^{13}C NMR spectrum of Compound **5.**

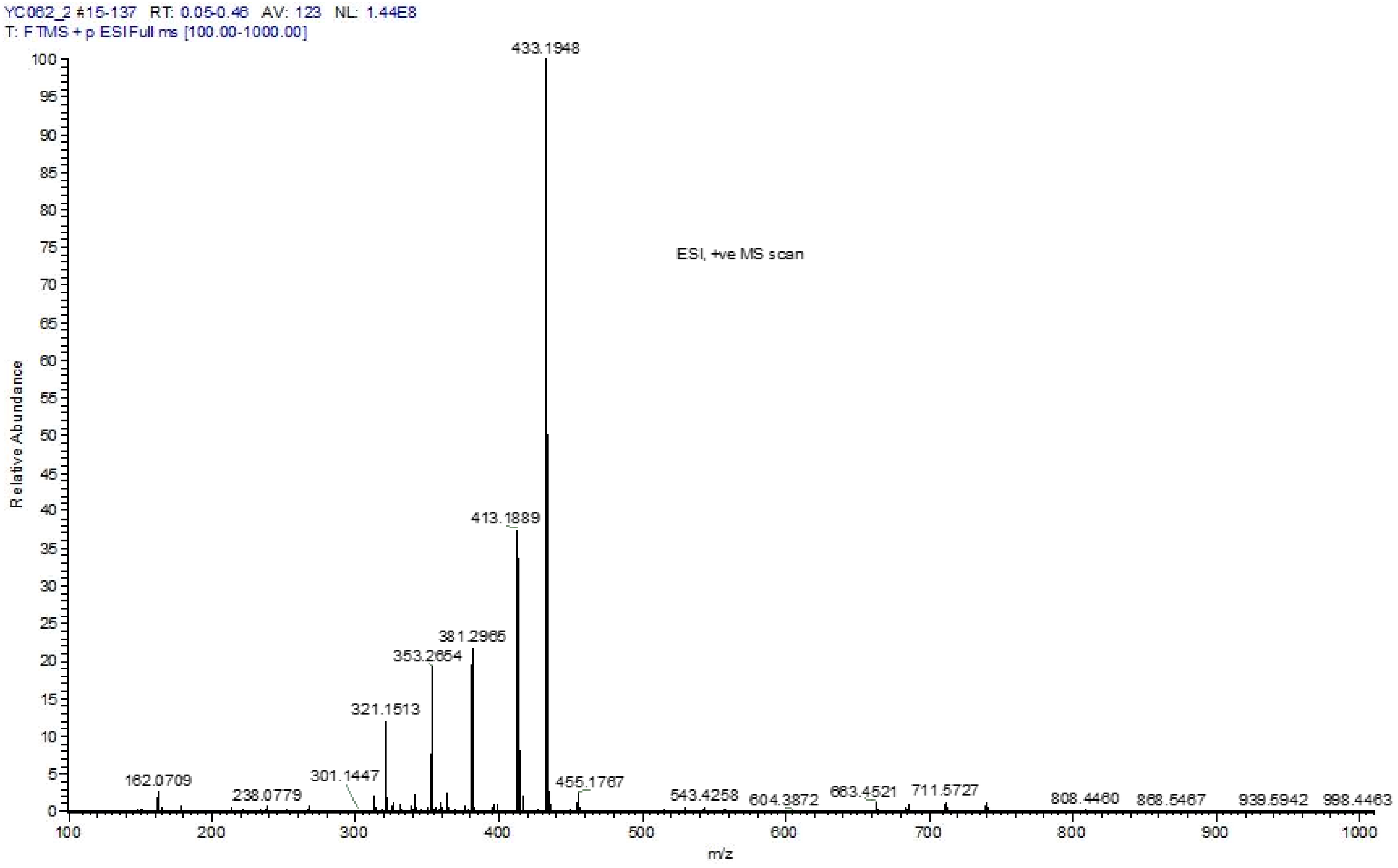

Figure S5-3: HRMS spectrum of Compound **5.**

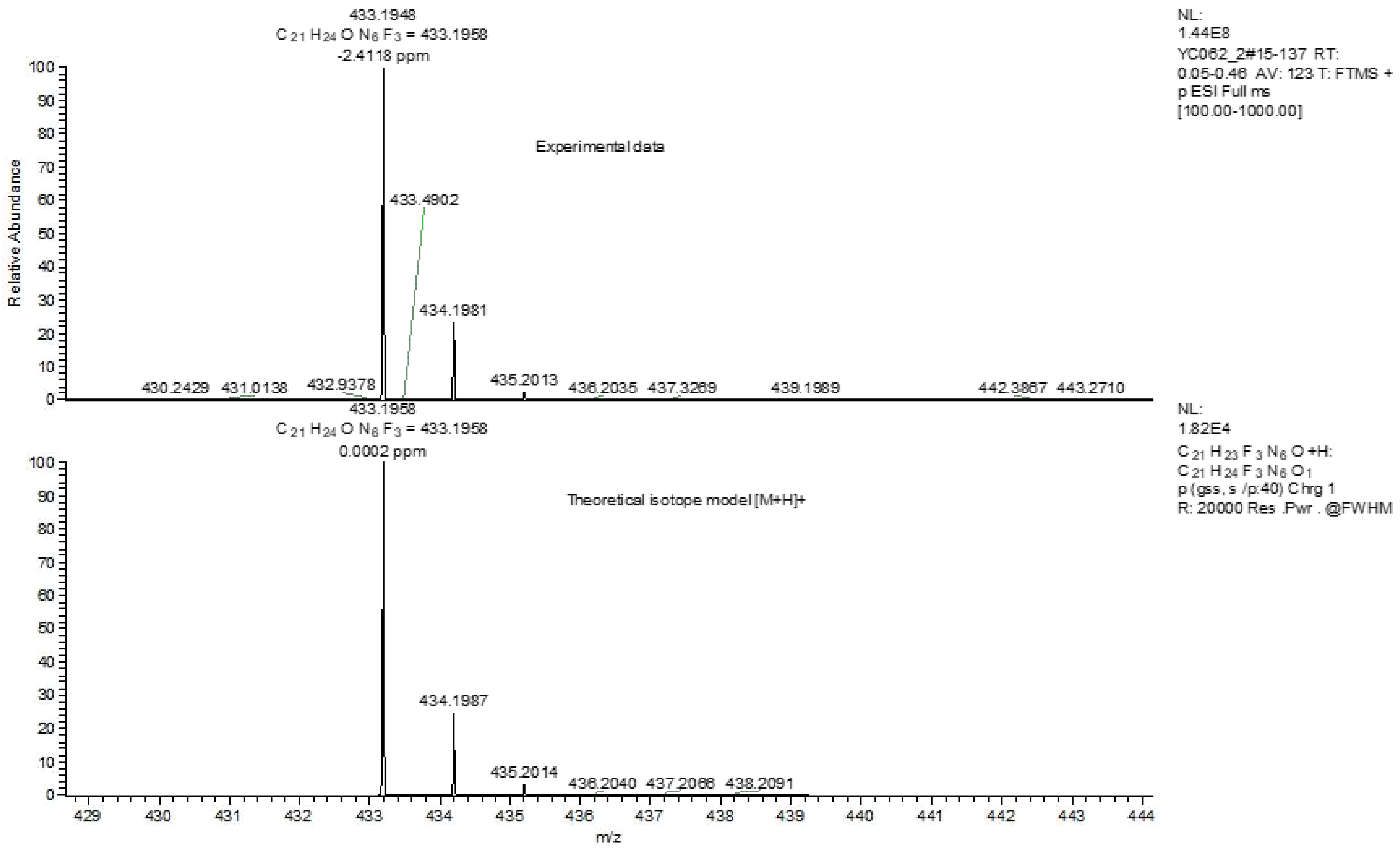

Figure S5-4: HRMS spectrum of Compound **5.**

2-(2,4-difluorophenyl)-1-(4-(pyrimidin-2-ylamino)piperidin-1-yl)-3-(1H-1,2,4-triazol-1-yl)propan-2-ol (6)

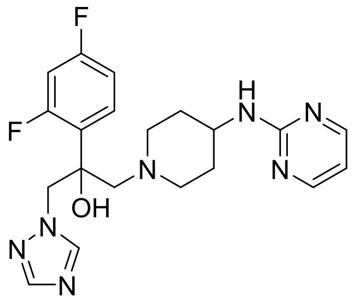

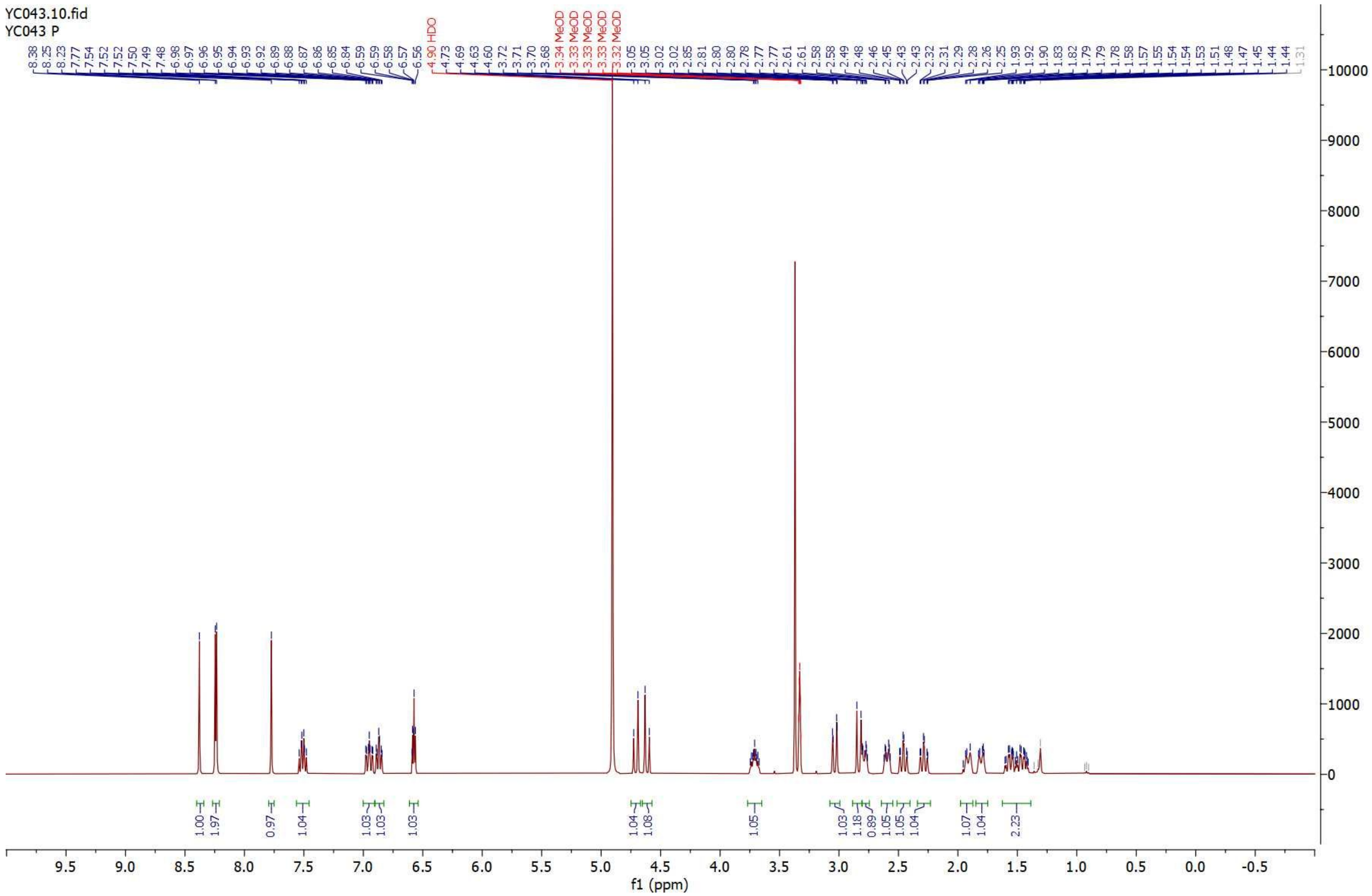

Figure S6-1: Proton ^{1}H NMR spectrum of Compound **6.**

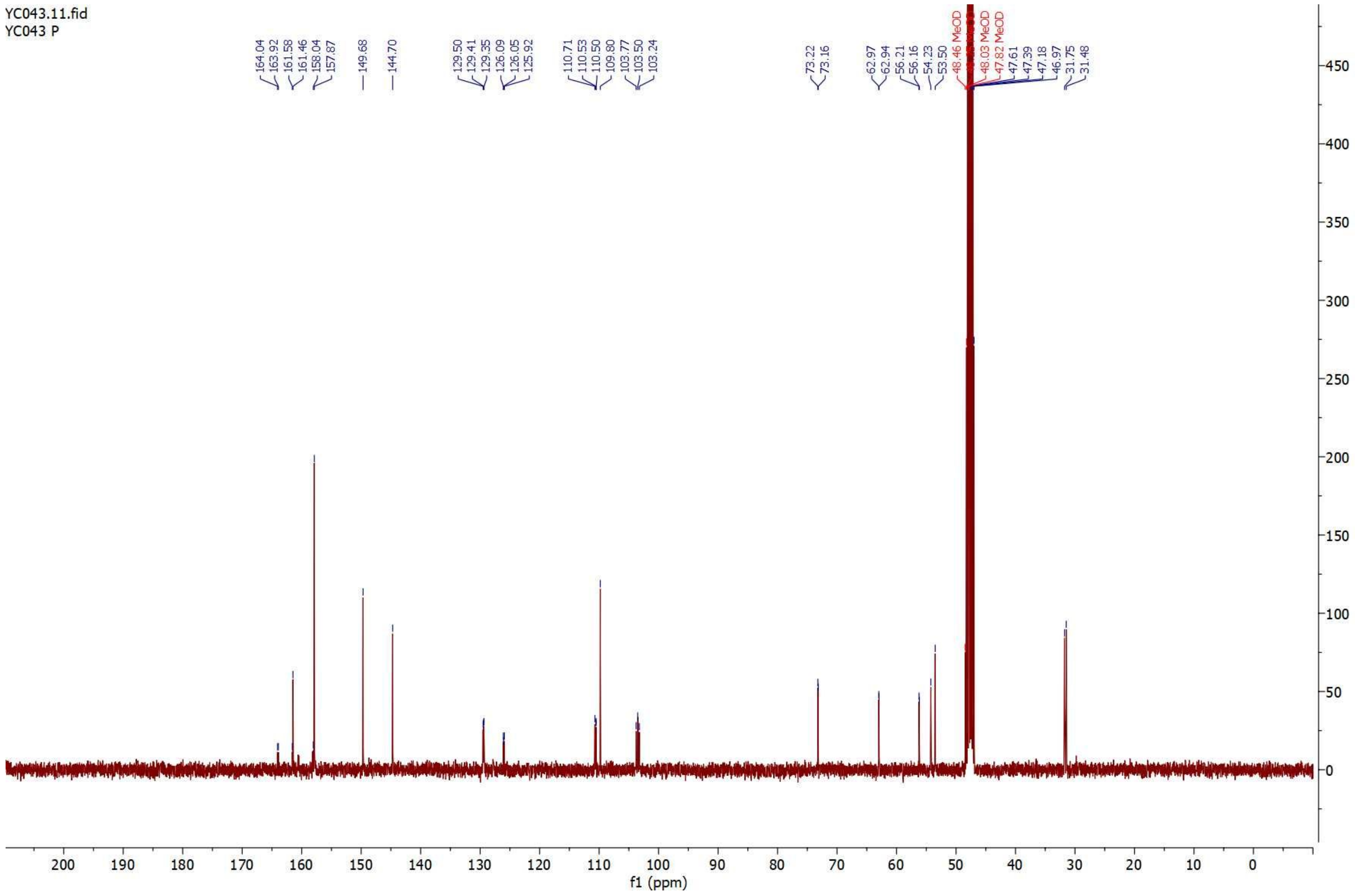

Figure S6-2: Carbon ^{13}C NMR spectrum of Compound **6.**

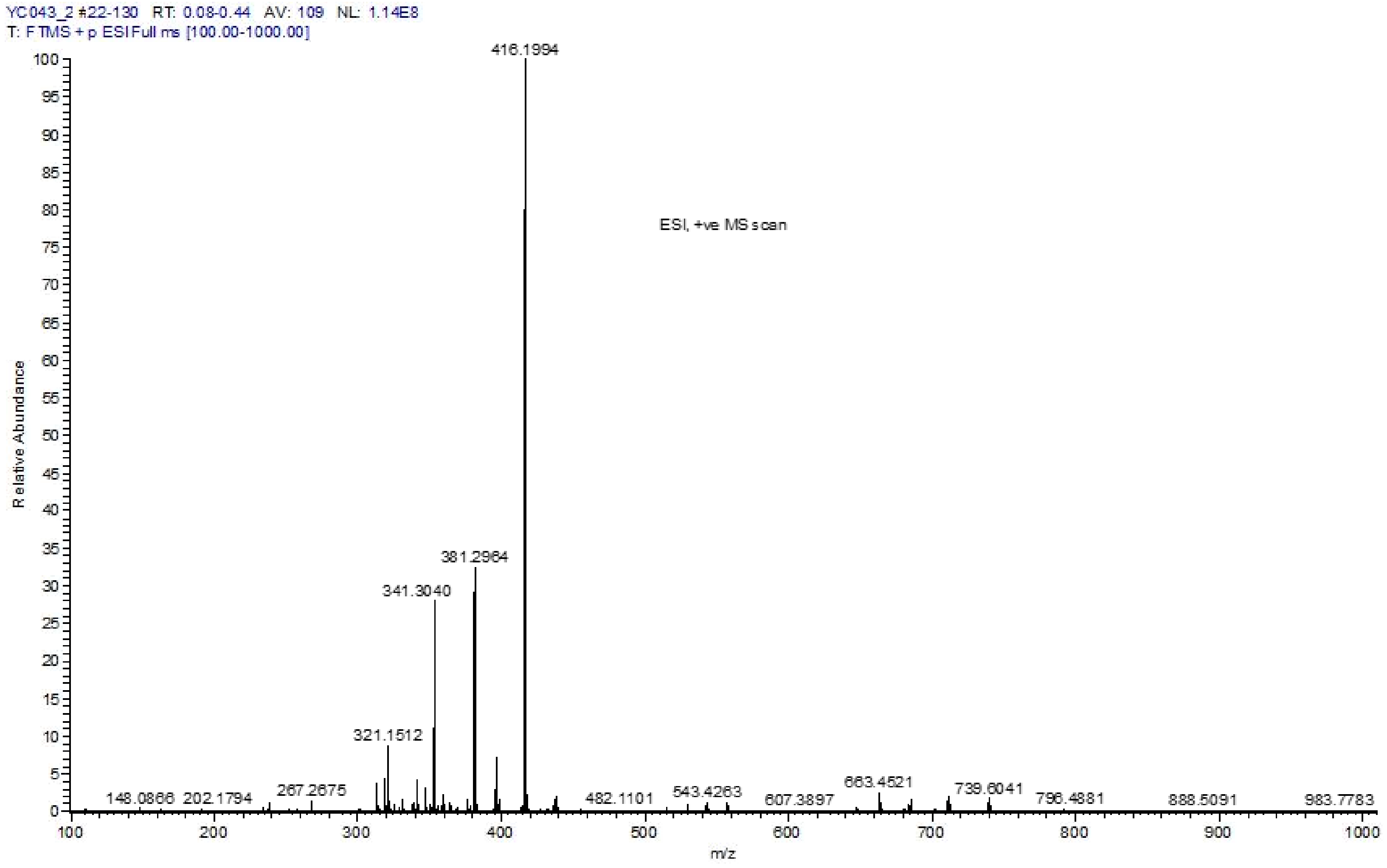

Figure S6-3: HRMS spectrum of Compound **6.**

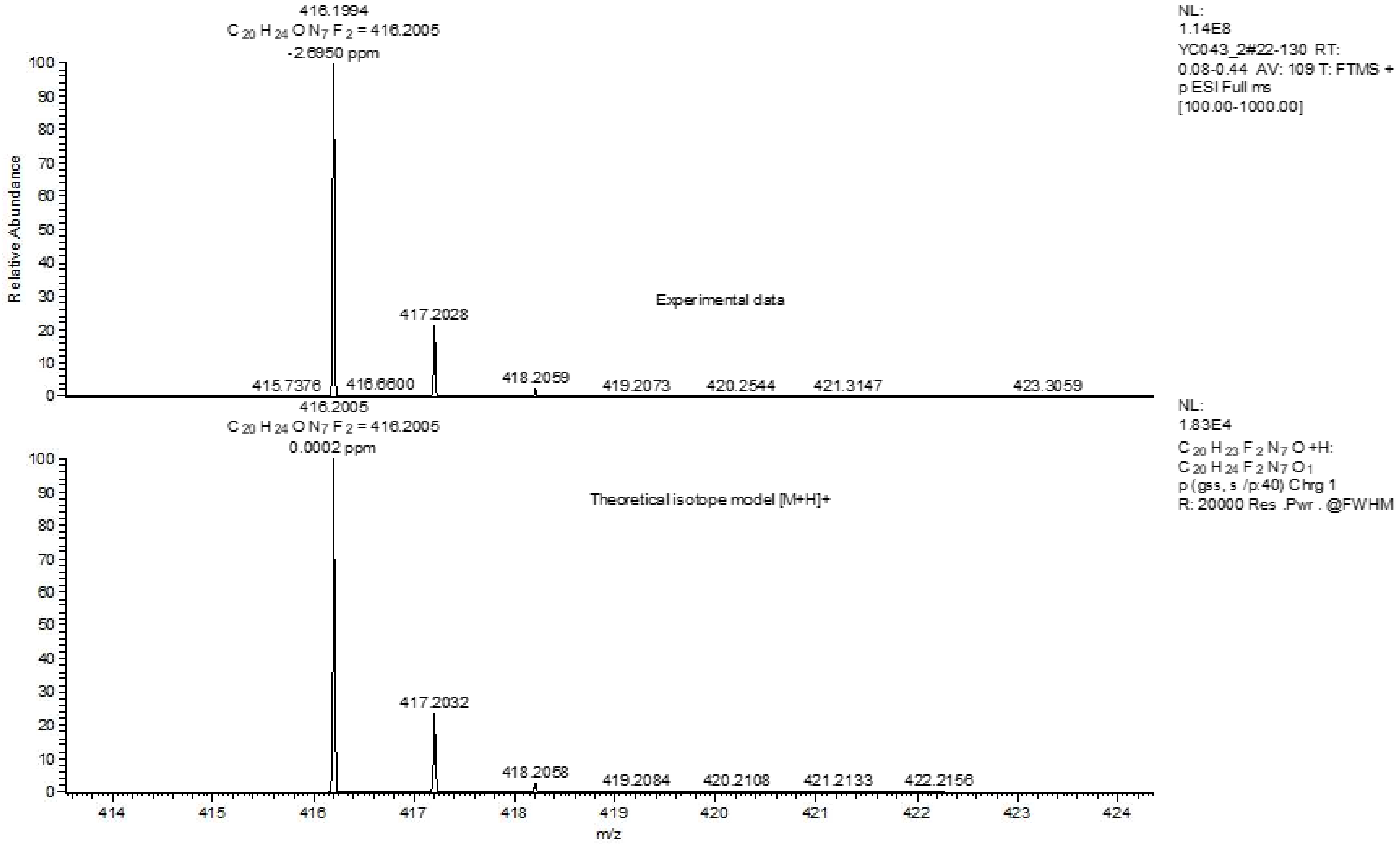

Figure S6-4: HRMS spectrum of Compound **6.**

2-(2,4-difluorophenyl)-1-(4-((5-fluoropyrimidin-2-yl)amino)piperidin-1-yl)-3-(1H-1,2,4-triazol-1-yl)propan-2-ol (7)

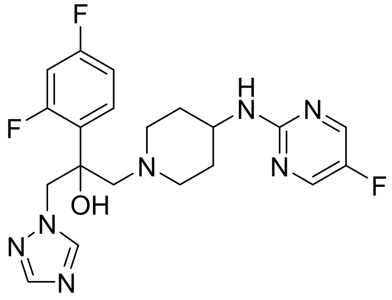

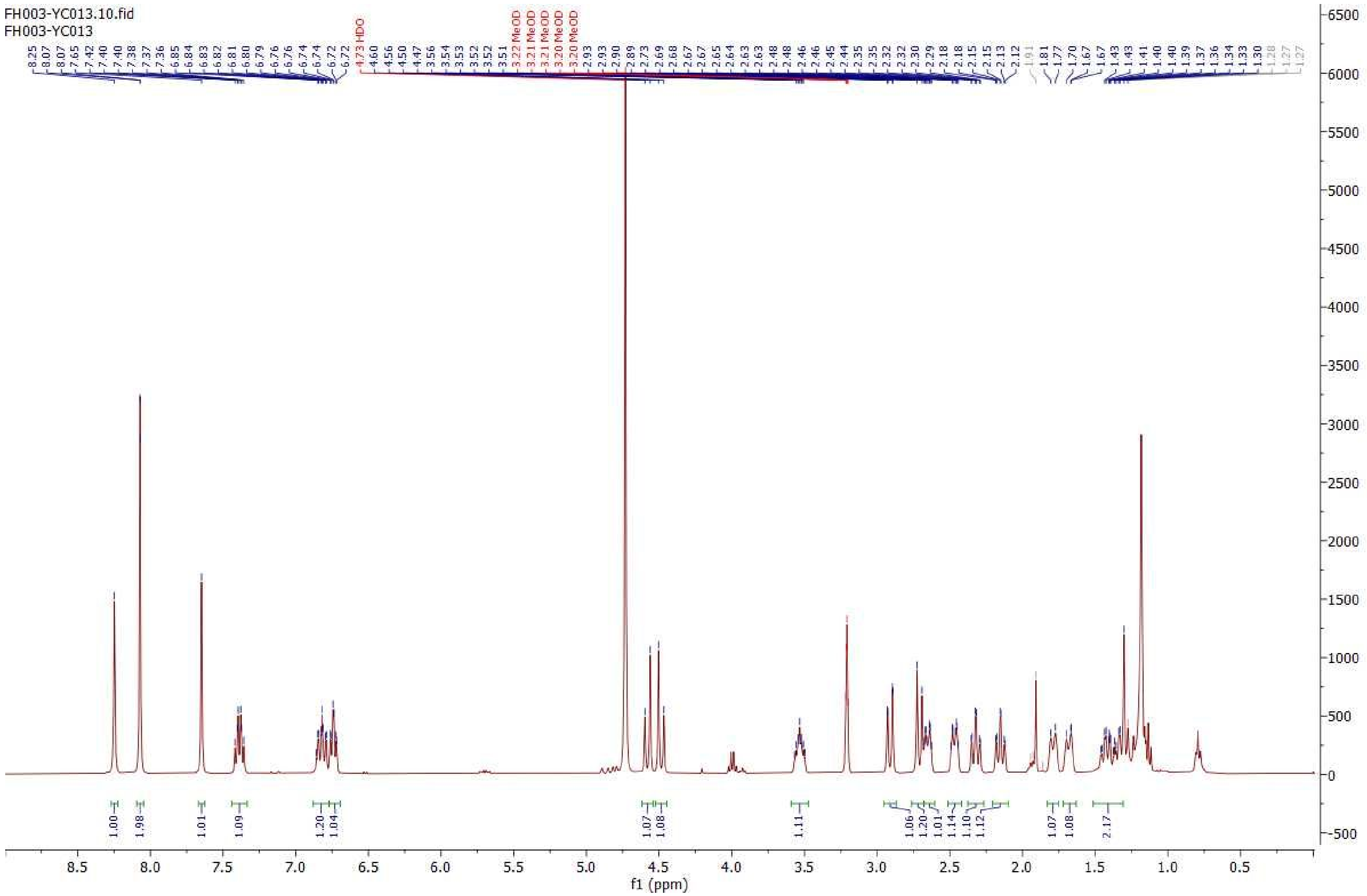
FH003-YC013.10.fid
FH003-YC013
f1 (ppm)

Figure S7-1: Proton ^{1}H NMR spectrum of Compound **7.**

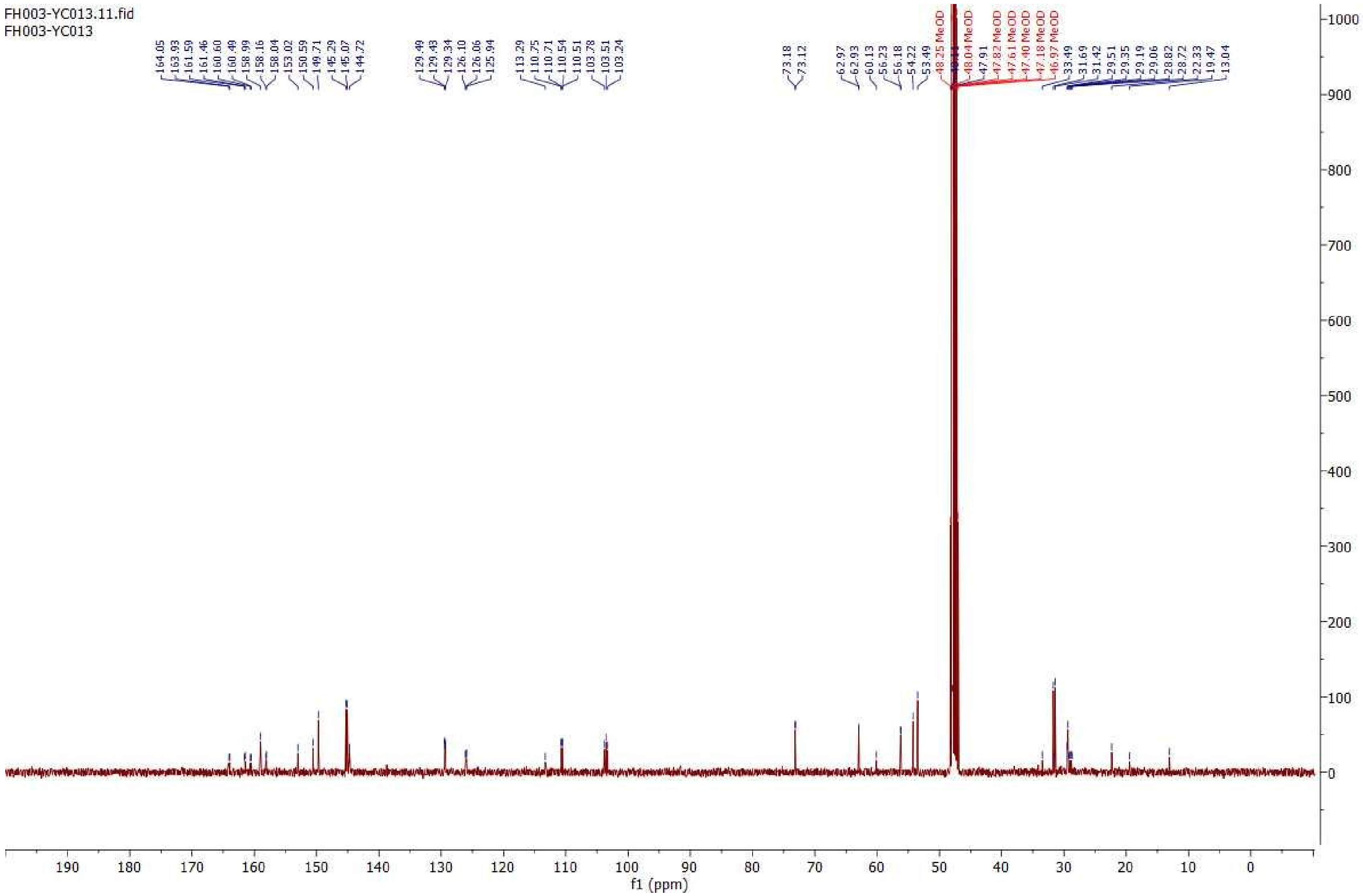

FH003-YC013.11.fid
FH003-YC013
f1 (ppm)

Figure S7-2: Carbon ^{13}C NMR spectrum of Compound **7.**

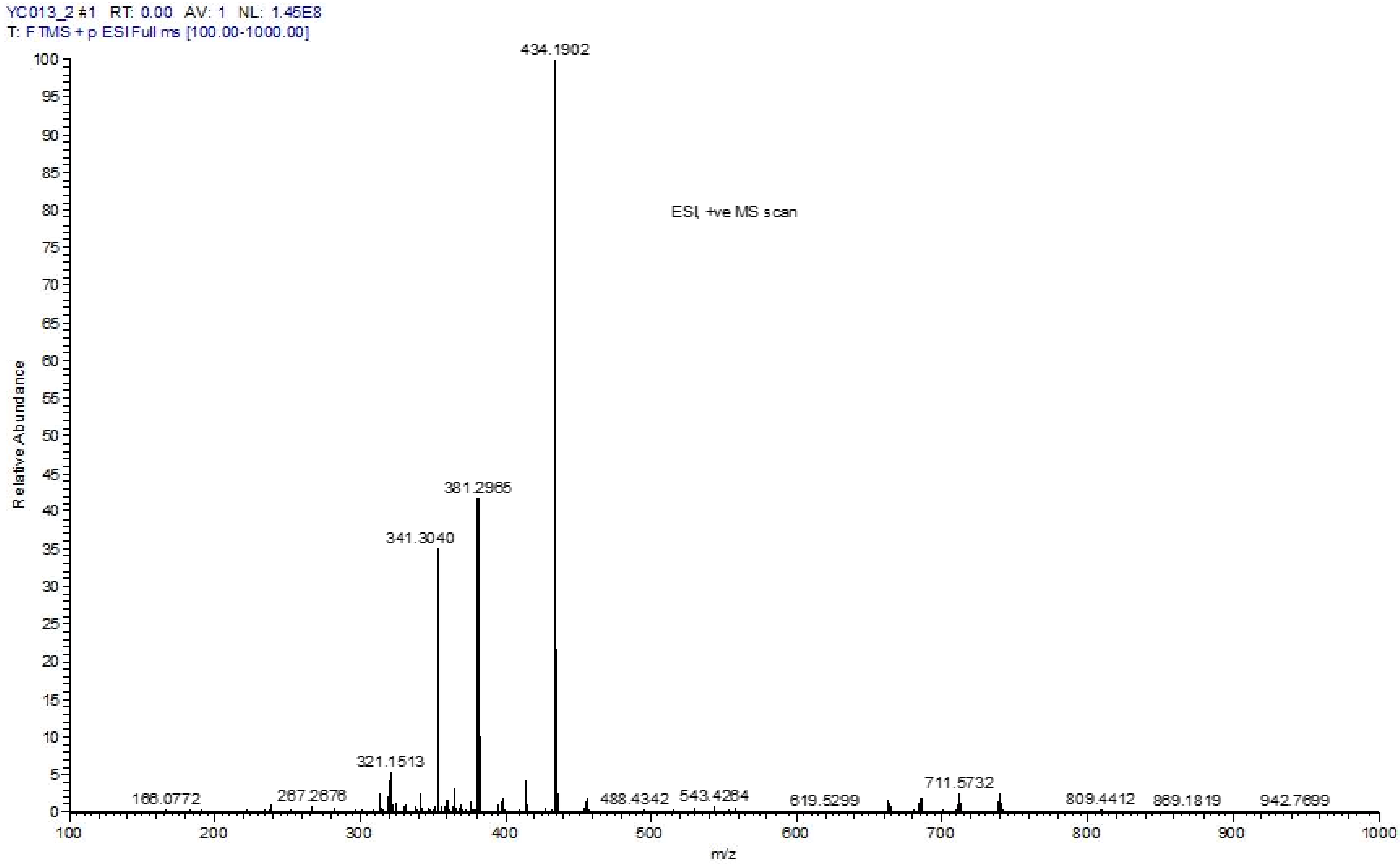
YC013_2 #1 RT: 0.00 AV: 1 NL: 1.45E8
T: FTMS + p ESI Full ms [100.00-1000.00]
434.1902
381.2965
341.3040
321.1513
166.0772
267.2676
488.4342
543.4264
619.5299
711.5732
809.4412
869.1819
942.7699
ESI, +ve MS scan
Relative Abundance
m/z

Figure S7-3: HRMS spectrum of Compound **7.**

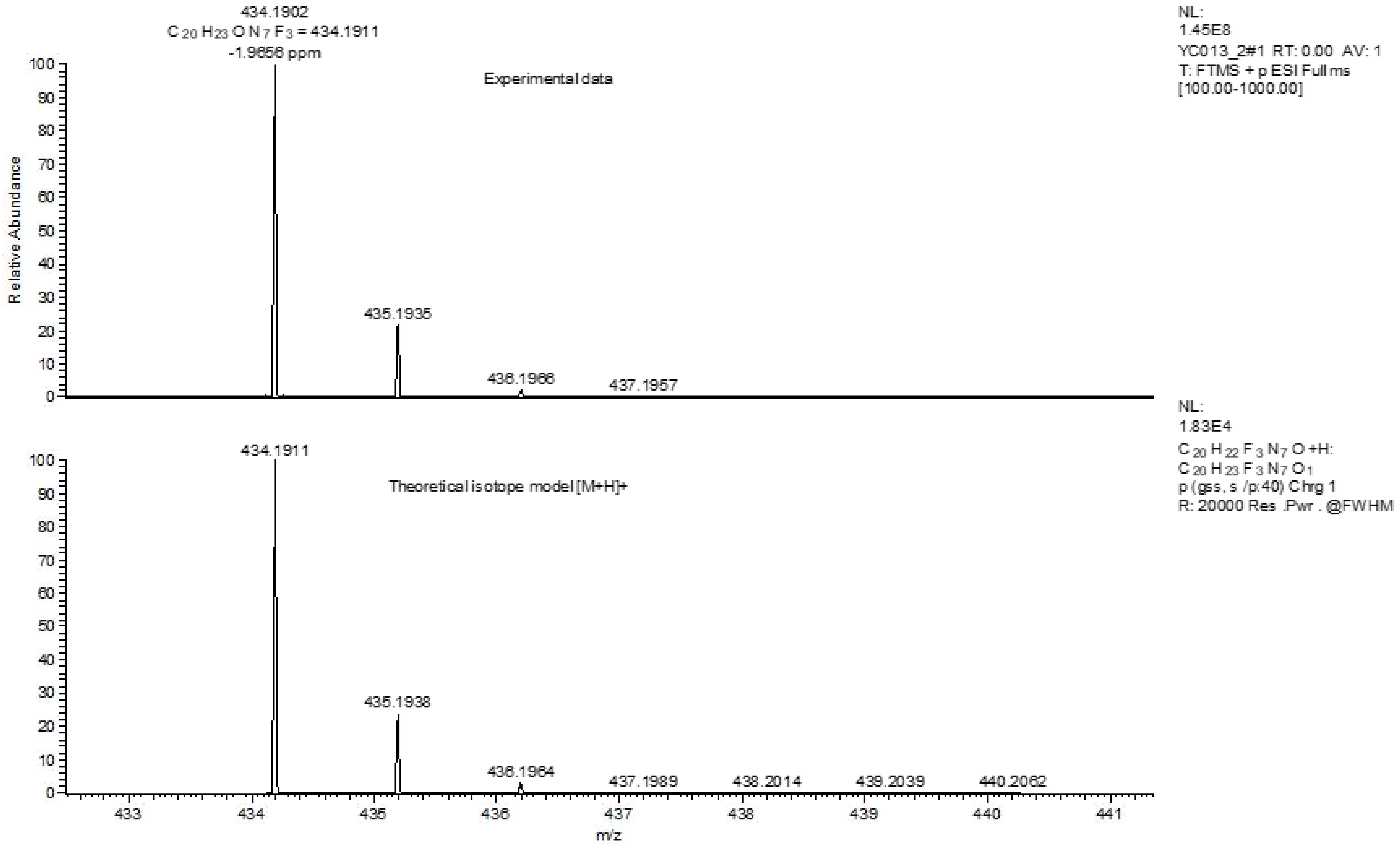
434.1902
C 20 H23 O N 7 F 3 = 434.1911
-1.9656 ppm
Experimental data
435.1935
436.1966
437.1957
NL:
1.45E8
YC013_2#1 RT: 0.00 AV: 1
T: FTMS + p ESI Full ms
[100.00-1000.00]
Relative Abundance
434.1911
Theoretical isotope model [M+H]+
435.1938
436.1964
437.1989
438.2014
439.2039
440.2062
NL:
1.83E4
C 20 H 22 F 3 N 7 O +H:
C 20 H 23 F 3 N 7 O 1
p (gss, s /p:40) Chrg 1
R: 20000 Res .Pwr . @FWHM
m/z

Figure S7-4: HRMS spectrum of Compound **7.**

2-(2,4-Difluorophenyl)-1-(4-((5-methylpyrimidin-2-yl)amino)piperidin-1-yl)-3-(1H-1,2,4-triazol-1-yl)propan-2-ol (8)

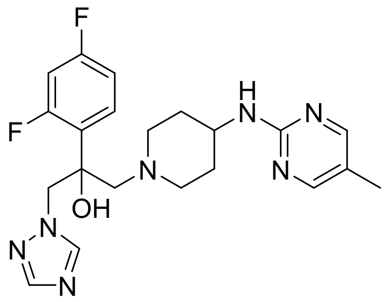

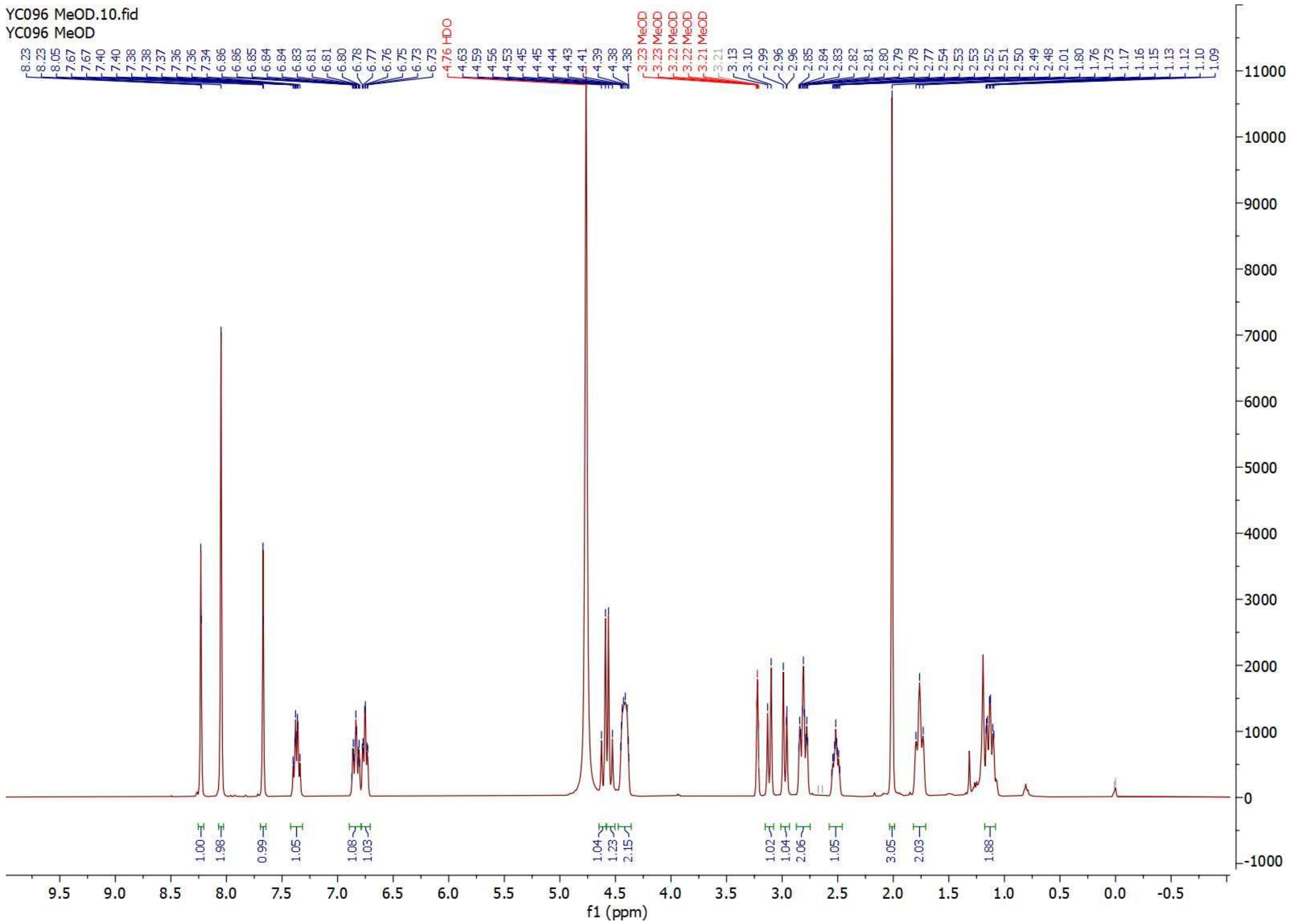

Figure S8-1: Proton ^{1}H NMR spectrum of Compound **8.**

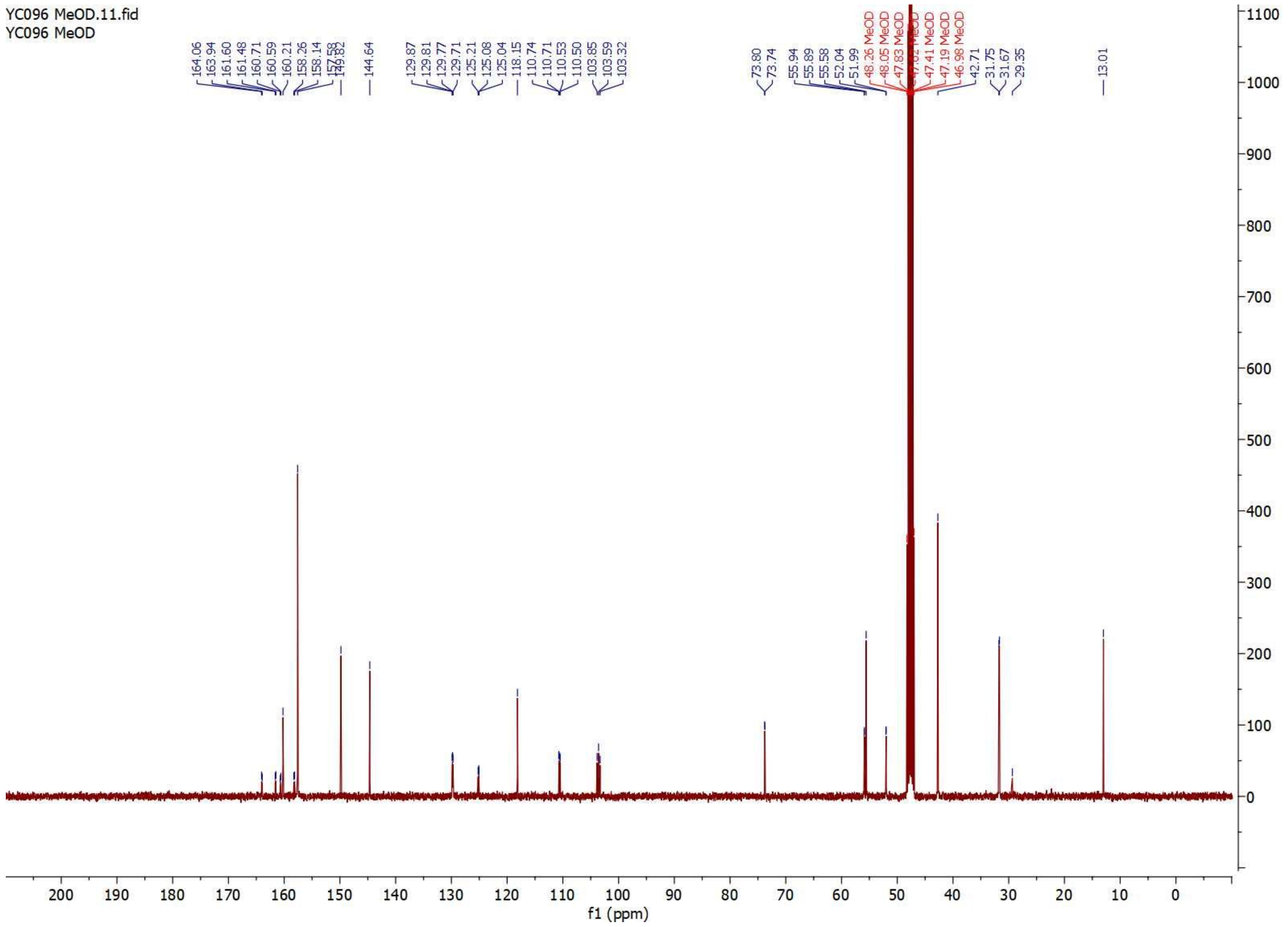

Figure S8-2: Carbon ^{13}C NMR spectrum of Compound **8.**

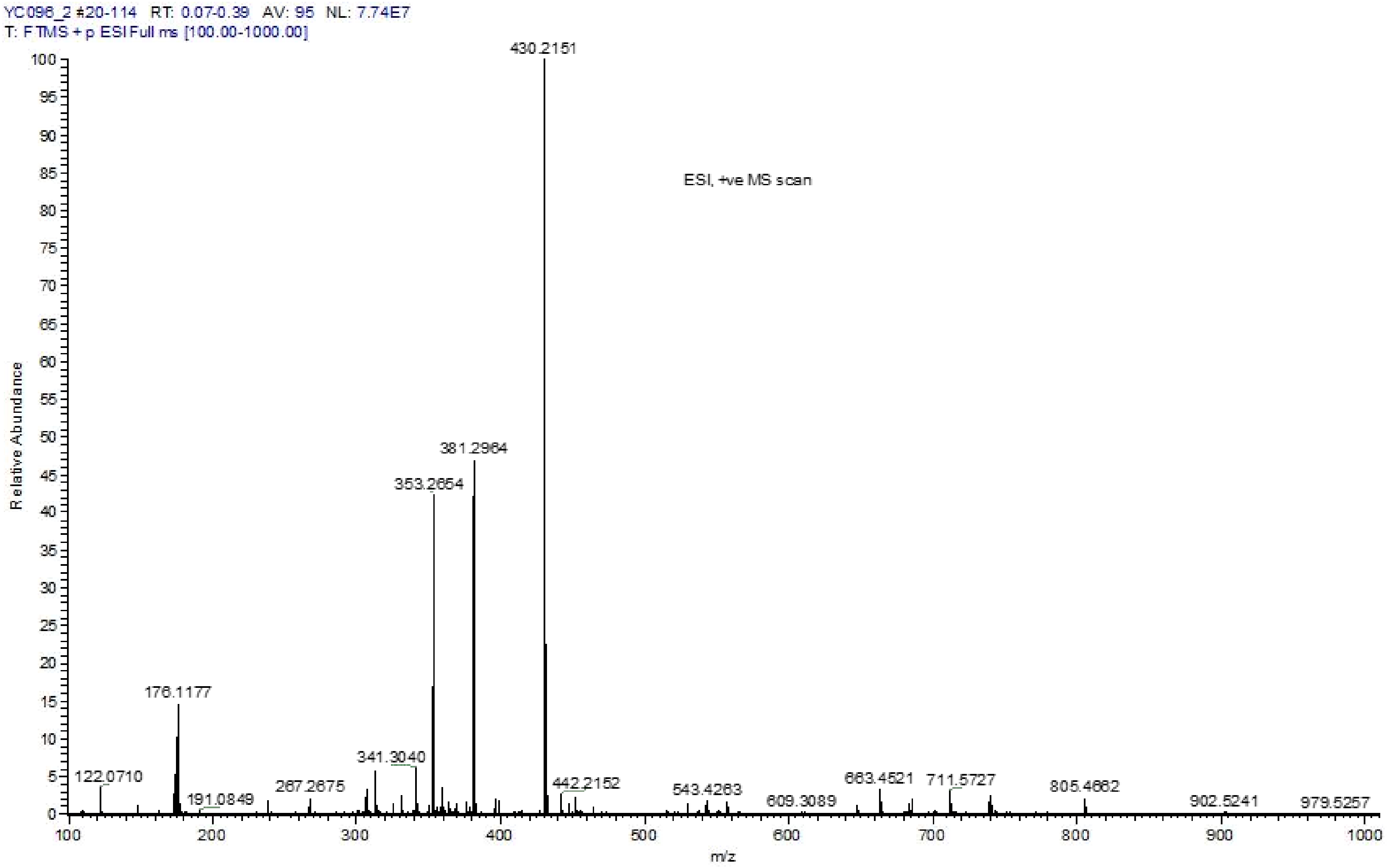

Figure S8-3: HRMS spectrum of Compound **8.**

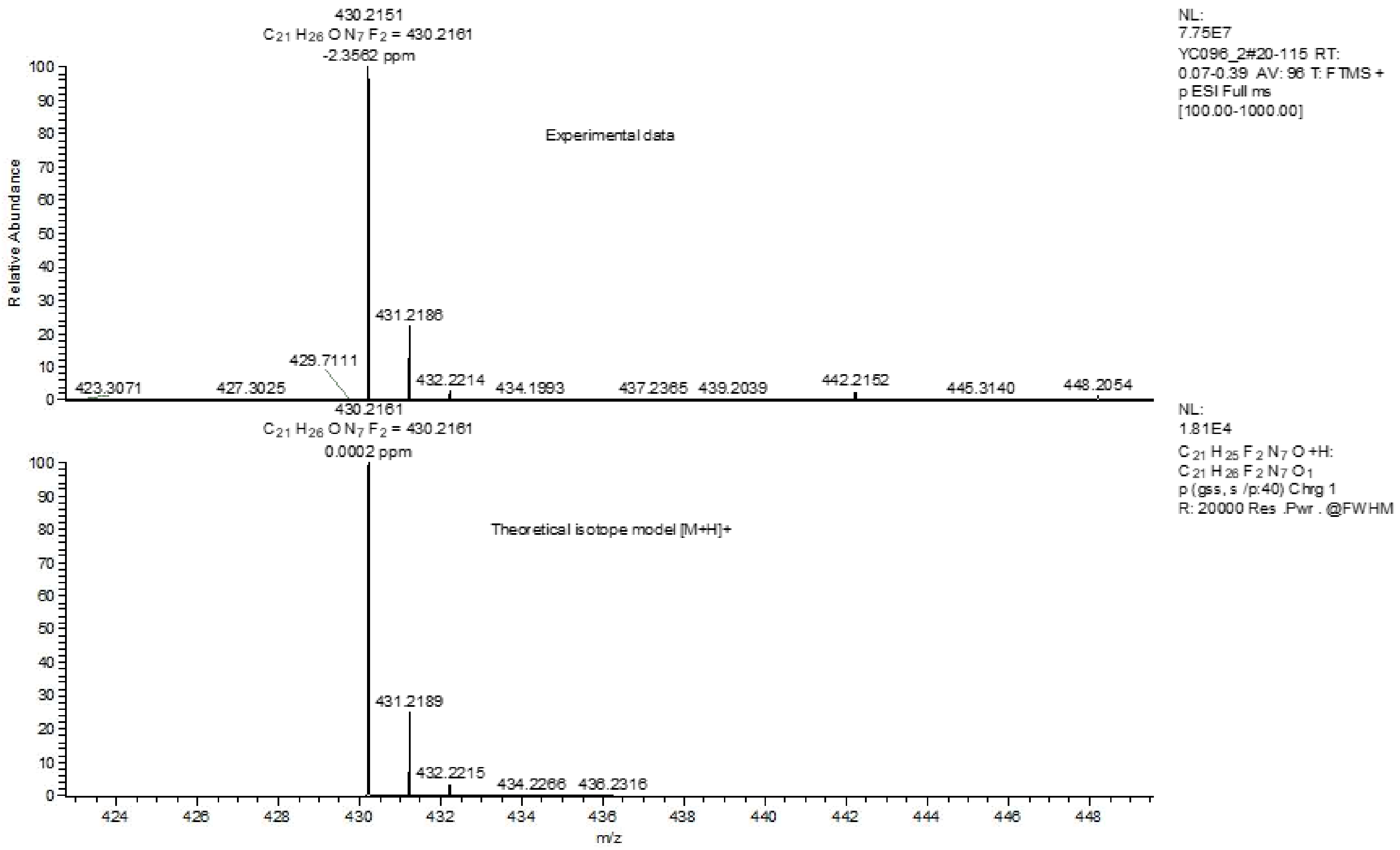

Figure S8-4: HRMS spectrum of Compound **8.**

2-(2,4-Difluorophenyl)-1-(4-((4,6-dimethylpyrimidin-2-yl)amino)piperidin-1-yl)-3-(1H-1,2,4-triazol-1-yl)propan-2-ol (9)

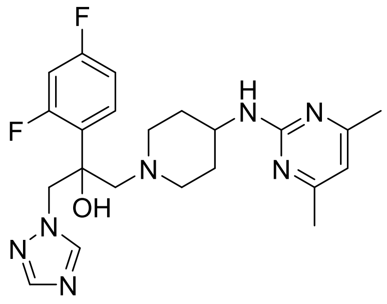

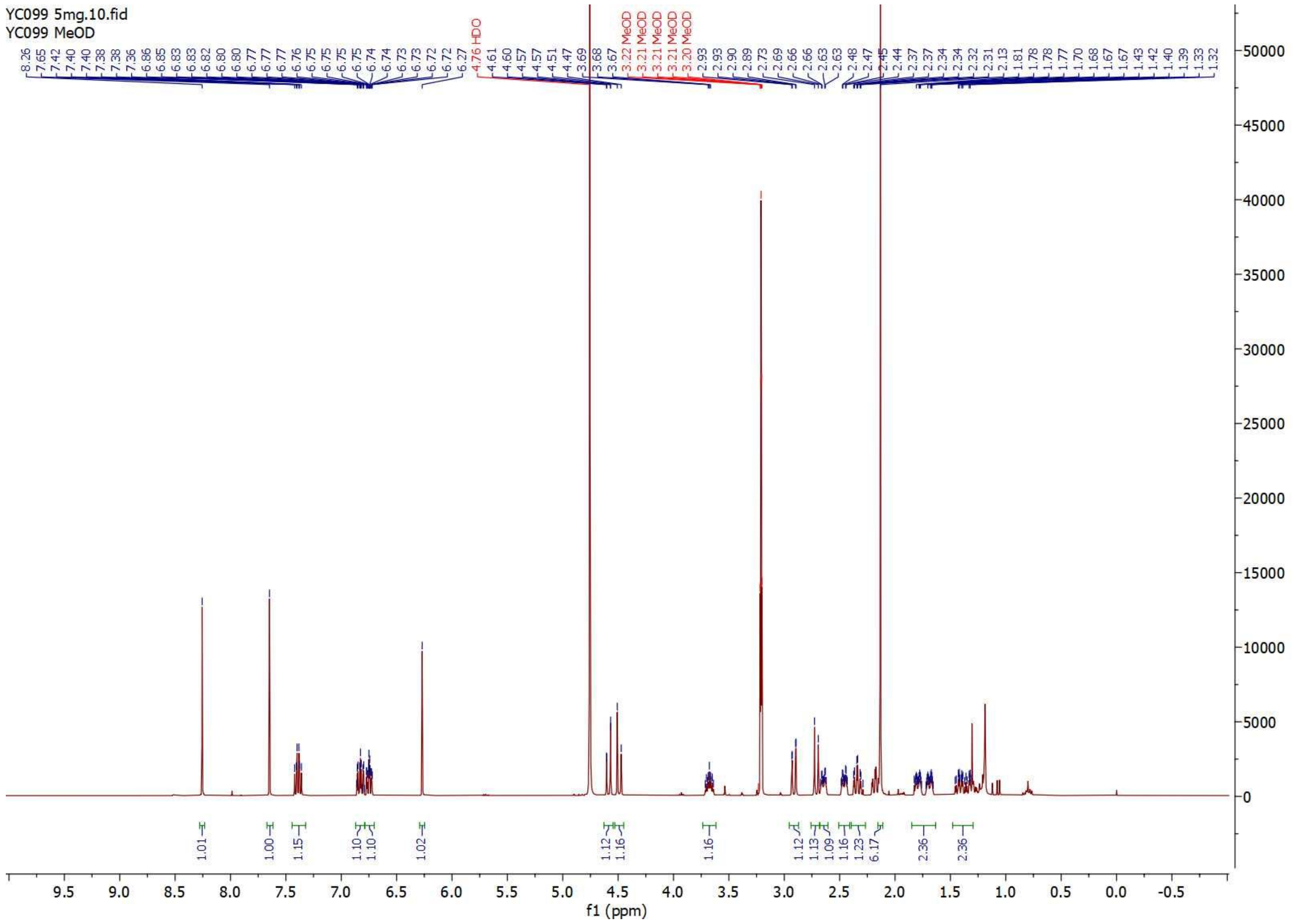

Figure S9-1: Proton ^{1}H NMR spectrum of Compound **9.**

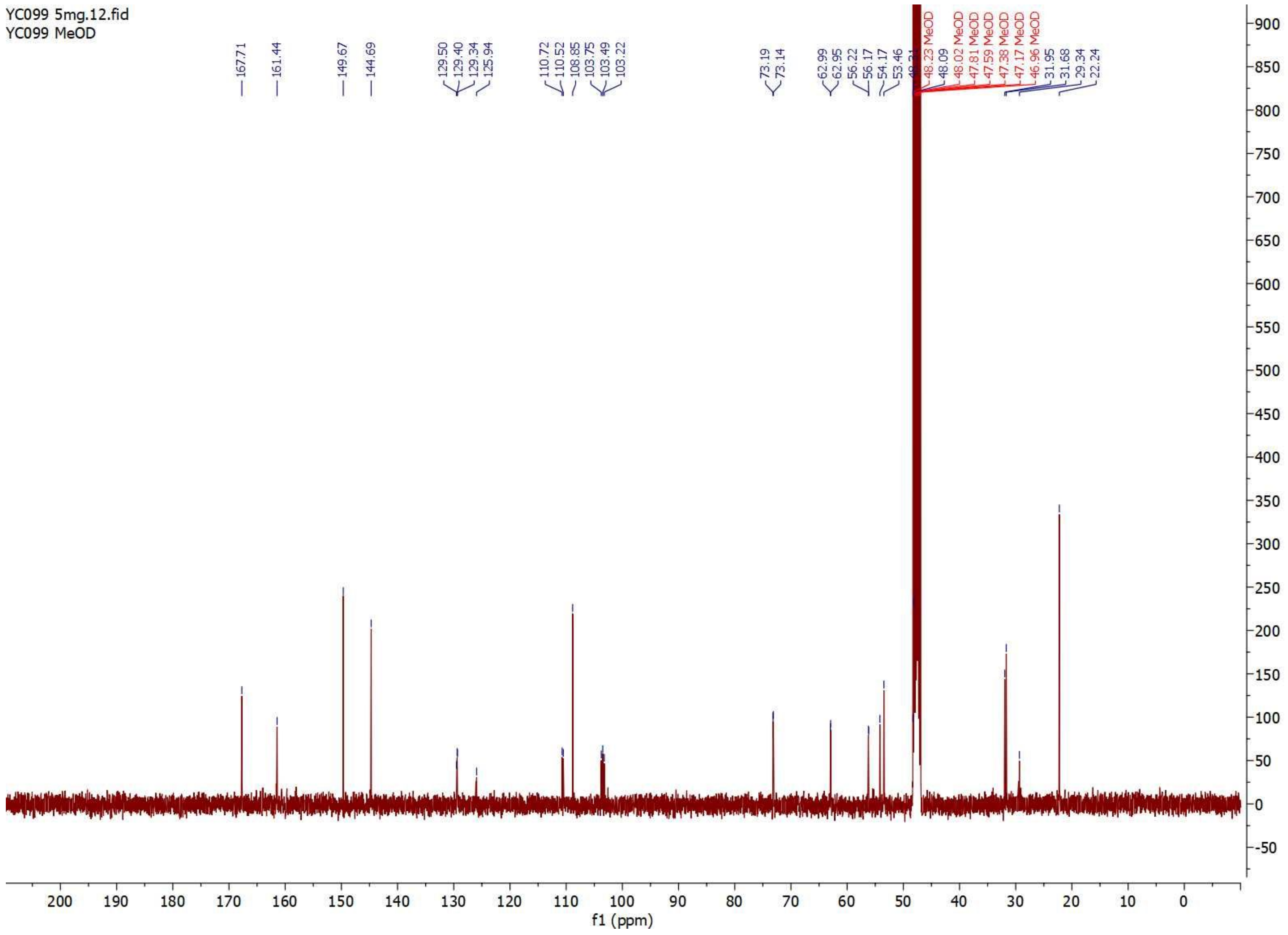

Figure S9-2: Carbon ^{13}C NMR spectrum of Compound **9.**

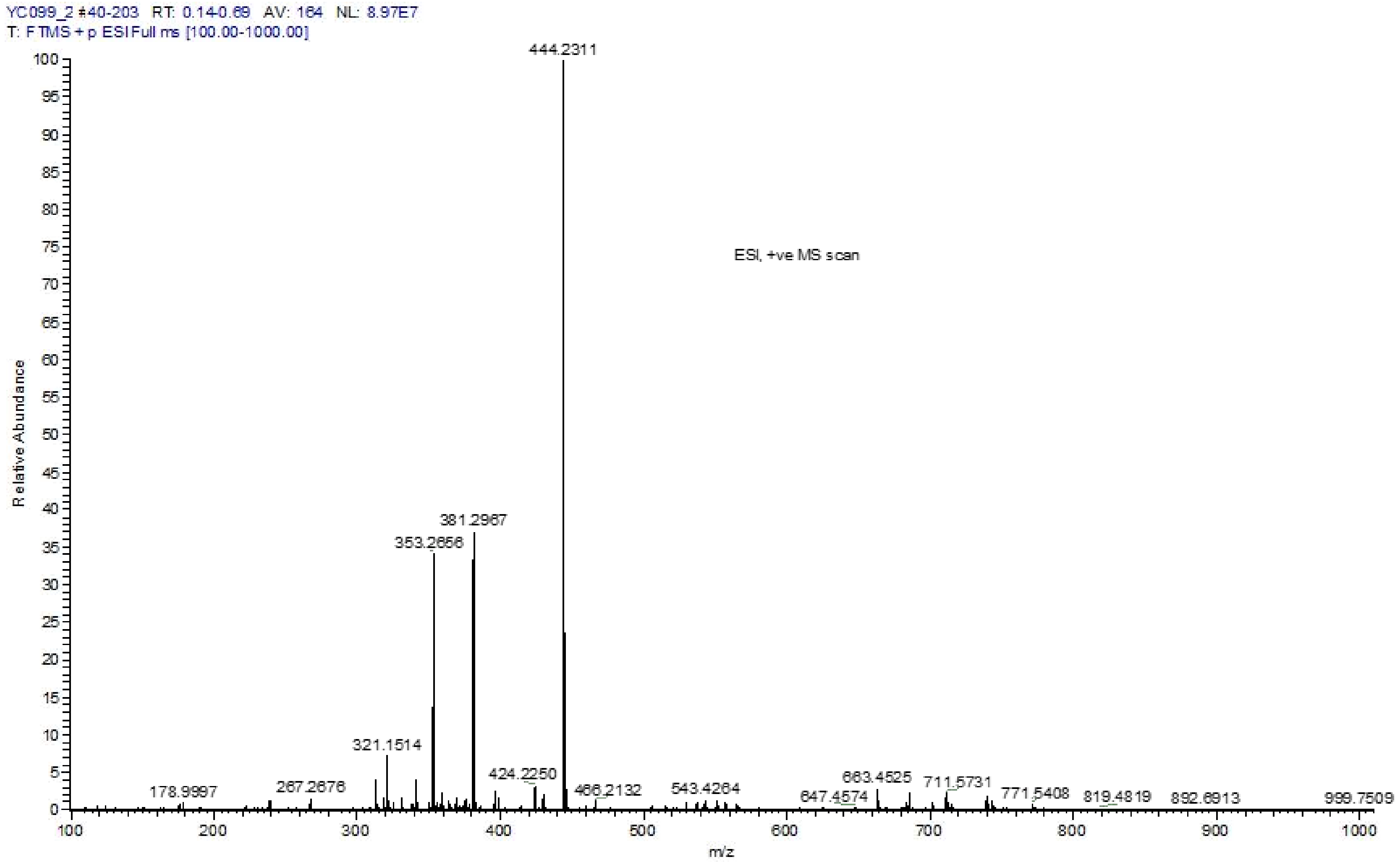

Figure S9-3: HRMS spectrum of Compound **9.**

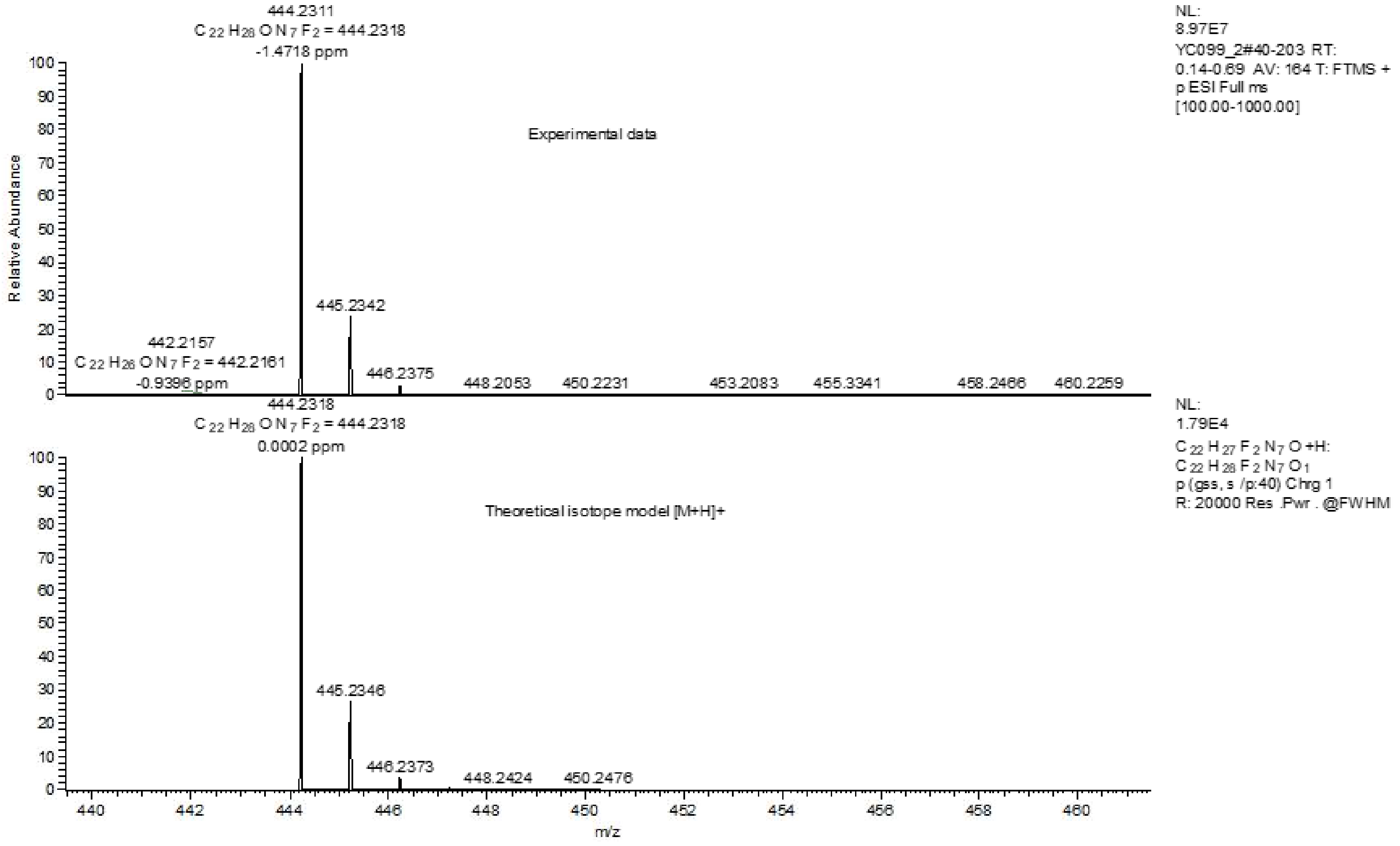

Figure S19-4: HRMS spectrum of Compound **9.**

2-(2,4-Difluorophenyl)-1-(4-((5-fluoro-4-methylpyrimidin-2-yl)amino)piperidin-1-yl)-3-(1H-1,2,4-triazol-1-yl)propan-2-ol (10)

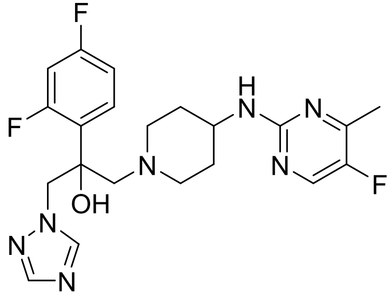

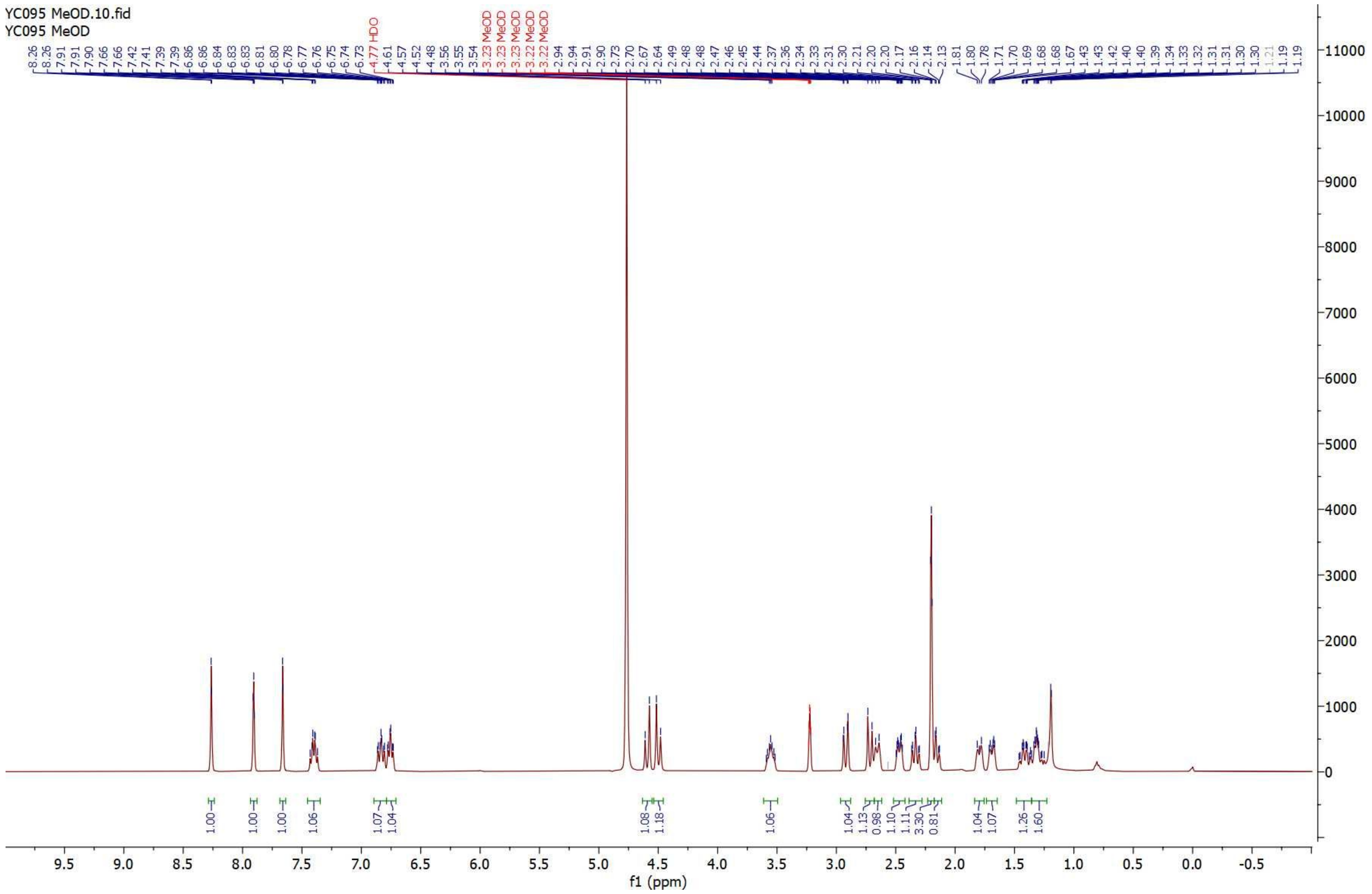

Figure S10-1: Proton ^{1}H NMR spectrum of Compound **10.**

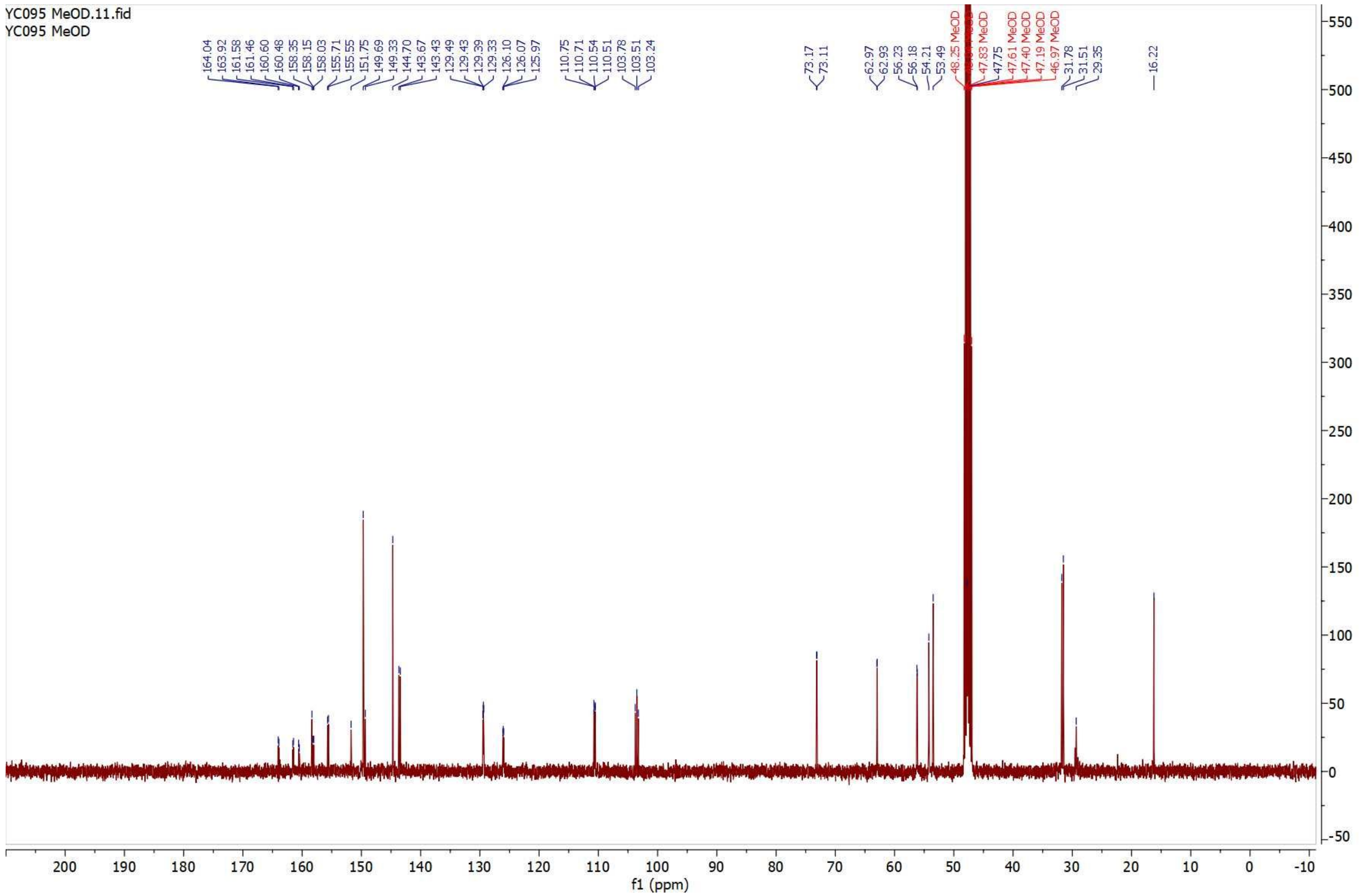

Figure S10-2: Carbon ^{13}C NMR spectrum of Compound **10.**

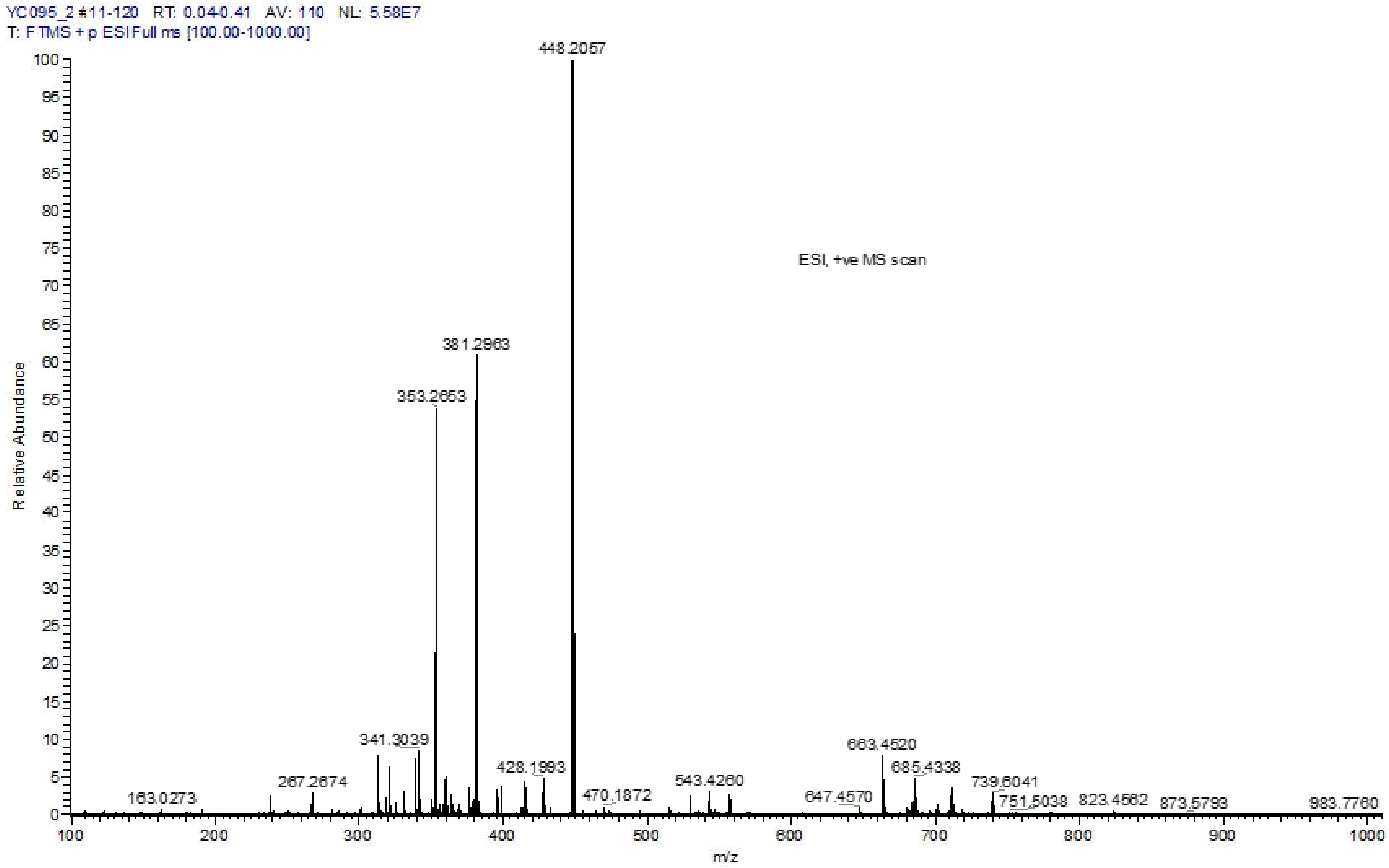

Figure S10-3: HRMS spectrum of Compound **10.**

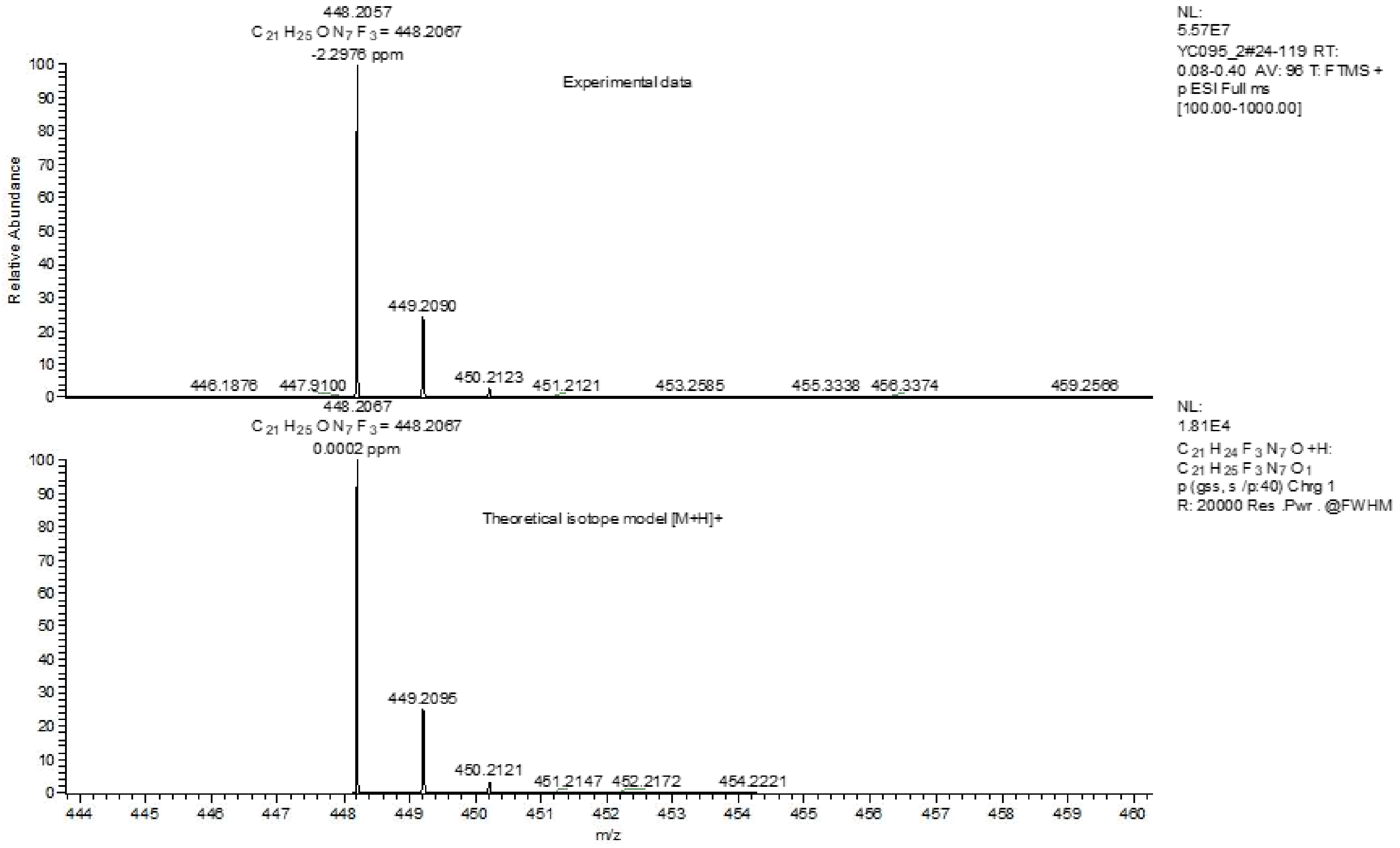

Figure S10-4: HRMS spectrum of Compound **10.**

2-(2,4-difluorophenyl)-1-(4-(pyrimidin-5-ylamino)piperidin-1-yl)-3-(1H-1,2,4-triazol-1-yl)propan-2-ol (11)

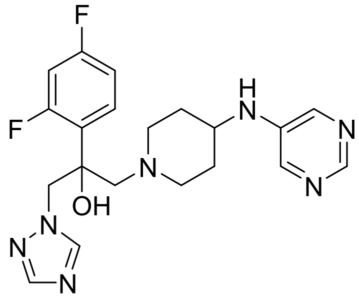

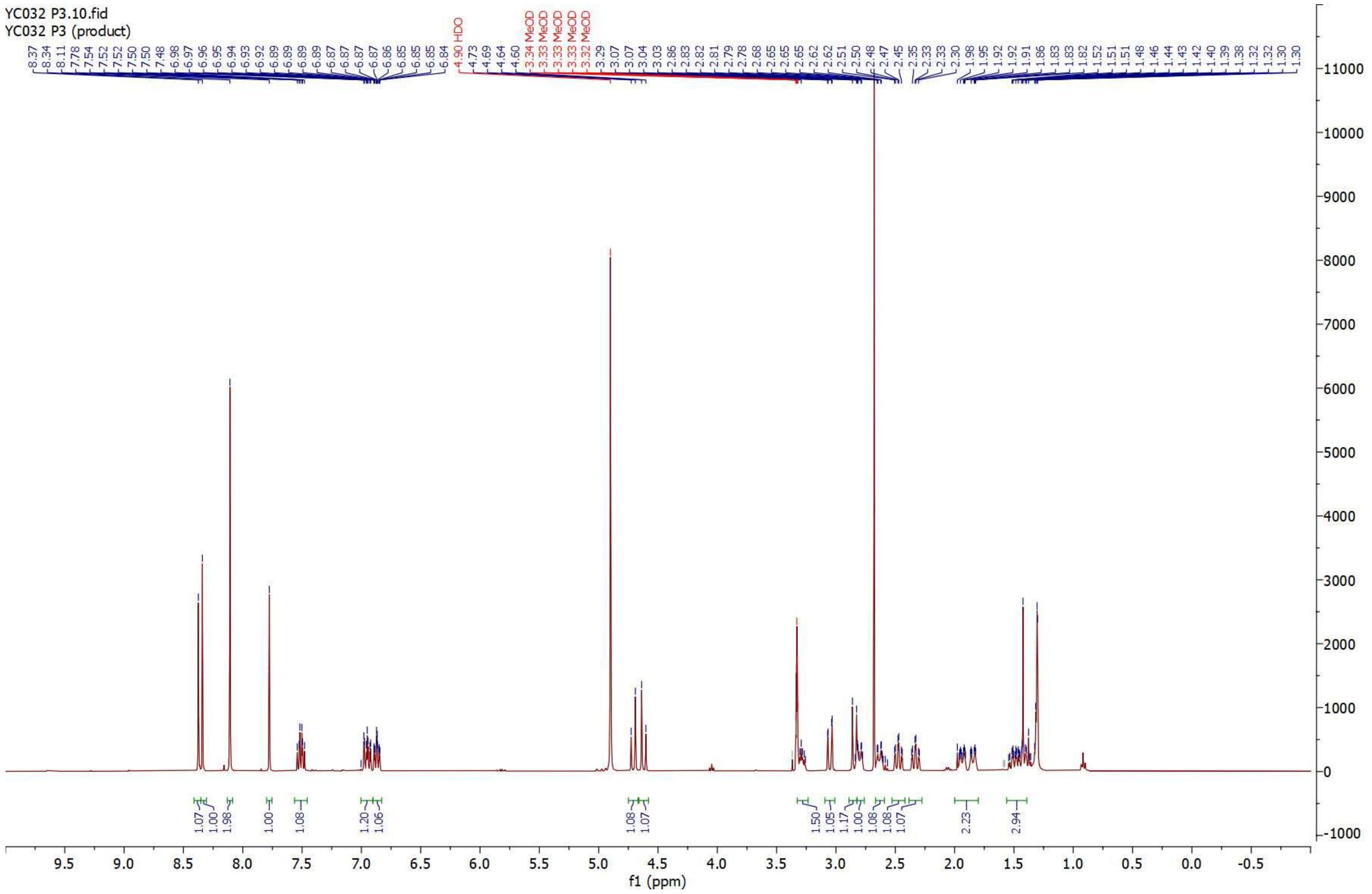

Figure S11-1: Proton ^{1}H NMR spectrum of Compound **11.**

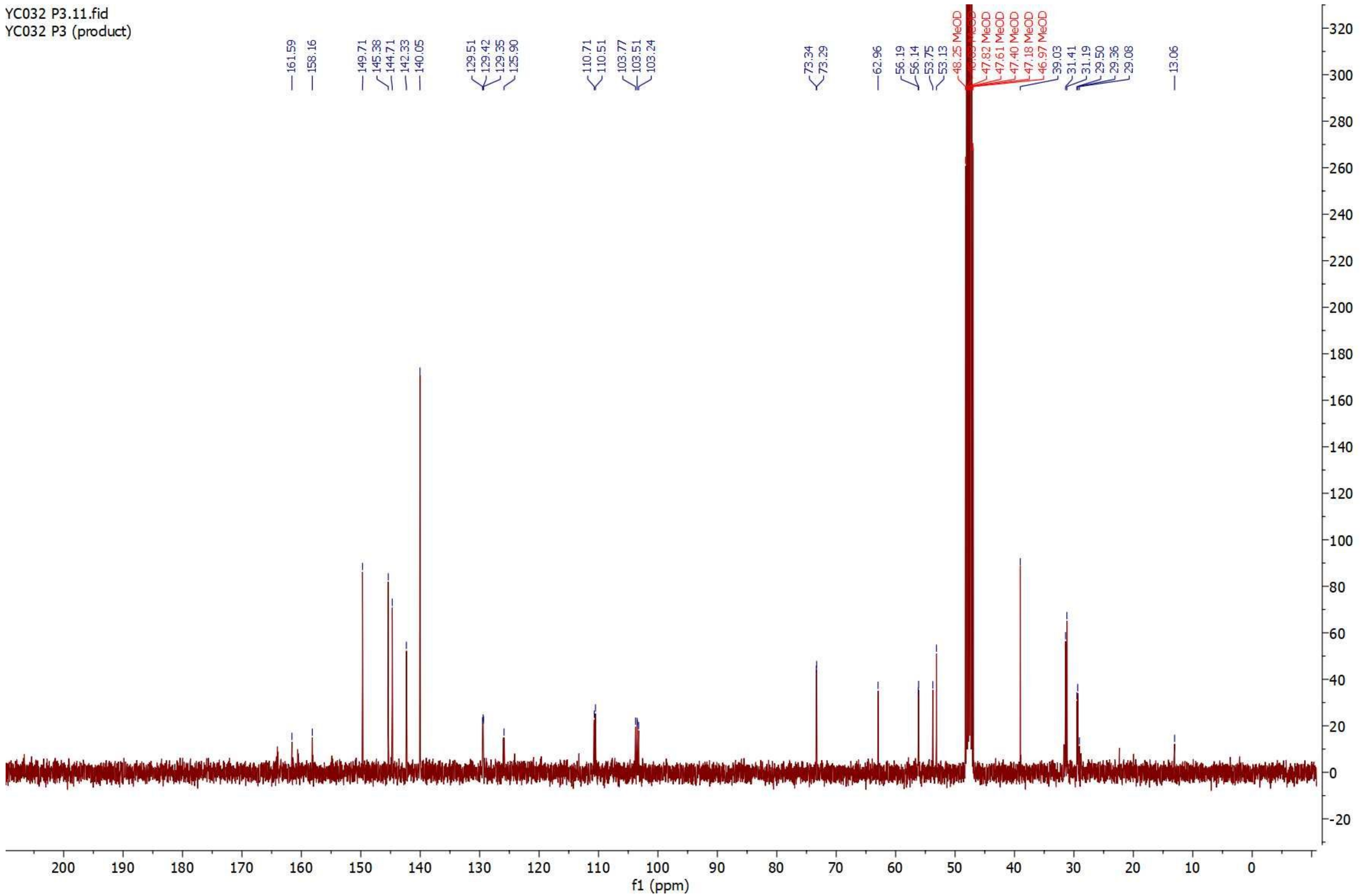

Figure S11-2: Carbon ^{13}C NMR spectrum of Compound **11.**

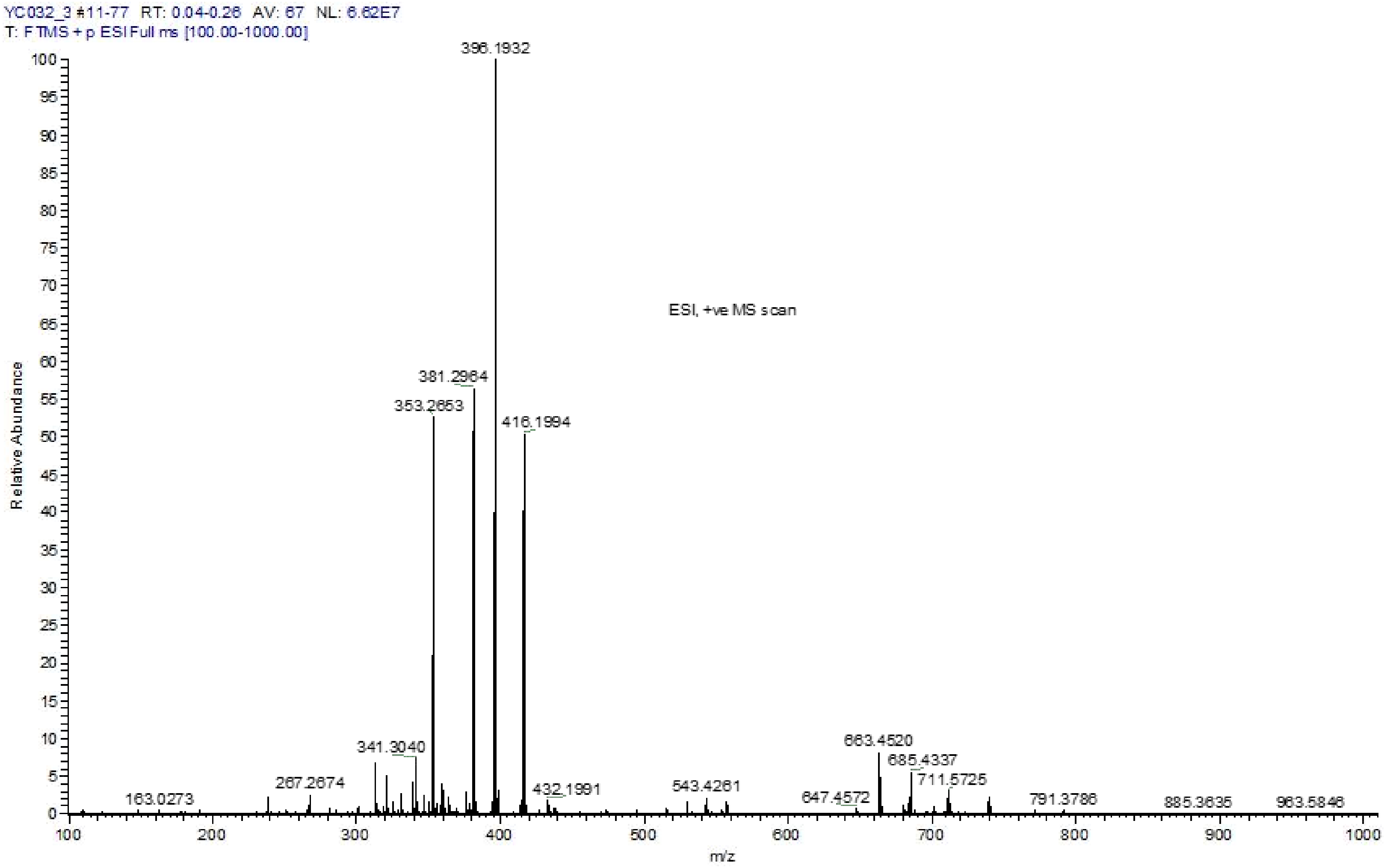

Figure S11-3: HRMS spectrum of Compound **11.**

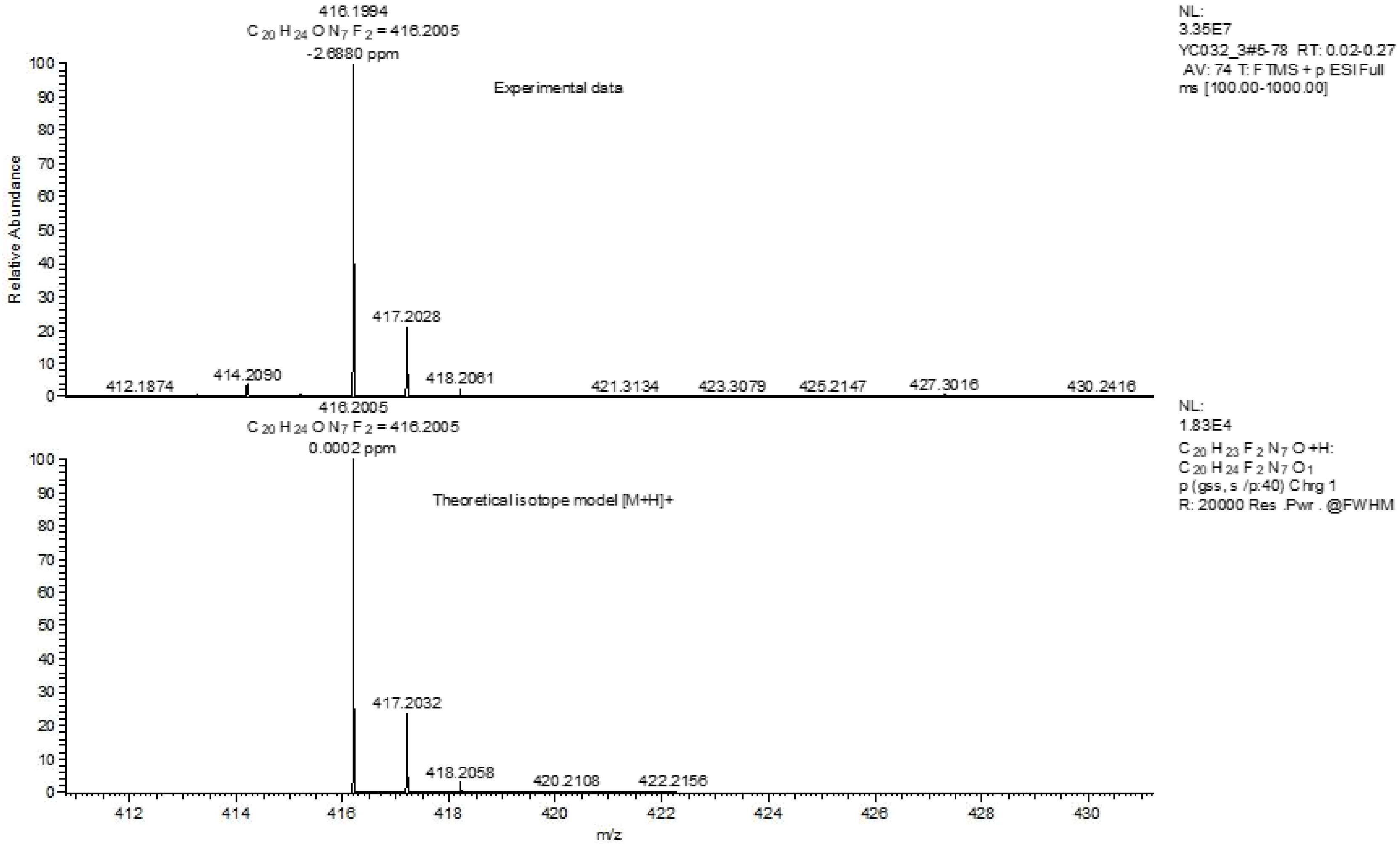

Figure S11-4: HRMS spectrum of Compound **11.**

2-(2,4-difluorophenyl)-1-(4-((2-fluoropyrimidin-5-yl)amino)piperidin-1-yl)-3-(1H-1,2,4-triazol-1-yl)propan-2-ol (12)

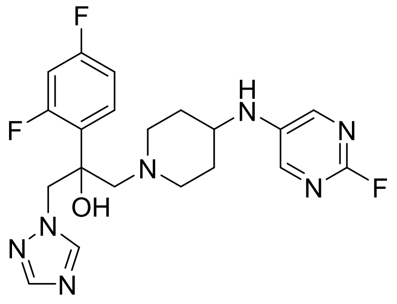

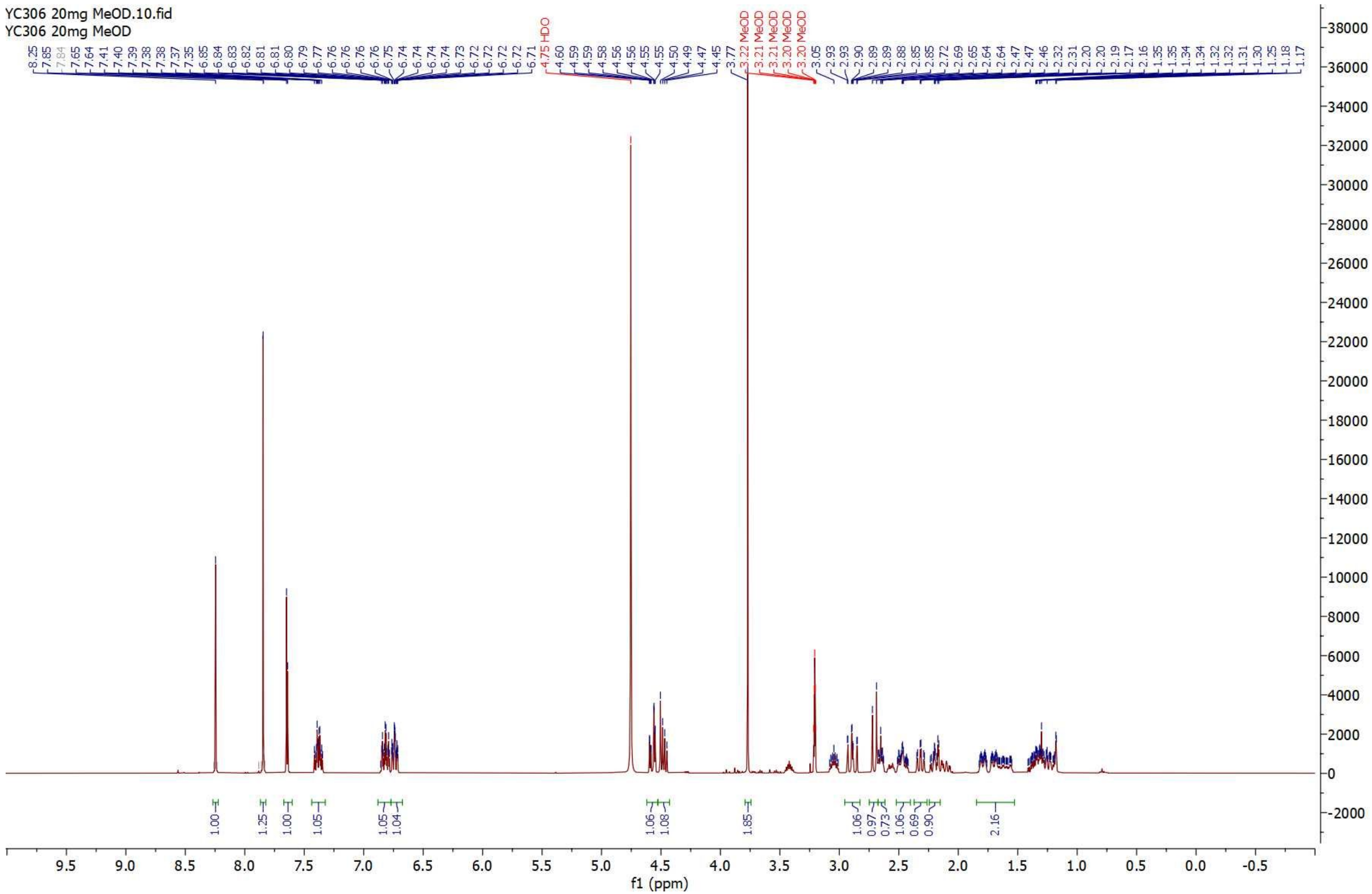

Figure S12-1: Proton ^{1}H NMR spectrum of Compound **12.**

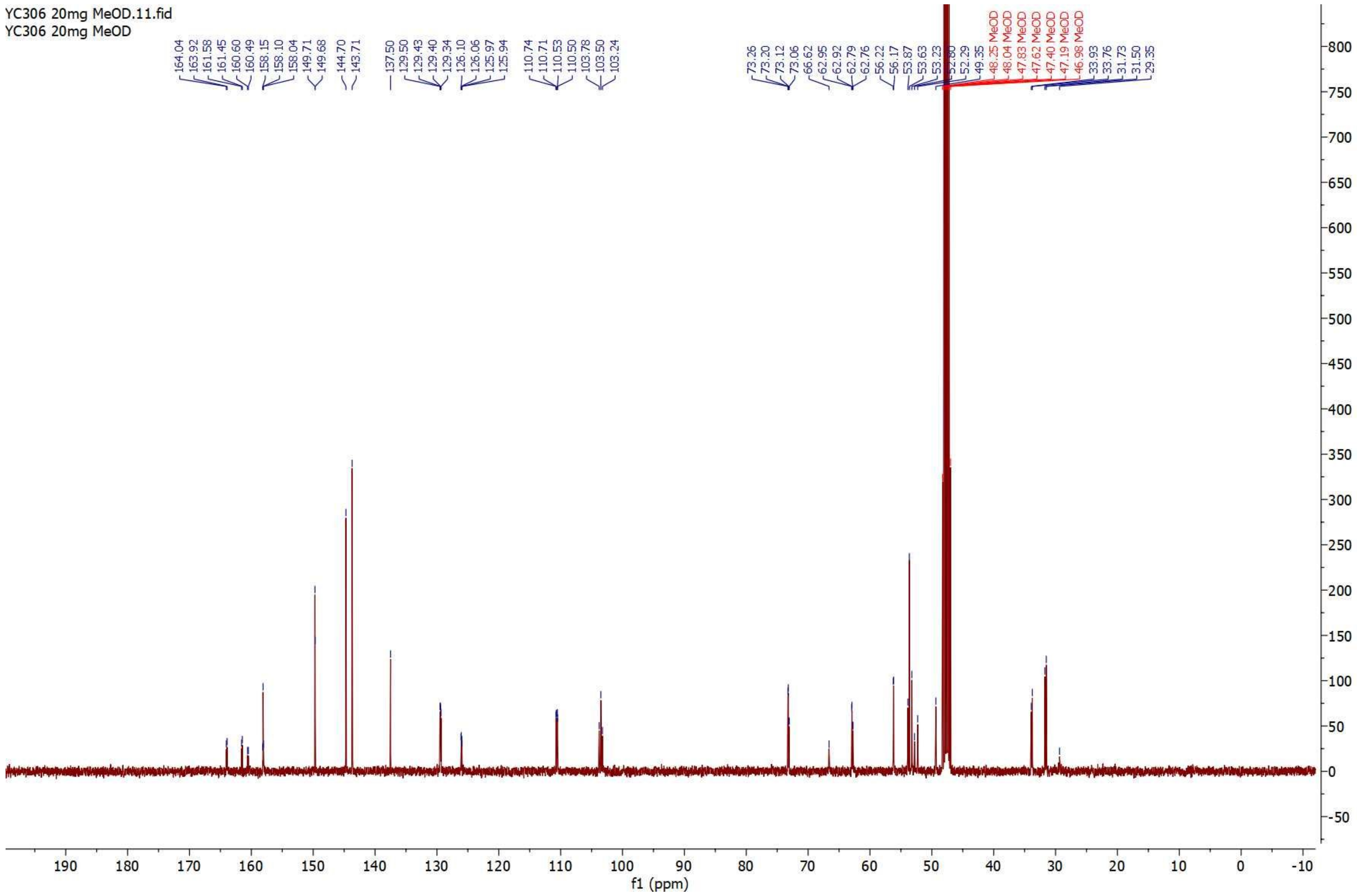

Figure S12-2: Carbon ^{13}C NMR spectrum of Compound **12.**

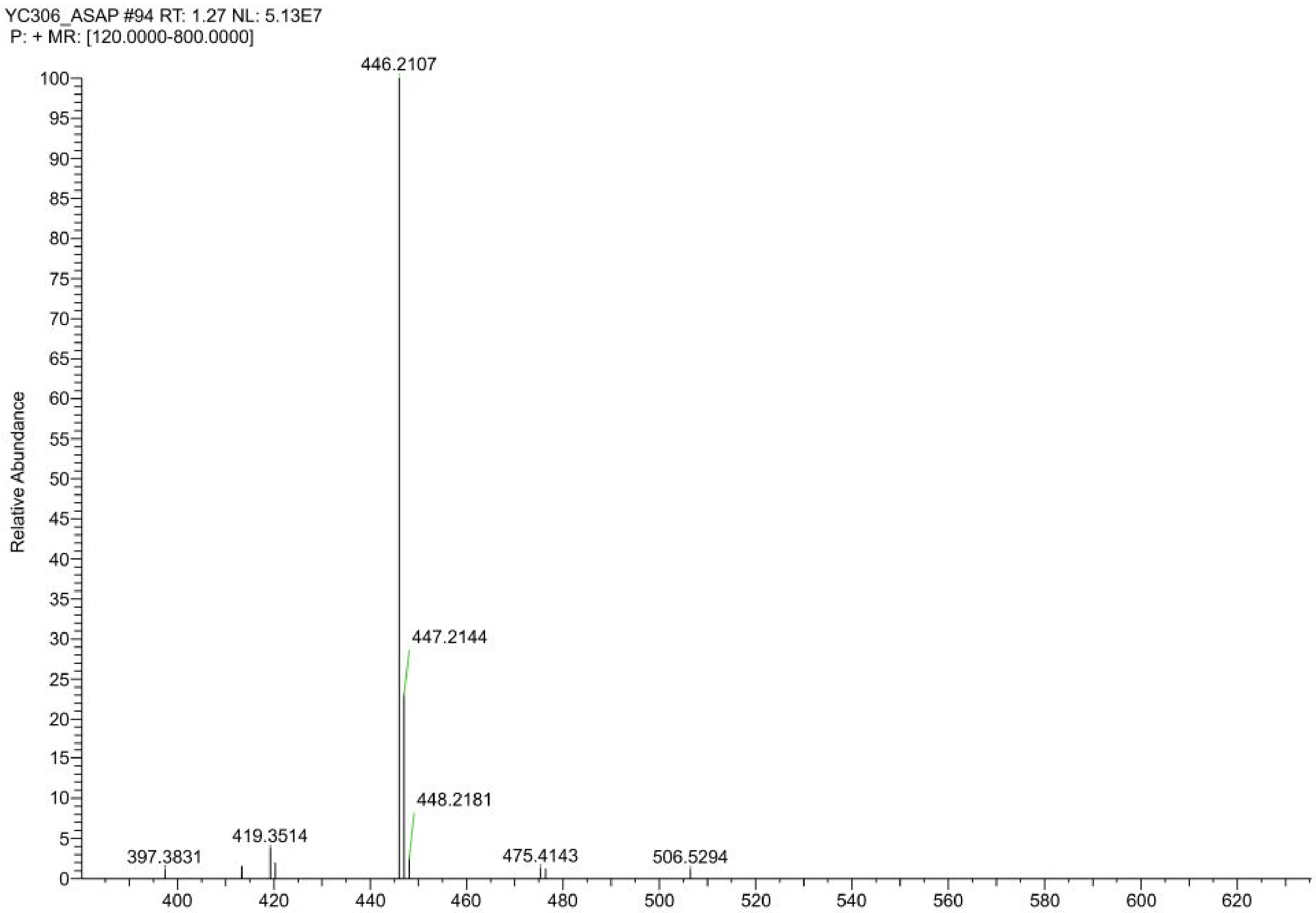

Figure S12-3: HRMS spectrum of Compound **12.**

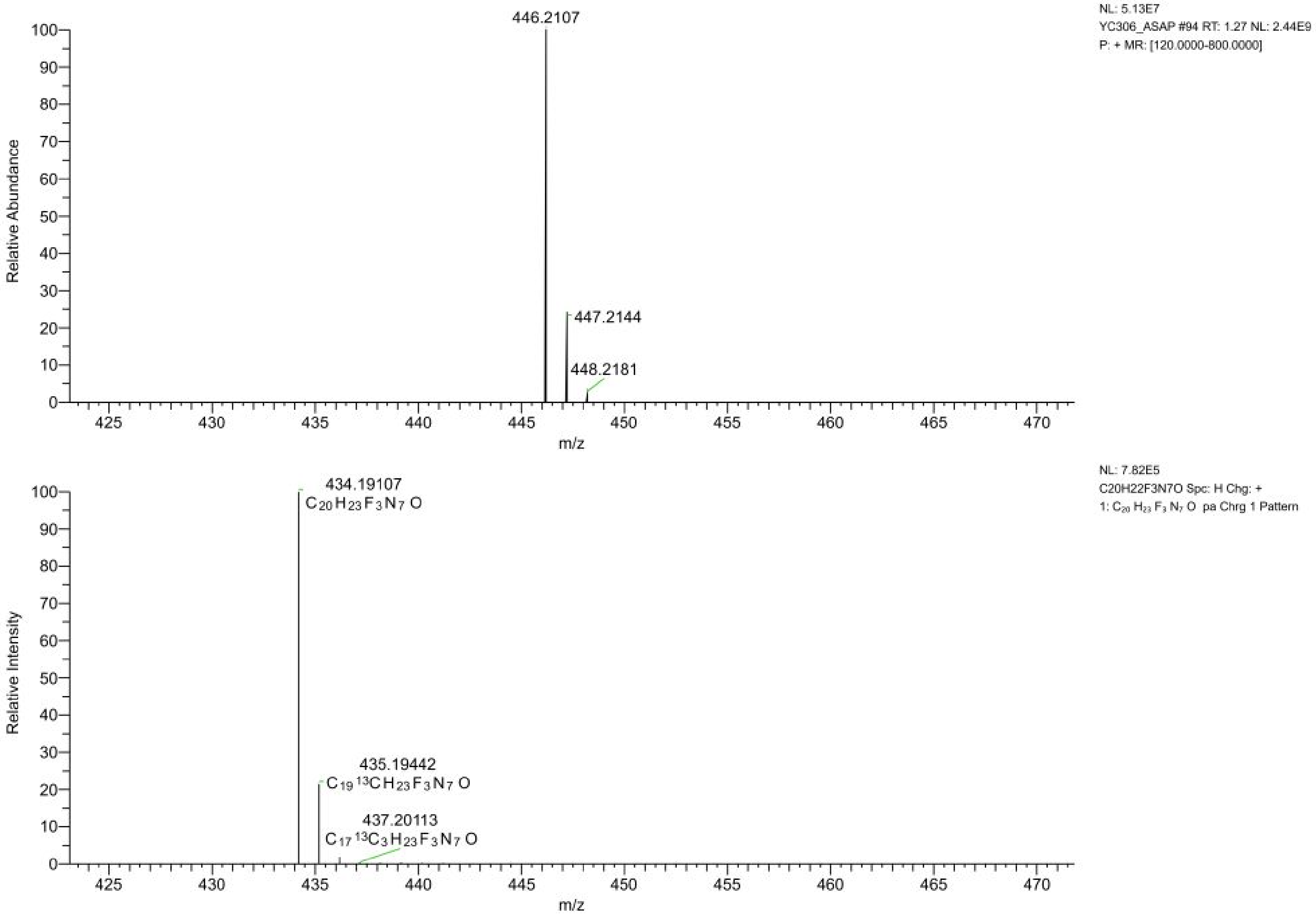

Figure S12-4: HRMS spectrum of Compound **12.**

2-(2,4-difluorophenyl)-1-(4-(5-fluoropyrimidin-2-yl)piperazin-1-yl)-3-(1H-1,2,4-triazol-1-yl)propan-2-ol (15)

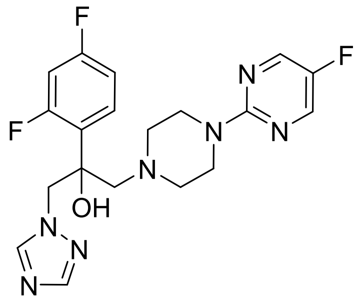

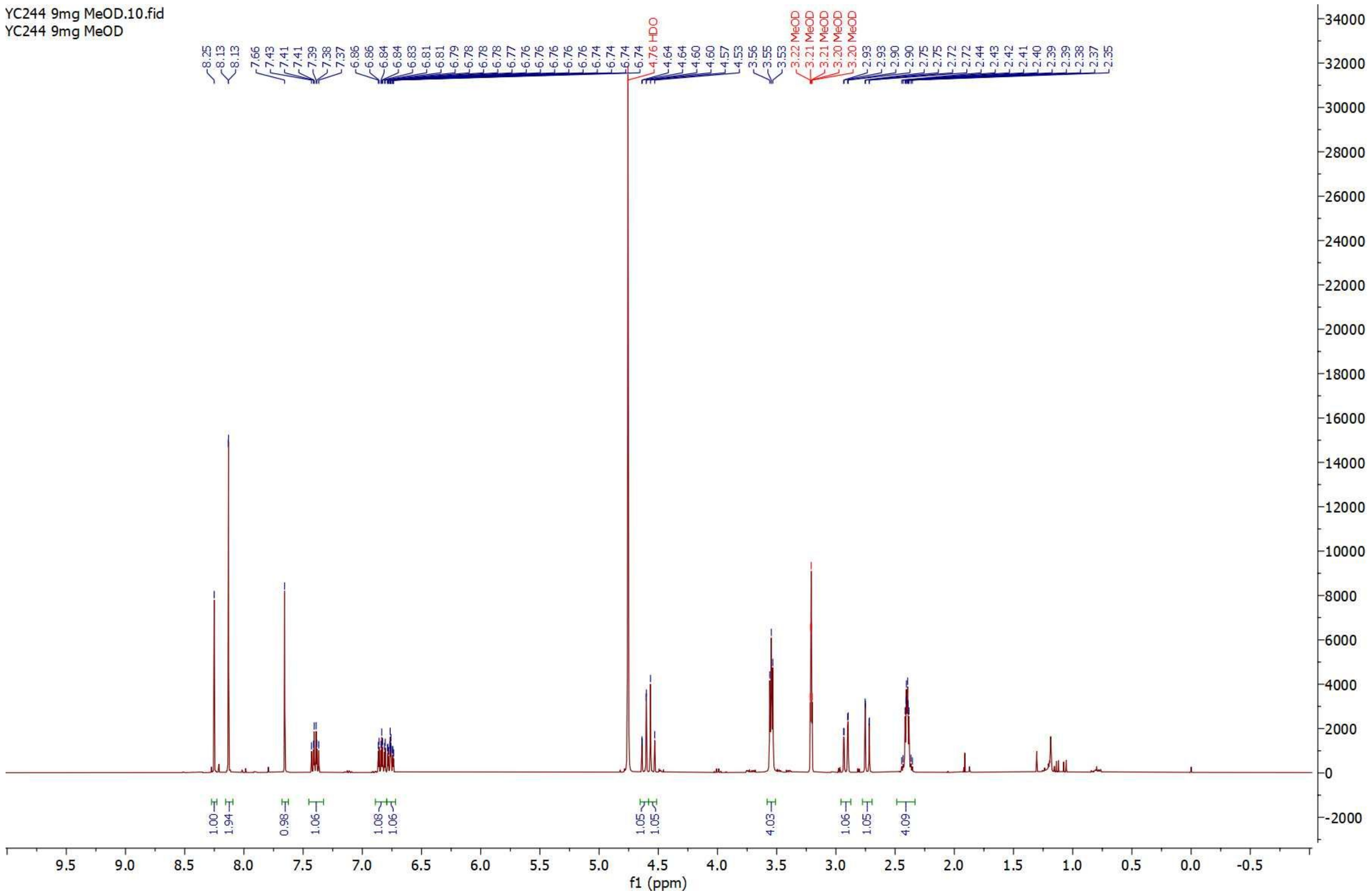

Figure S13-1: Proton ^{1}H NMR spectrum of Compound **15.**

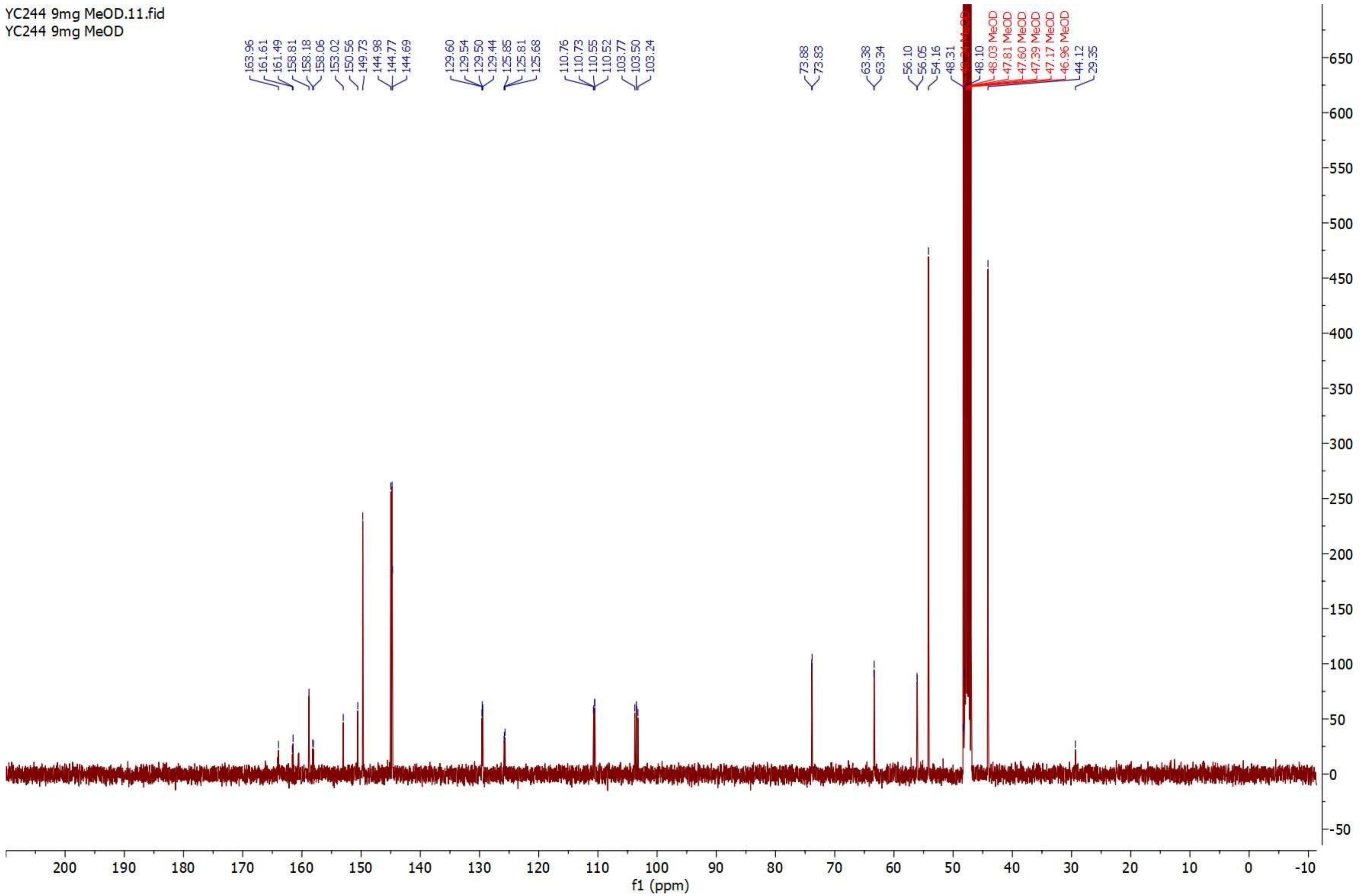

Figure S13-2: Carbon ^{13}C NMR spectrum of Compound **15.**

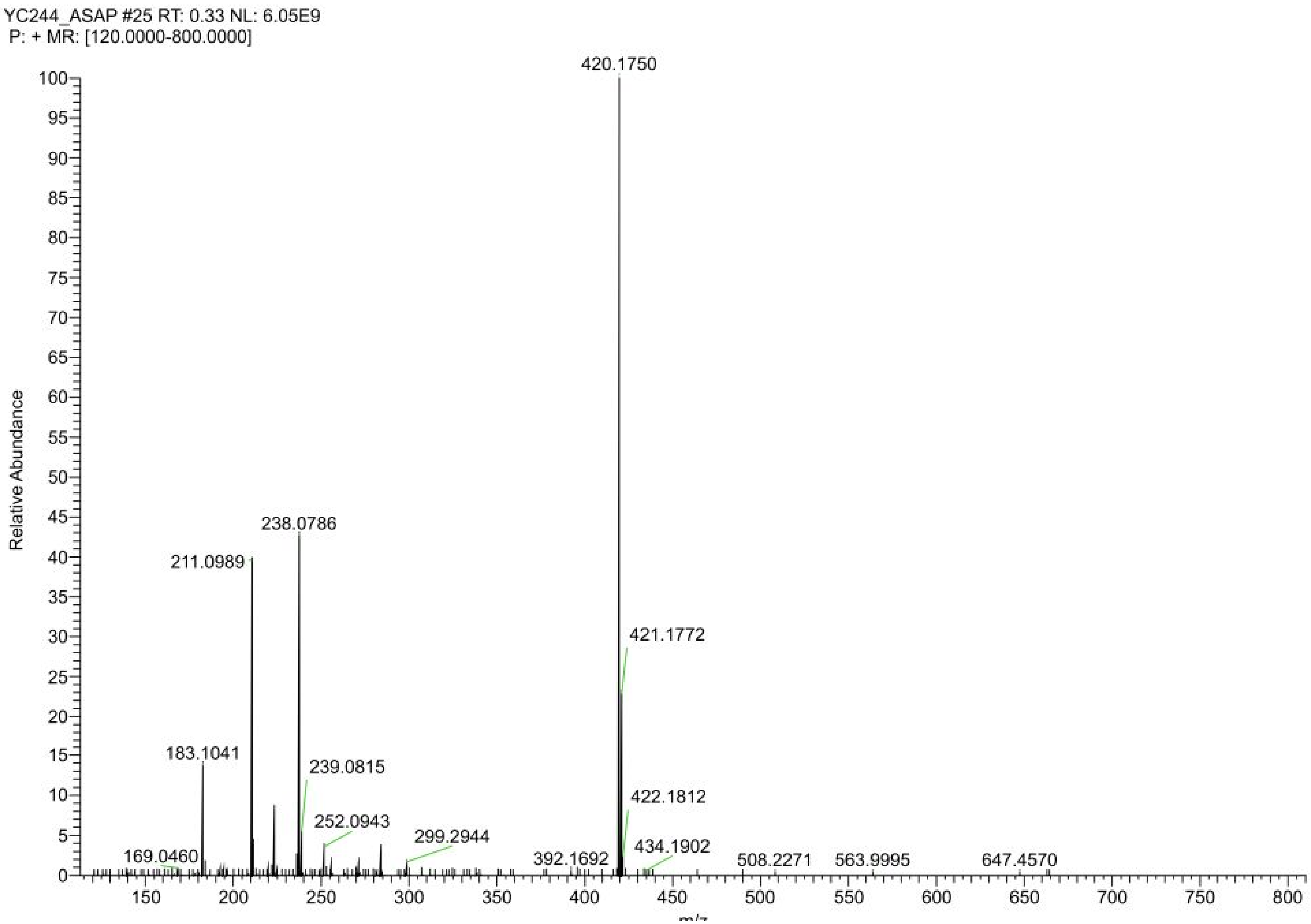

Figure S13-3: HRMS spectrum of Compound **15.**

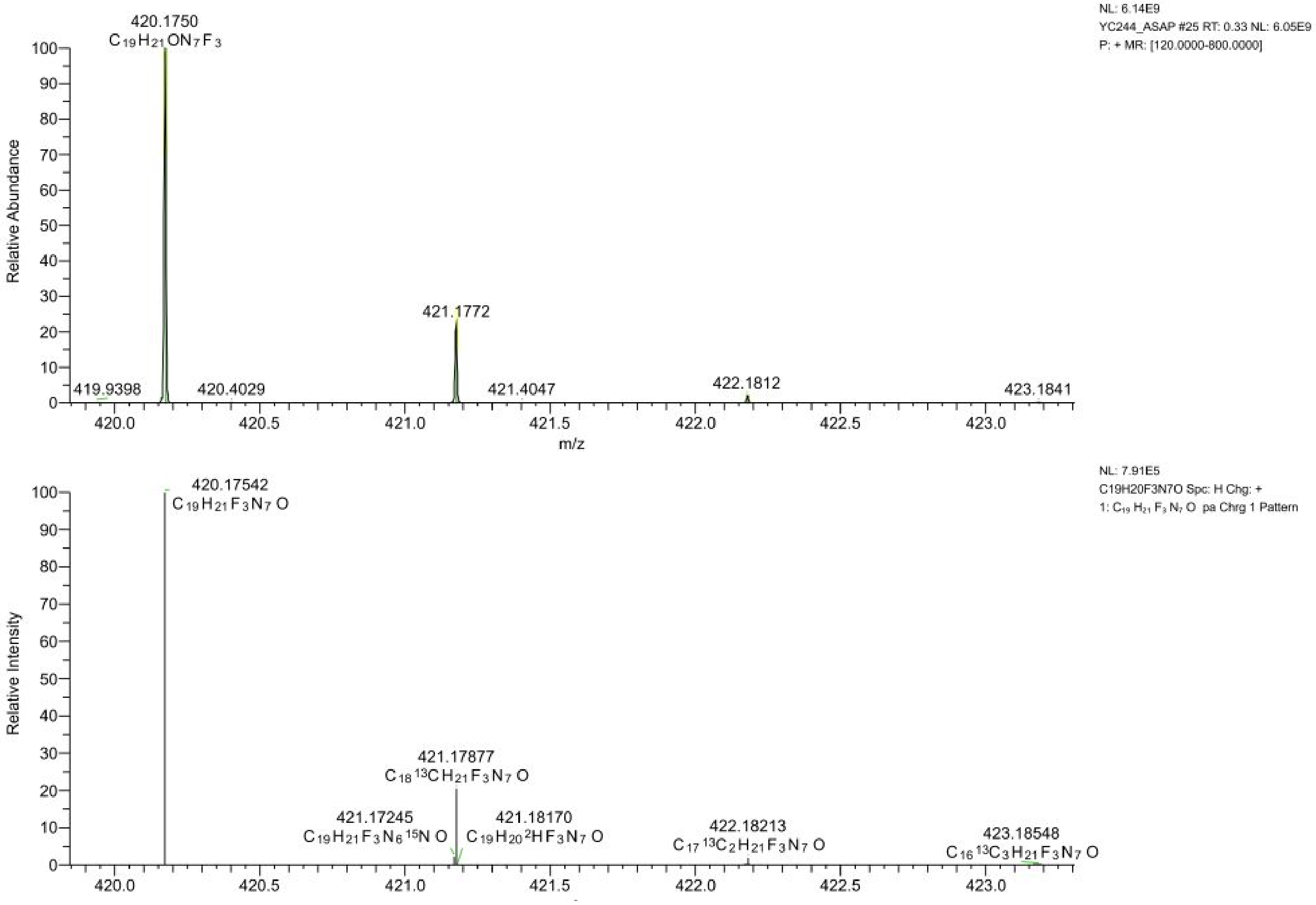

Figure S13-4: HRMS spectrum of Compound **15.**

2-(2,4-Difluorophenyl)-1-(4-((5-fluoropyrimidin-2-yl)amino)-[1,4'-bipiperidin]-1'-yl)-3-(1H-1,2,4-triazol-1-yl)propan-2-ol (16)

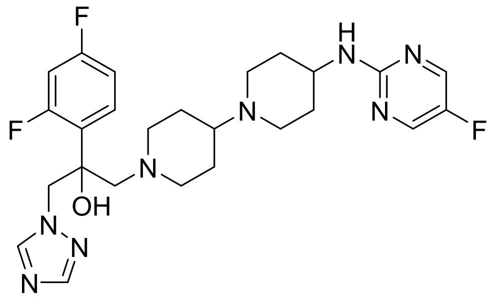

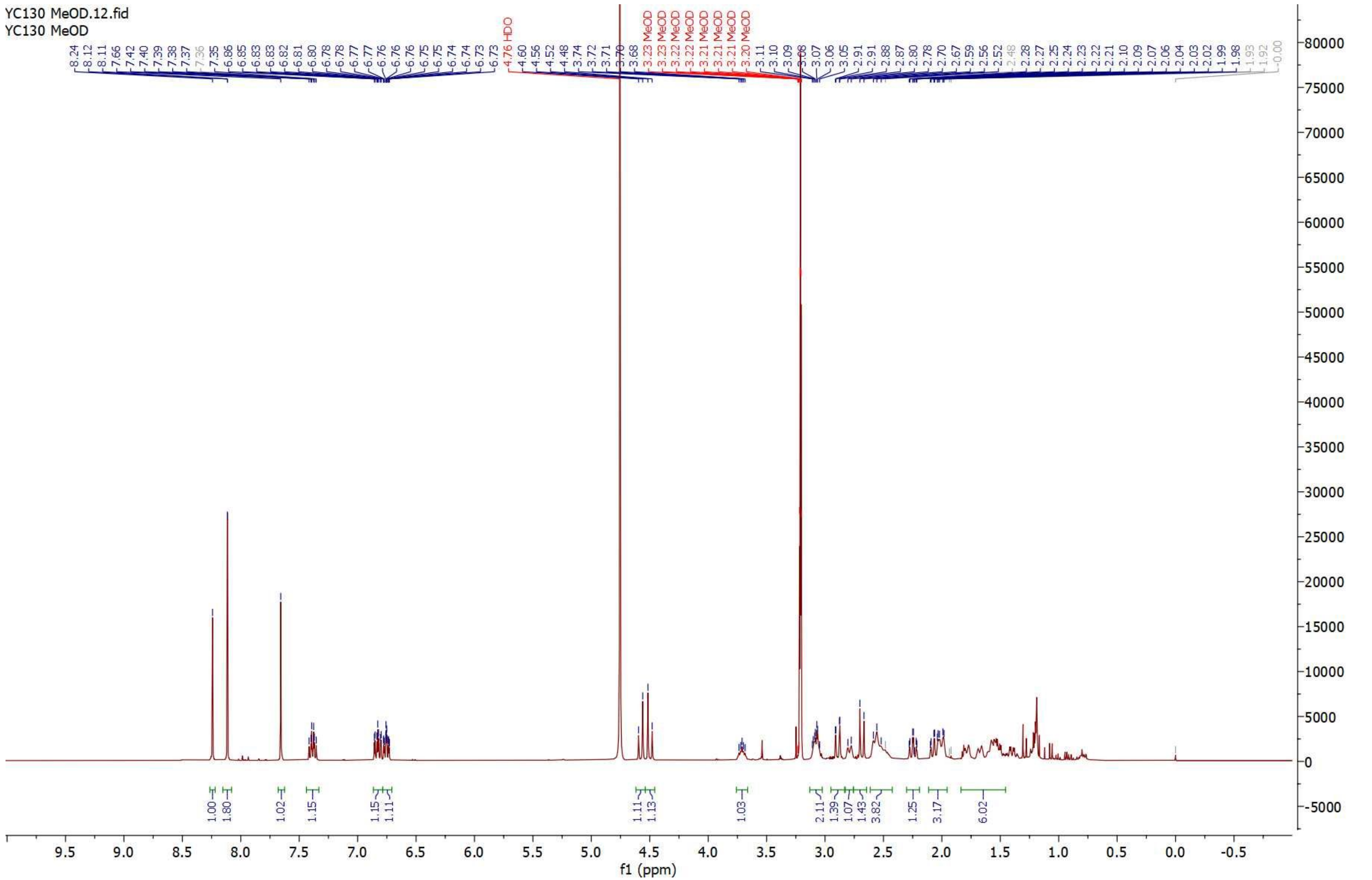

Figure S14-1: Proton ^{1}H NMR spectrum of Compound **16.**

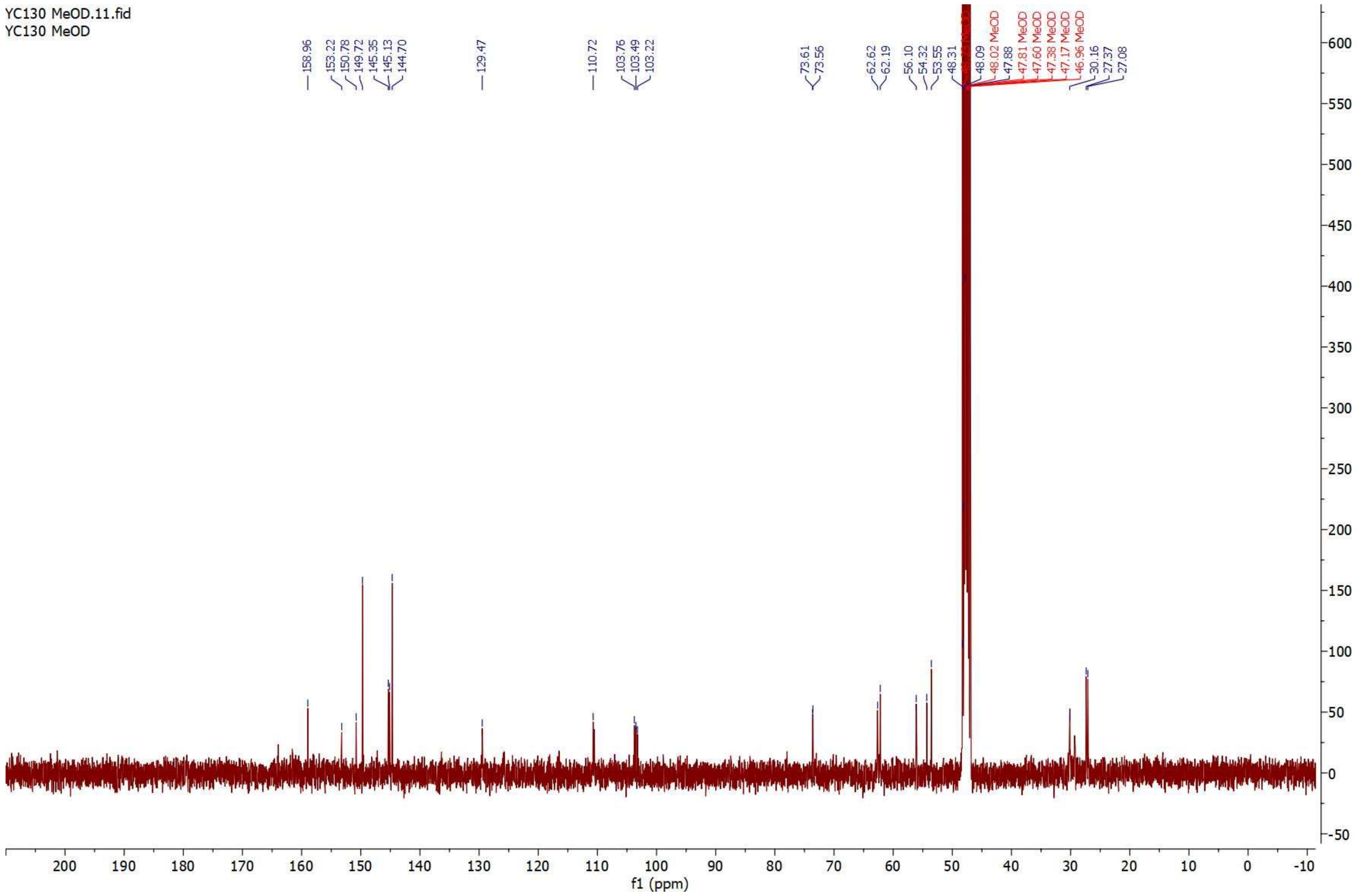

Figure S14-2: Carbon ^{13}C NMR spectrum of Compound **16.**

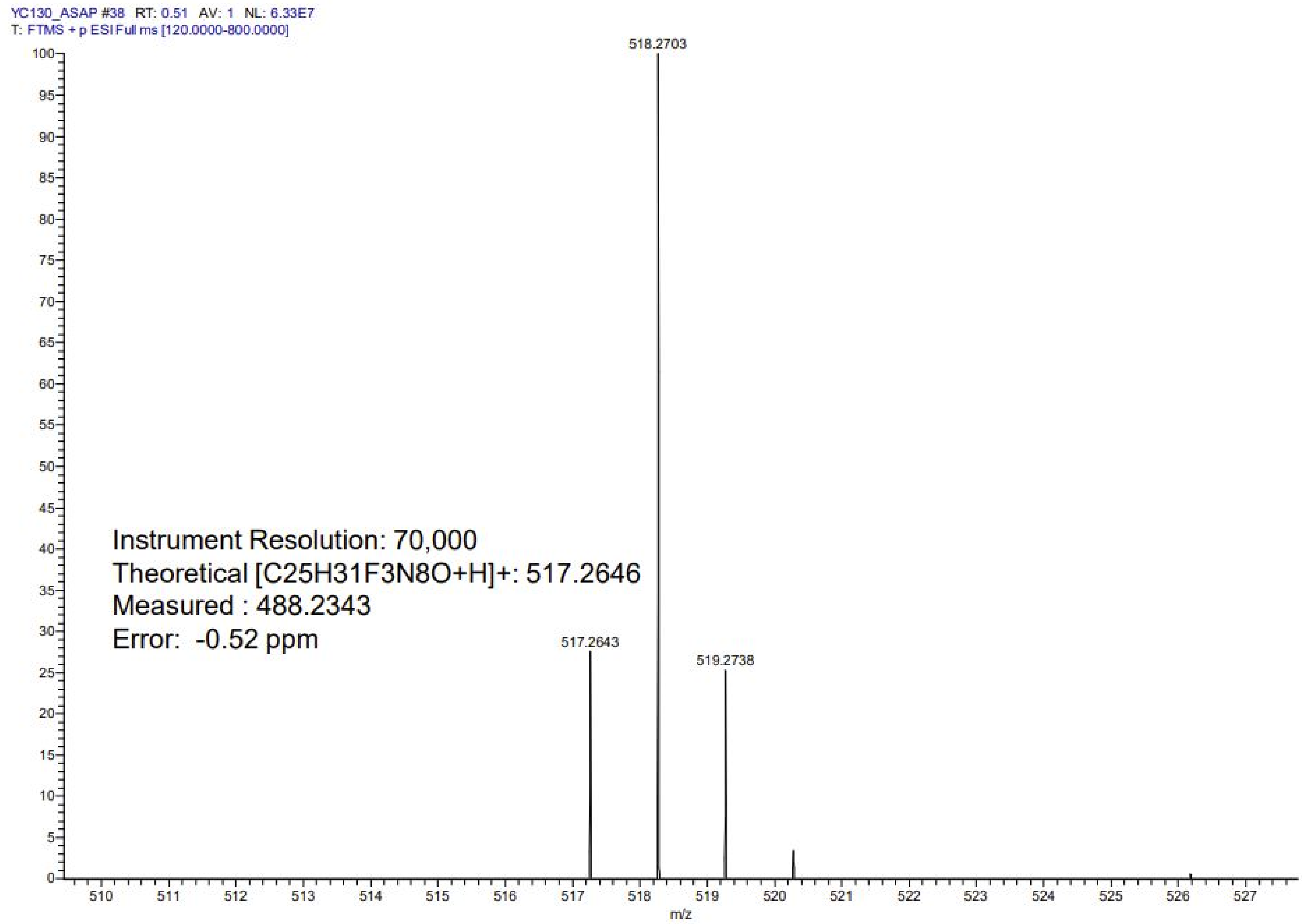

Figure S14-3: HRMS spectrum of Compound **16.**

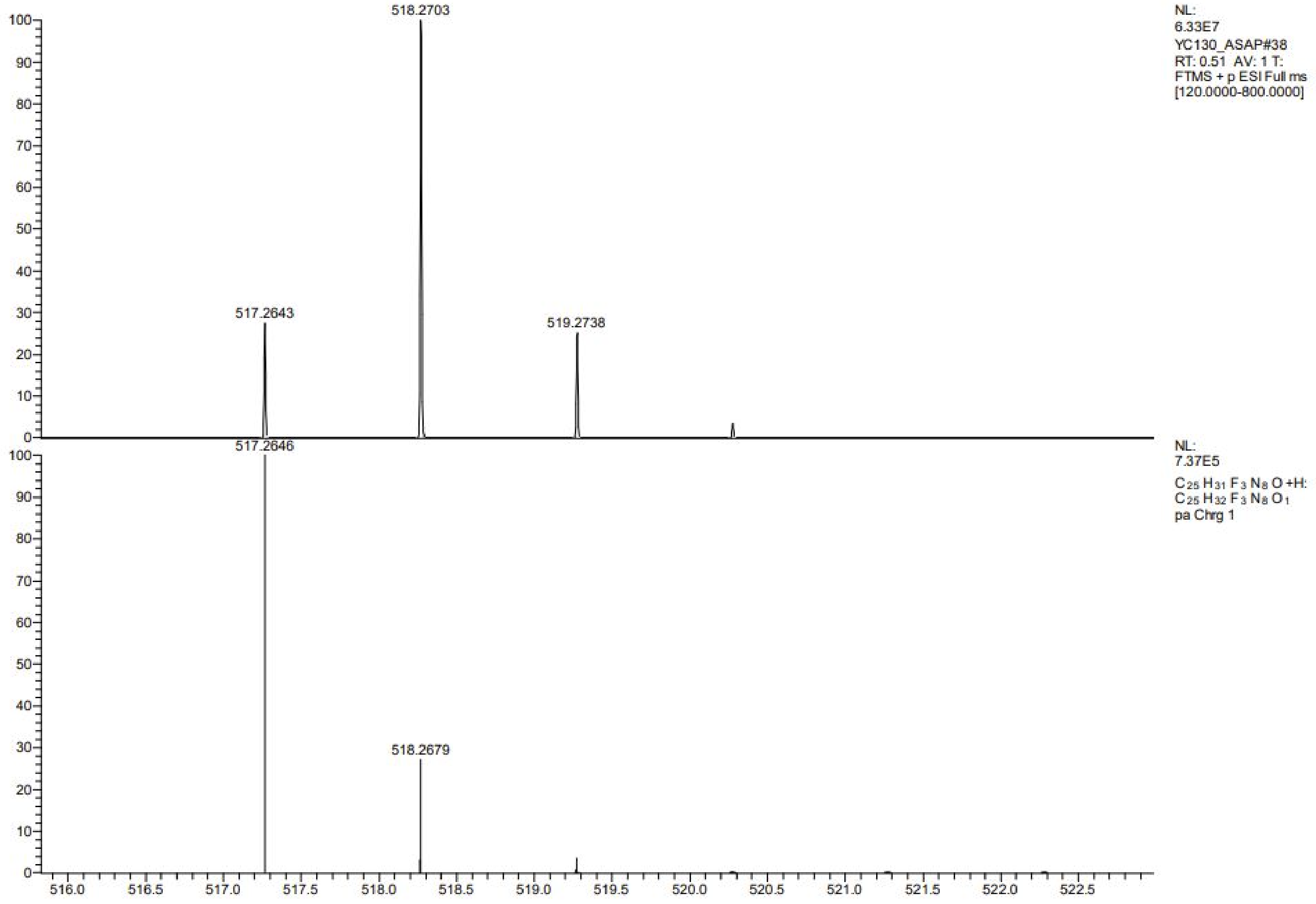

Figure S14-4: HRMS spectrum of Compound **16.**

2-(2,4-difluorophenyl)-1-(8-((5-fluoropyrimidin-2-yl)amino)-2-azaspiro[4.5]decan-2-yl)-3-(1H-1,2,4-triazol-1-yl)propan-2-ol (17)

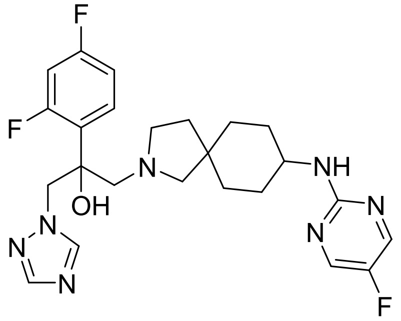

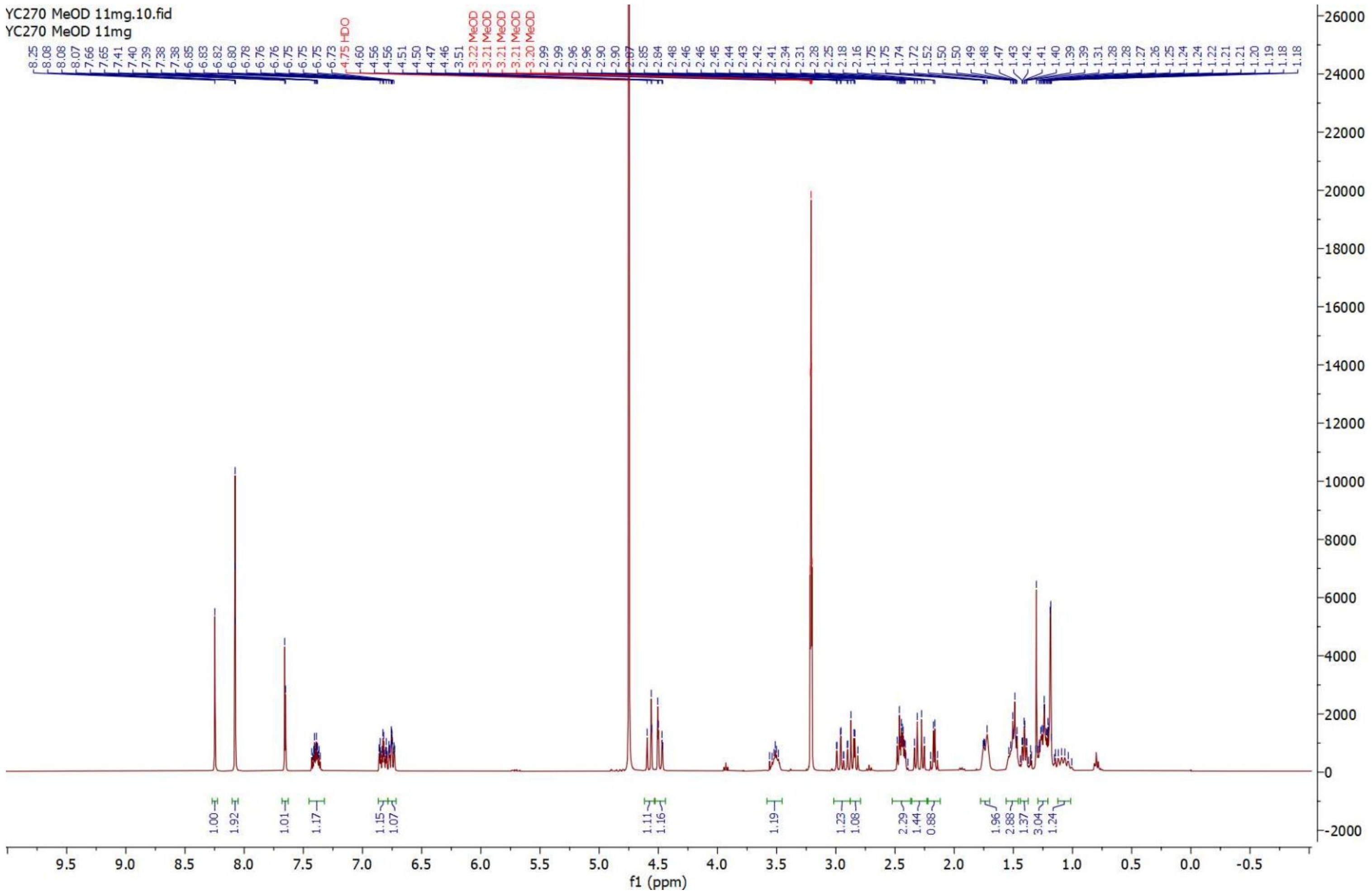

Figure S15-1: Proton ^{1}H NMR spectrum of Compound **17.**

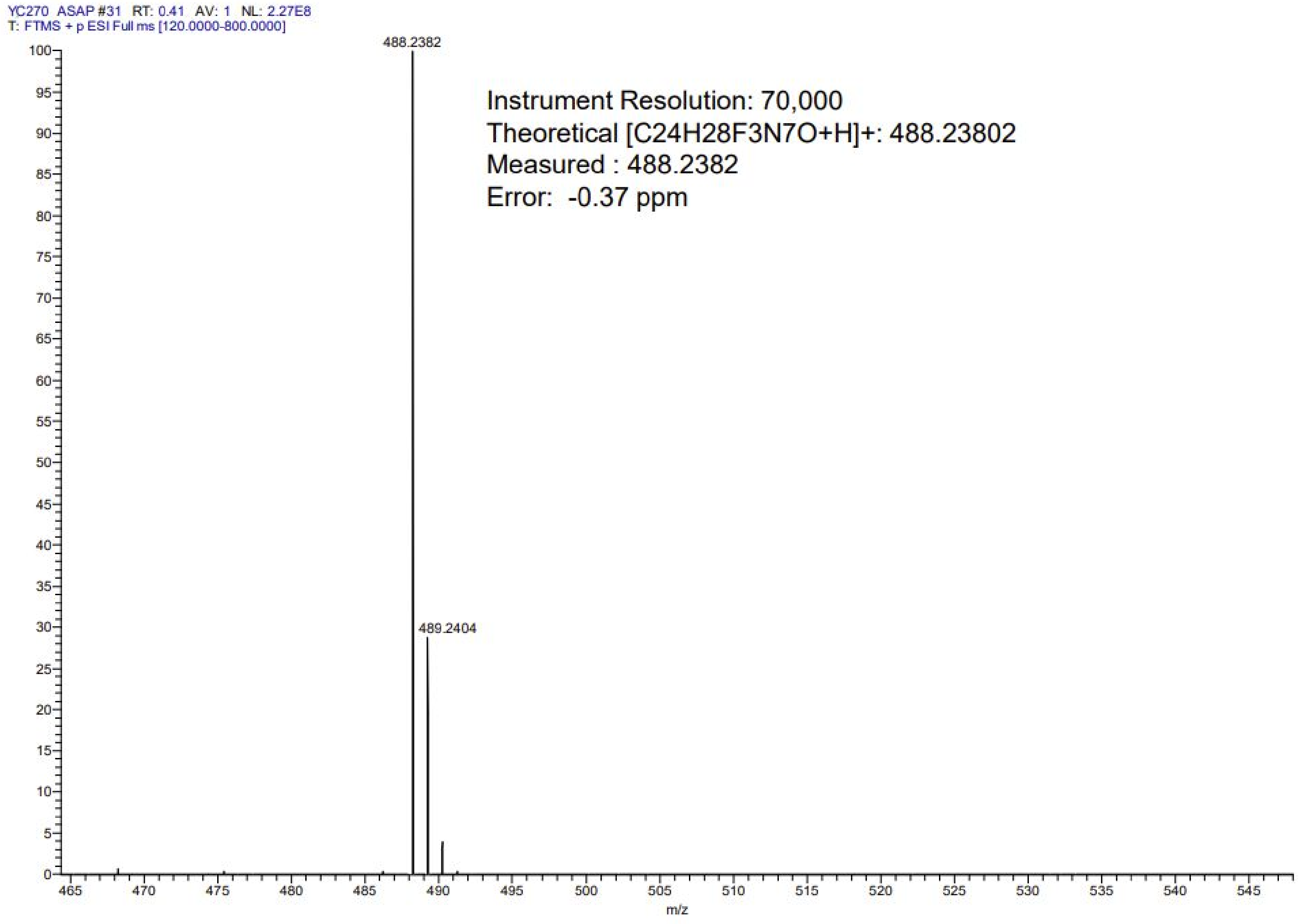

Figure S15-3: HRMS spectrum of Compound **17.**

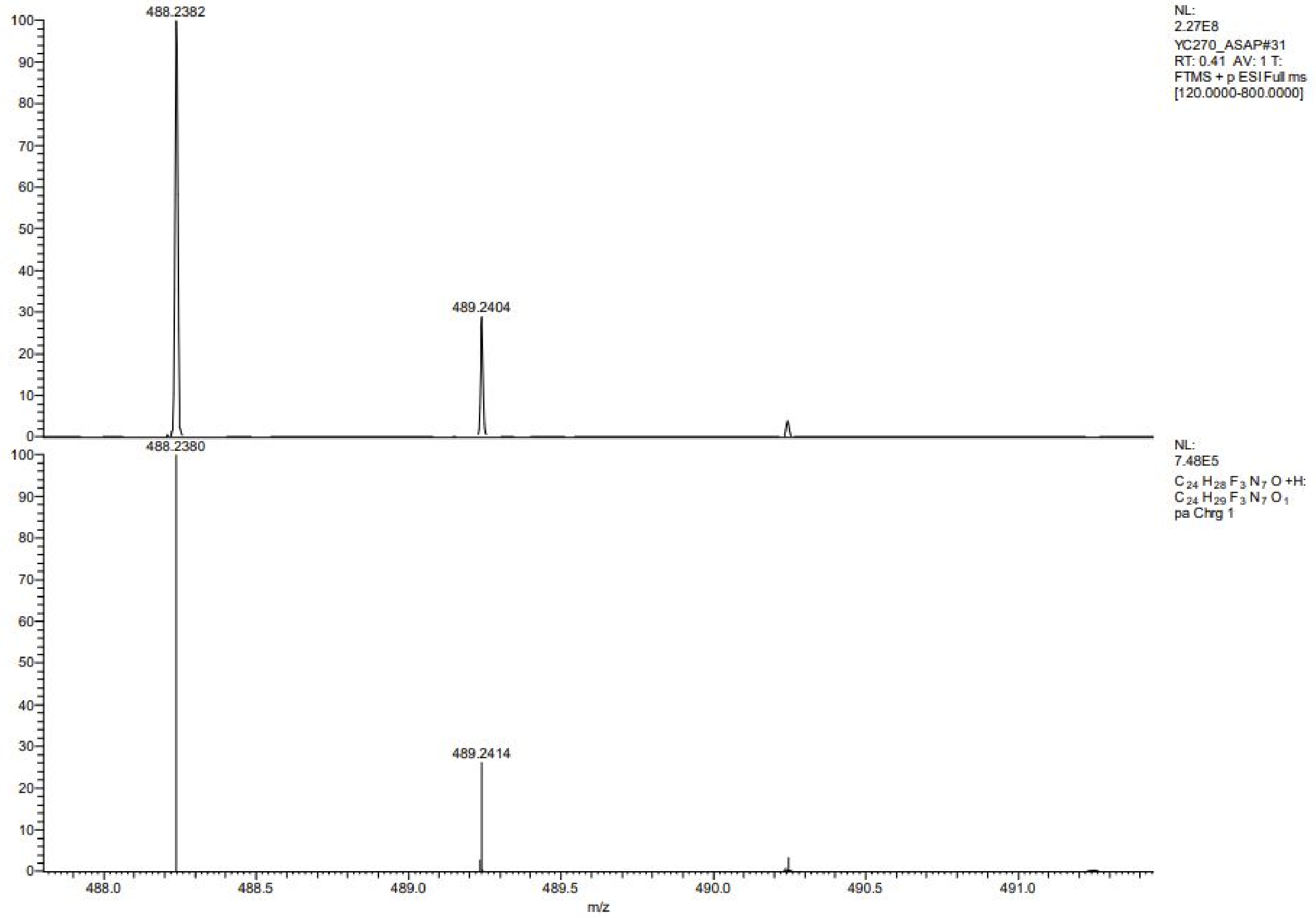

Figure S15-4: HRMS spectrum of Compound **17.**

2-(2,4-Difluorophenyl)-1-(8-(5-fluoropyrimidin-2-yl)-2,8-diazaspiro[4.5]decan-2-yl)-3-(1H-1,2,4-triazol-1-yl)propan-2-ol (18)

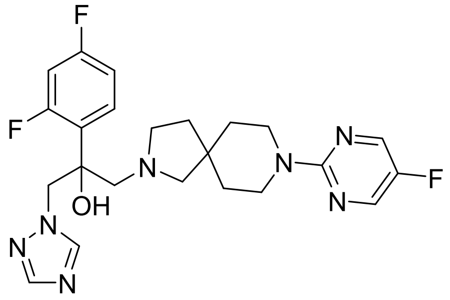

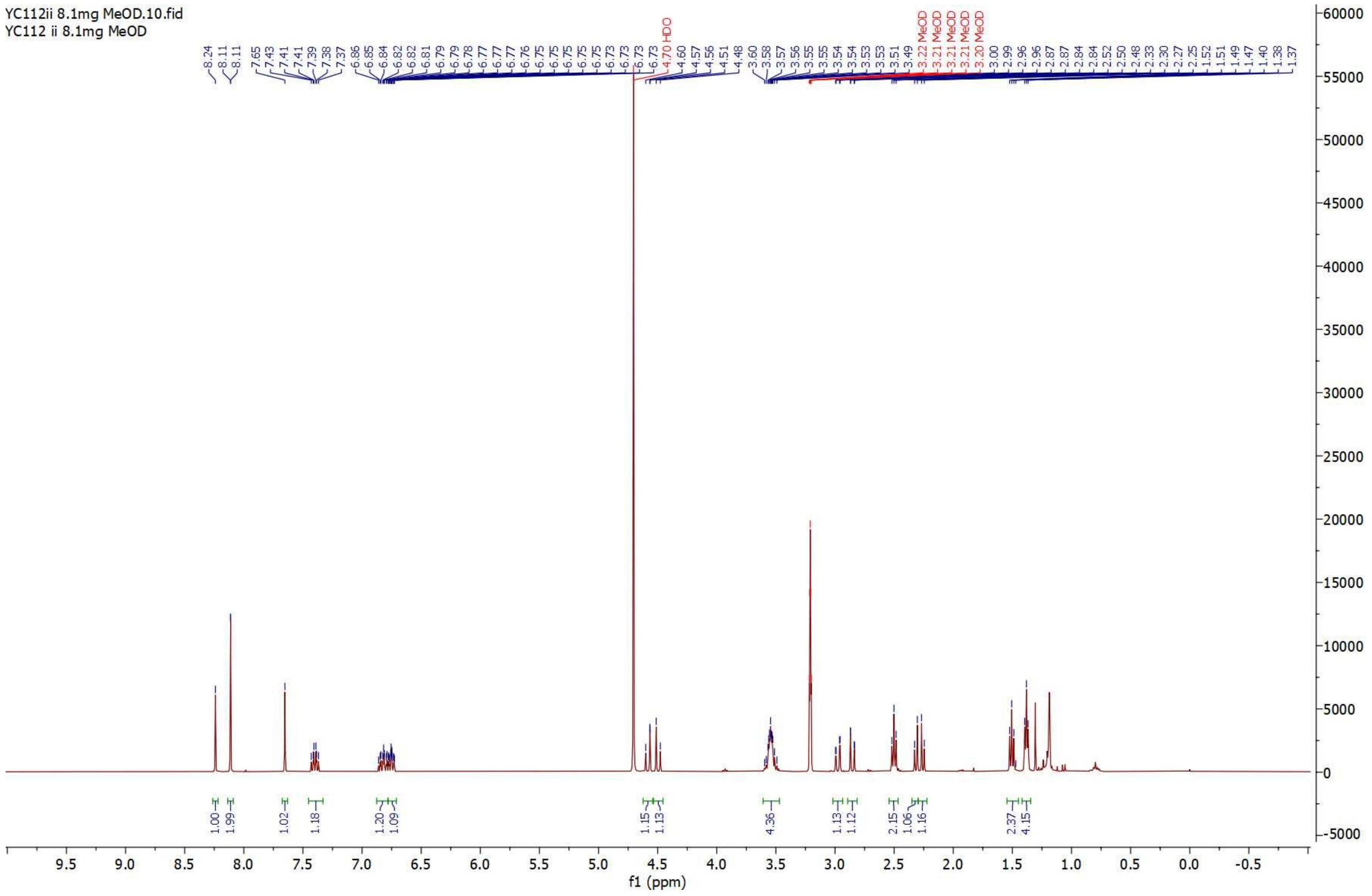

Figure S16-1: Proton ^{1}H NMR spectrum of Compound **18.**

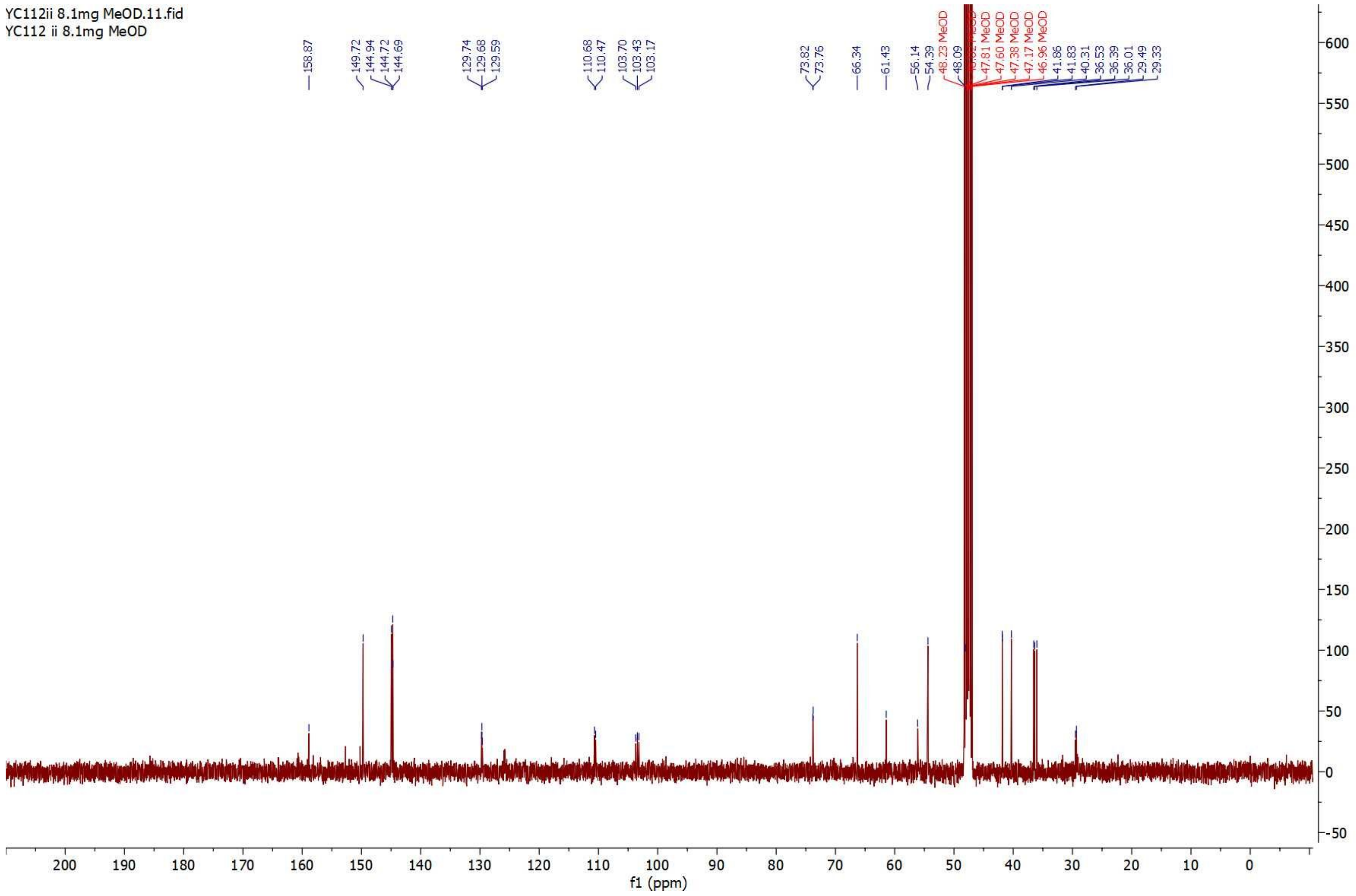

Figure S16-2: Carbon ^{13}C NMR spectrum of Compound **18.**

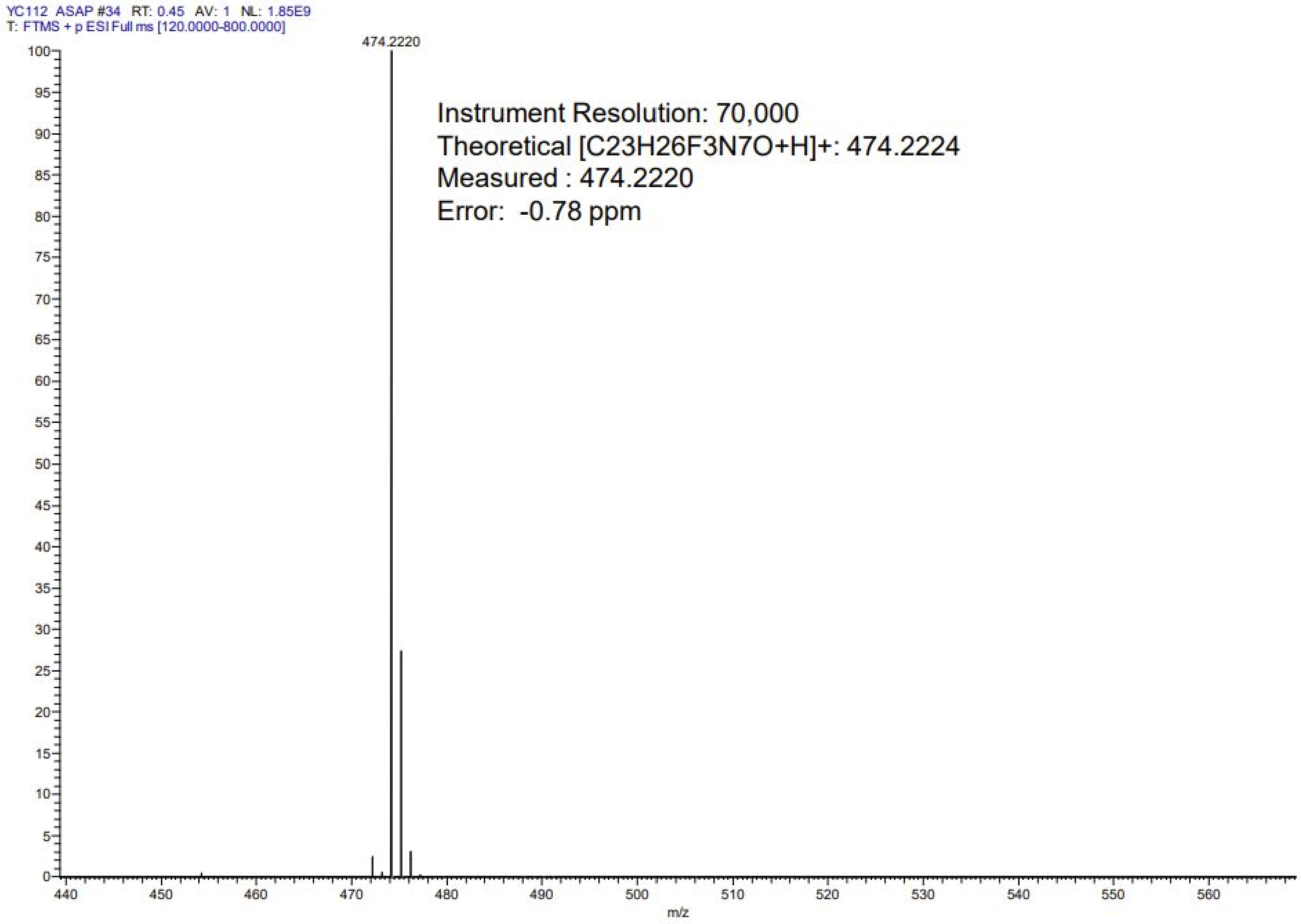

Figure S16-3: HRMS spectrum of Compound **18.**

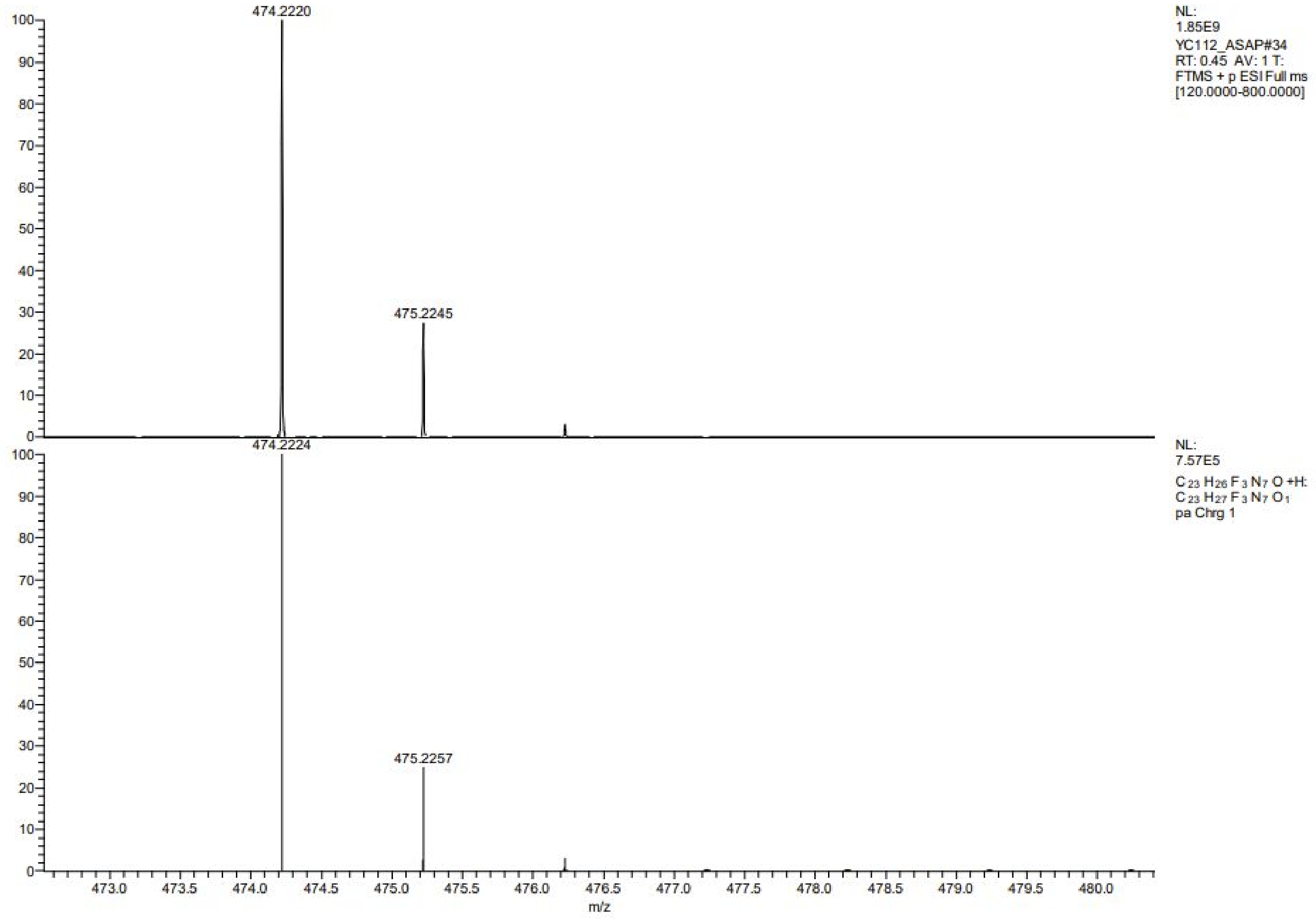

Figure S16-4: HRMS spectrum of Compound **18.**

2-(2,4-difluorophenyl)-1-(4-((5-fluoropyrimidin-2-yl)amino)azepan-1-yl)-3-(1H-1,2,4-triazol-1-yl)propan-2-ol (20)

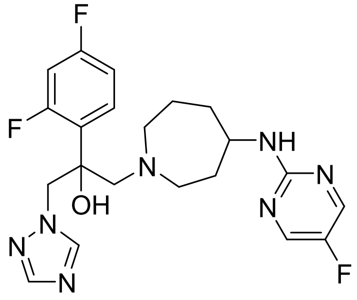

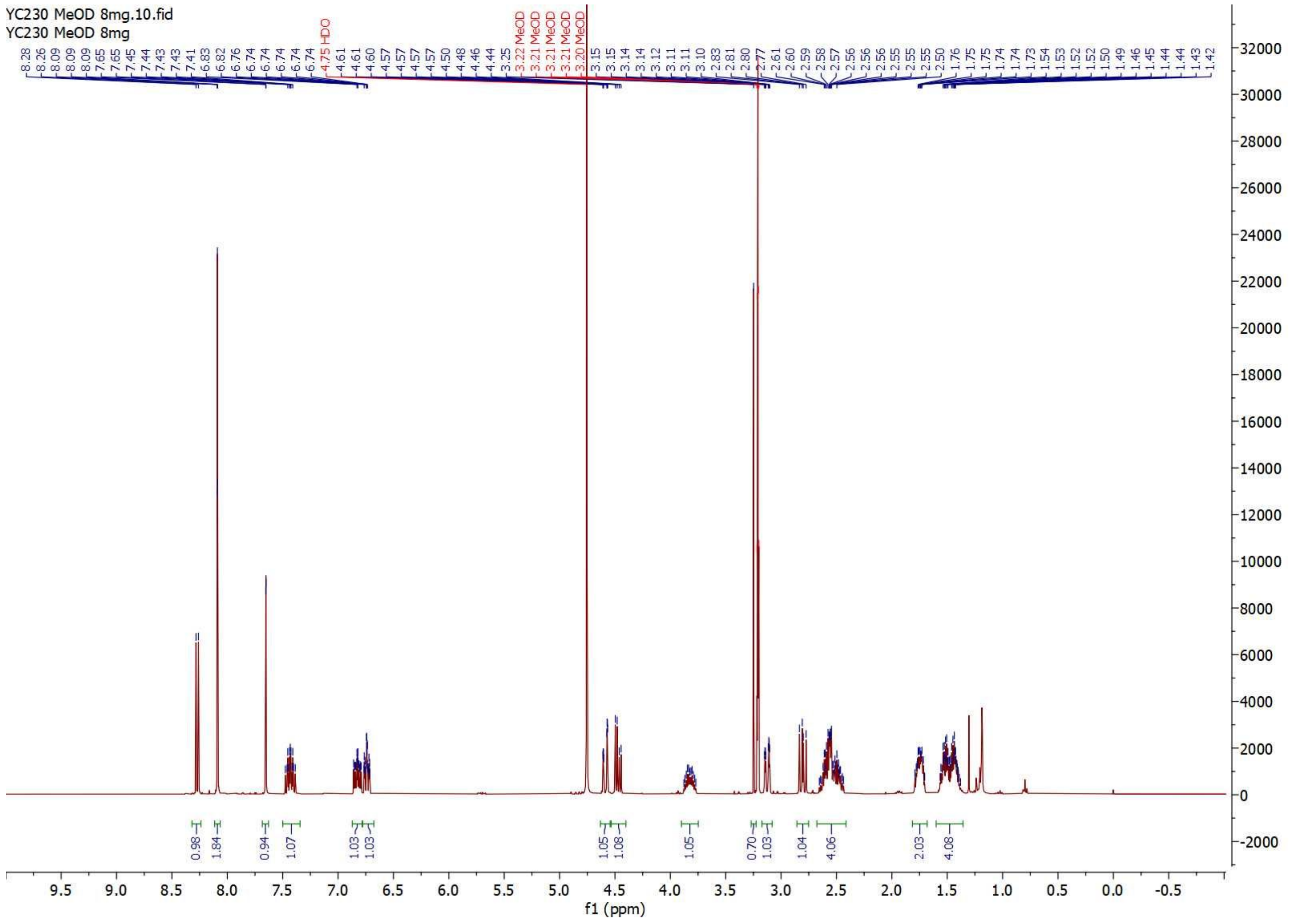

Figure S17-1: Proton ^{1}H NMR spectrum of Compound **20.**

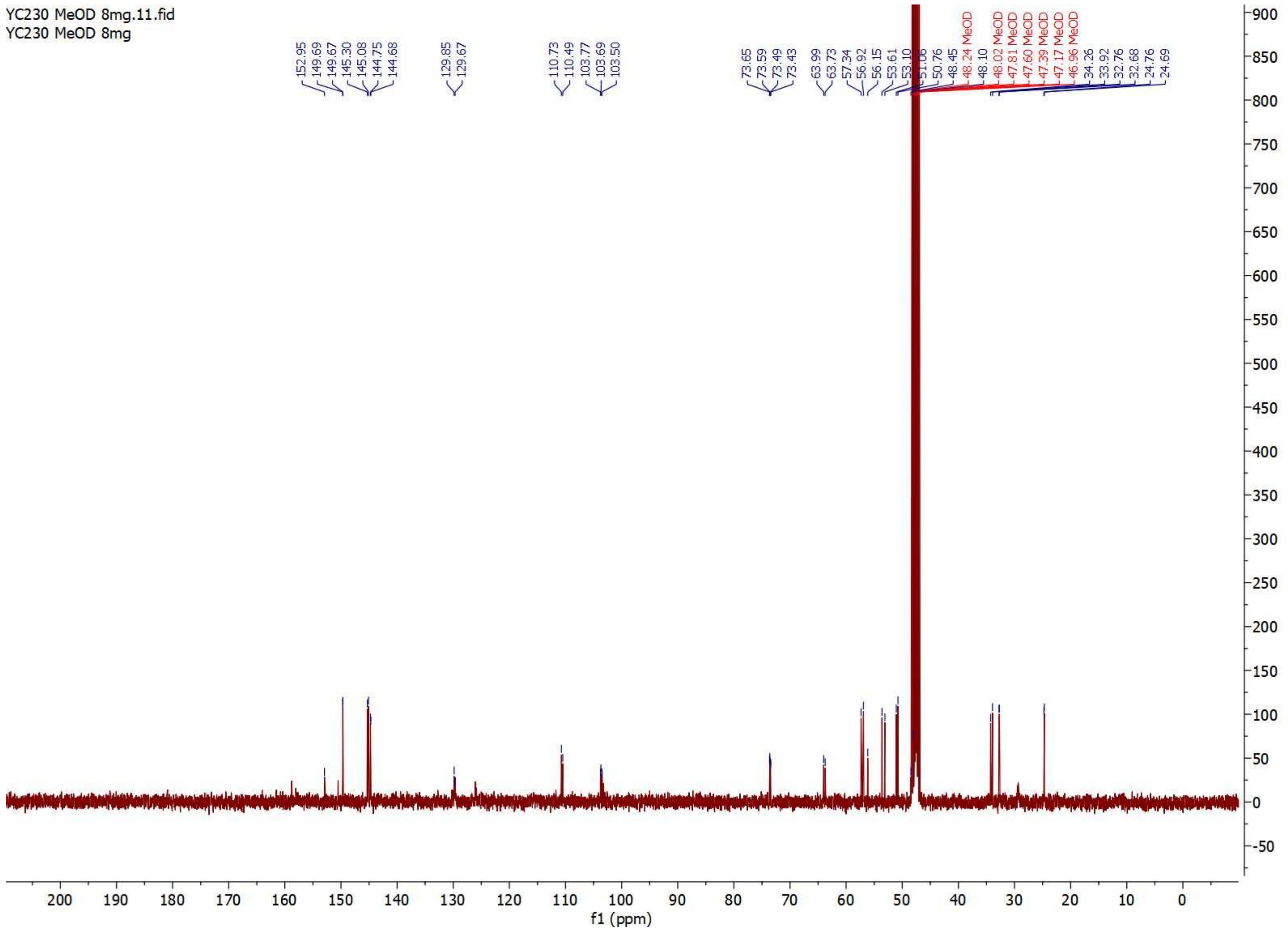

Figure S17-2: Carbon ^{13}C NMR spectrum of Compound **20.**

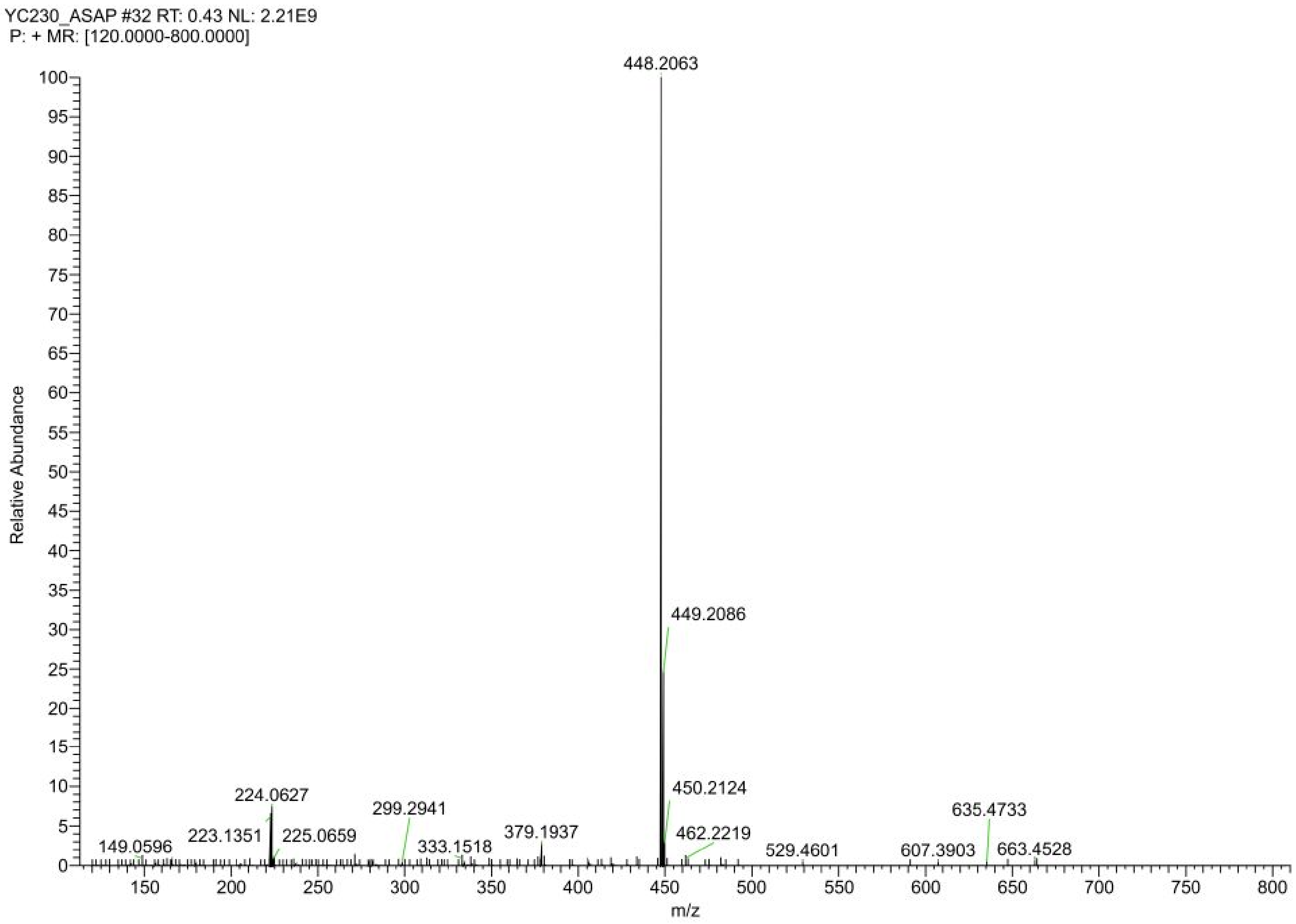

Figure S17-3: HRMS spectrum of Compound **20.**

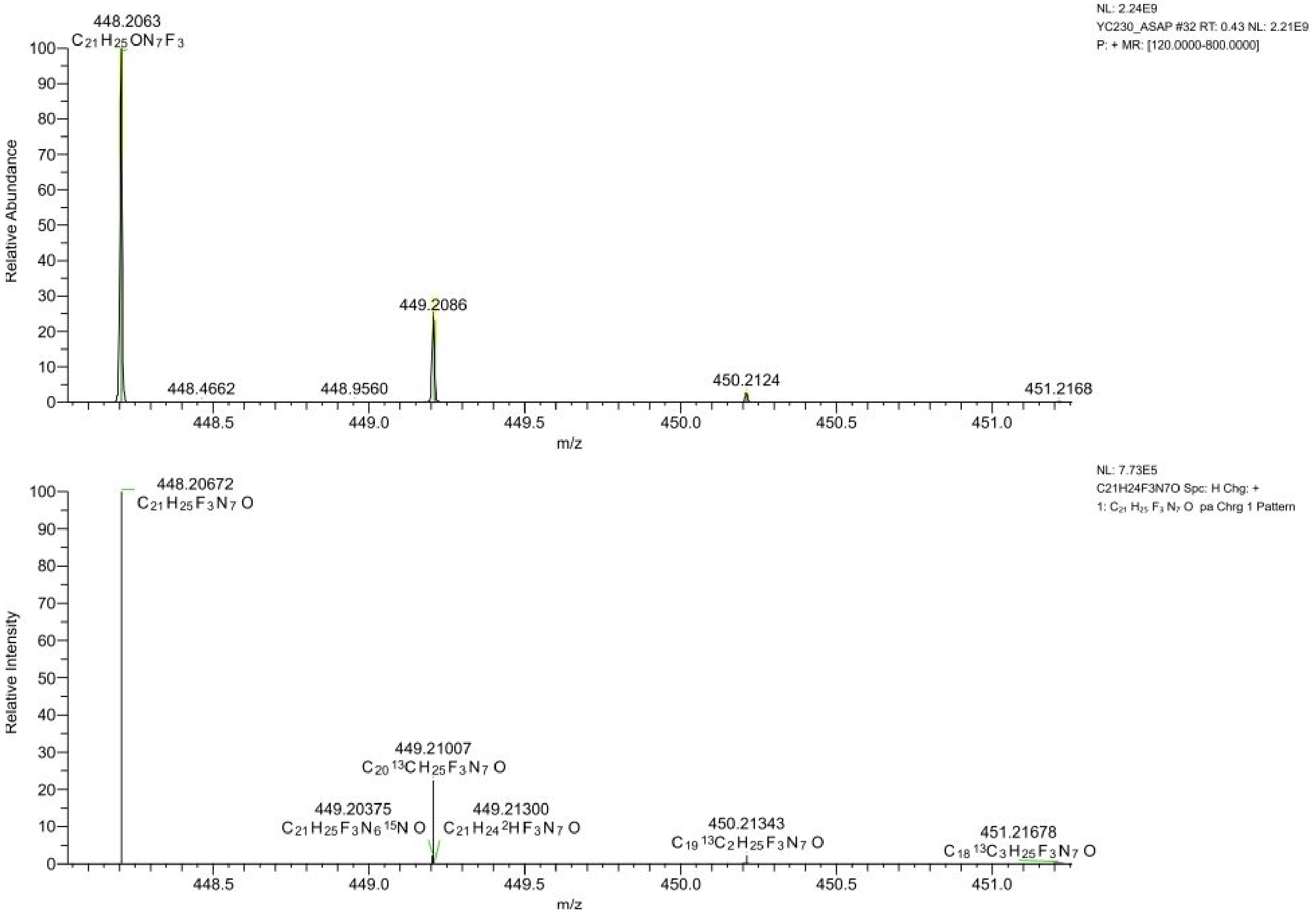

Figure S17-4: HRMS spectrum of Compound **20.**

2-(2,4-Difluorophenyl)-1-(4-(5-fluoropyrimidin-2-yl)-1,4-diazepan-1-yl)-3-(1H-1,2,4-triazol-1-yl)propan-2-ol (21)

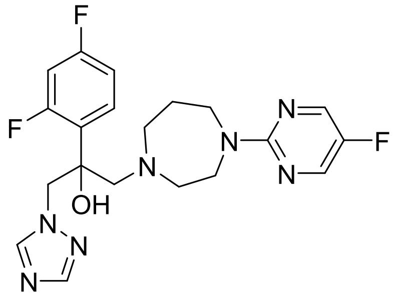

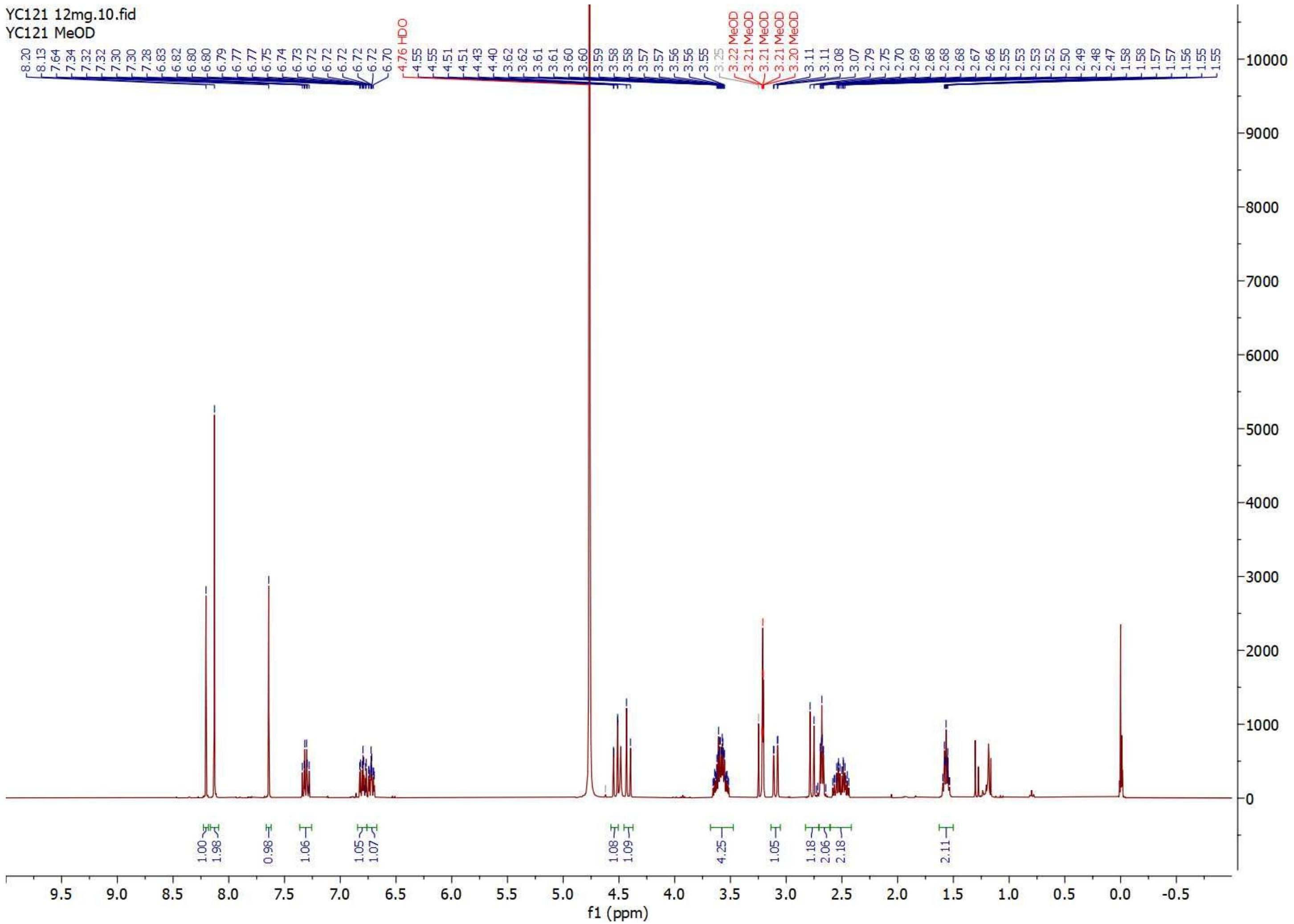

Figure S18-1: Proton ^{1}H NMR spectrum of Compound **21.**

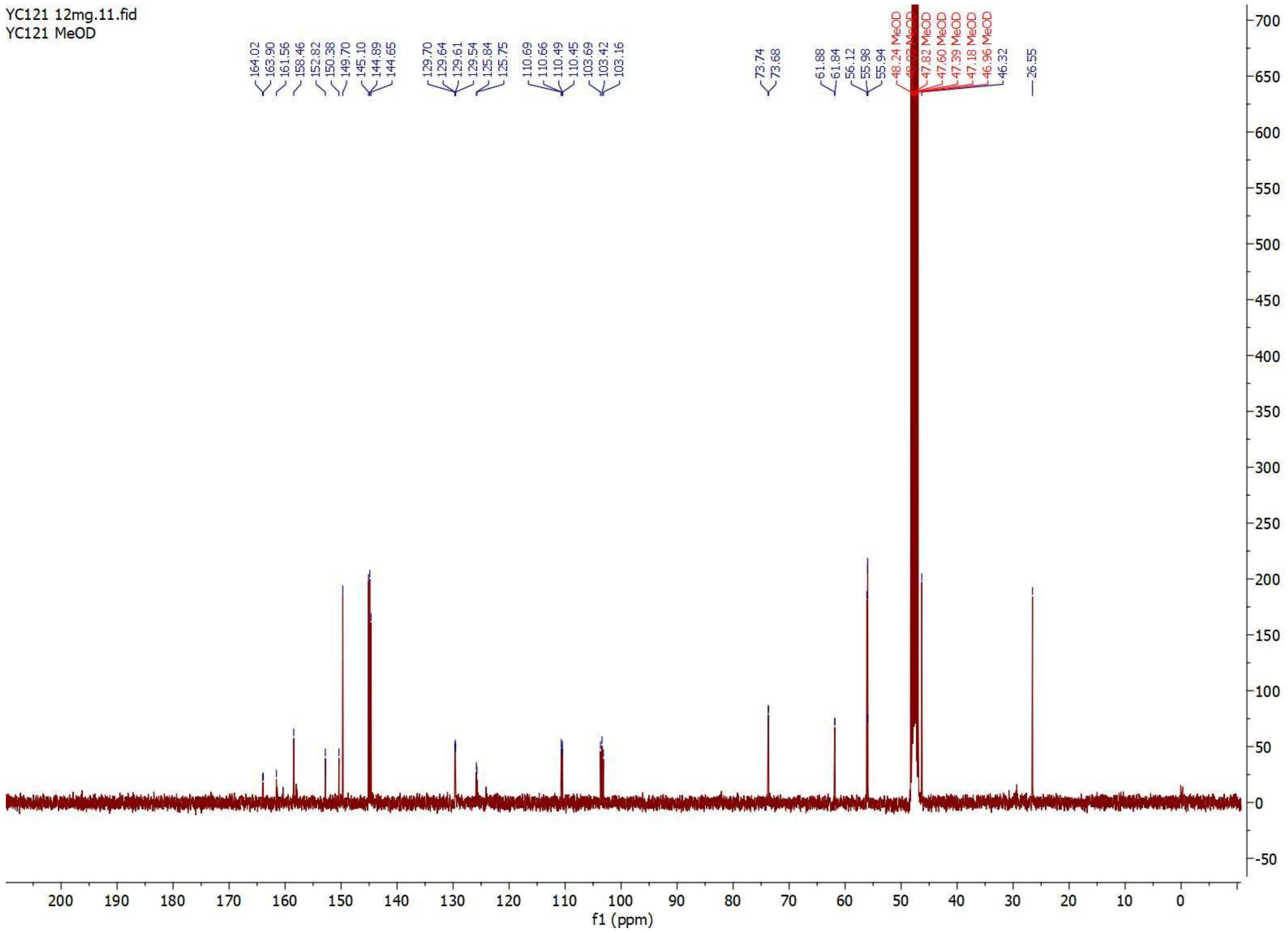

Figure S18-2: Carbon ^{13}C NMR spectrum of Compound **21.**

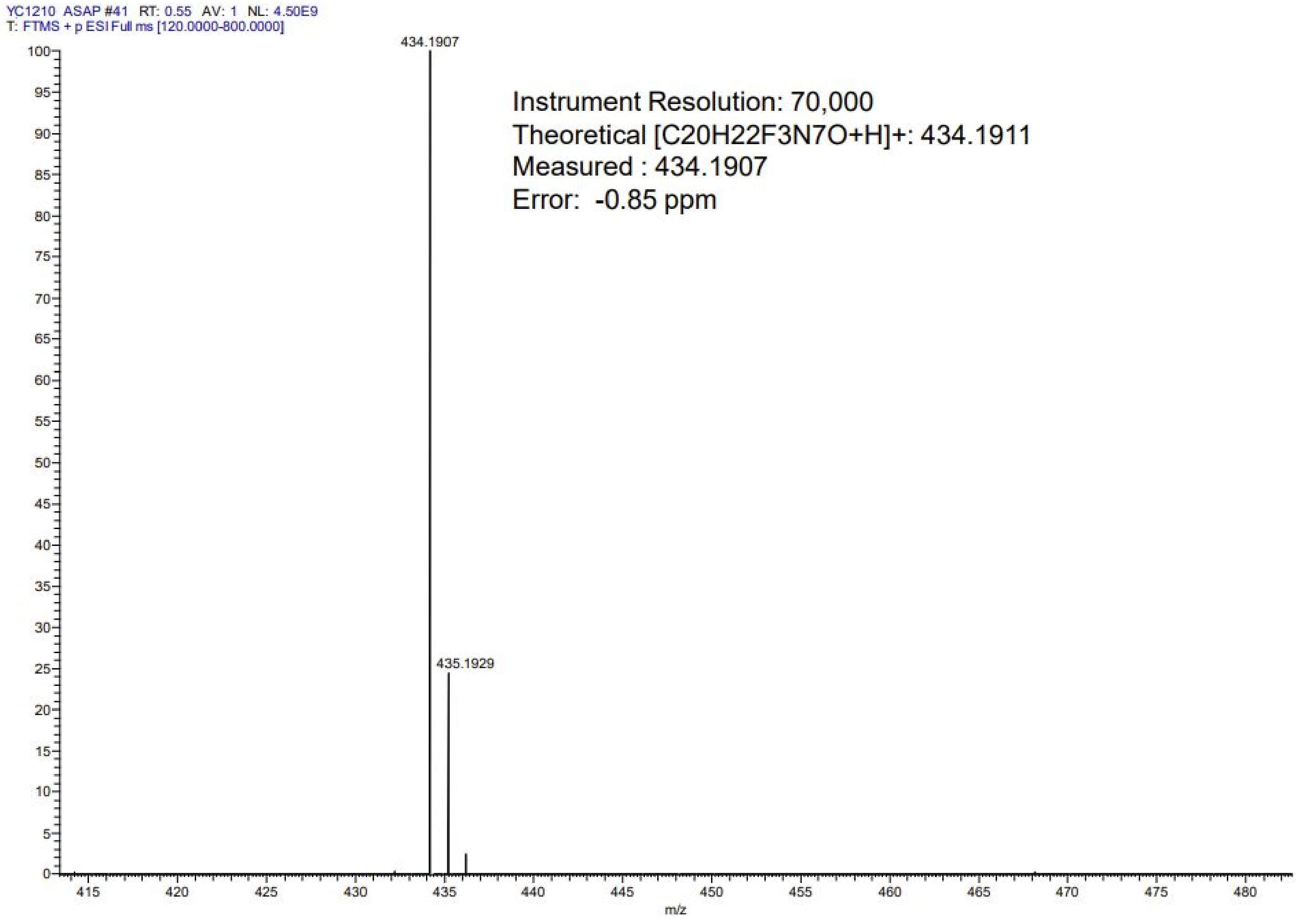

Figure S18-3: HRMS spectrum of Compound **21.**

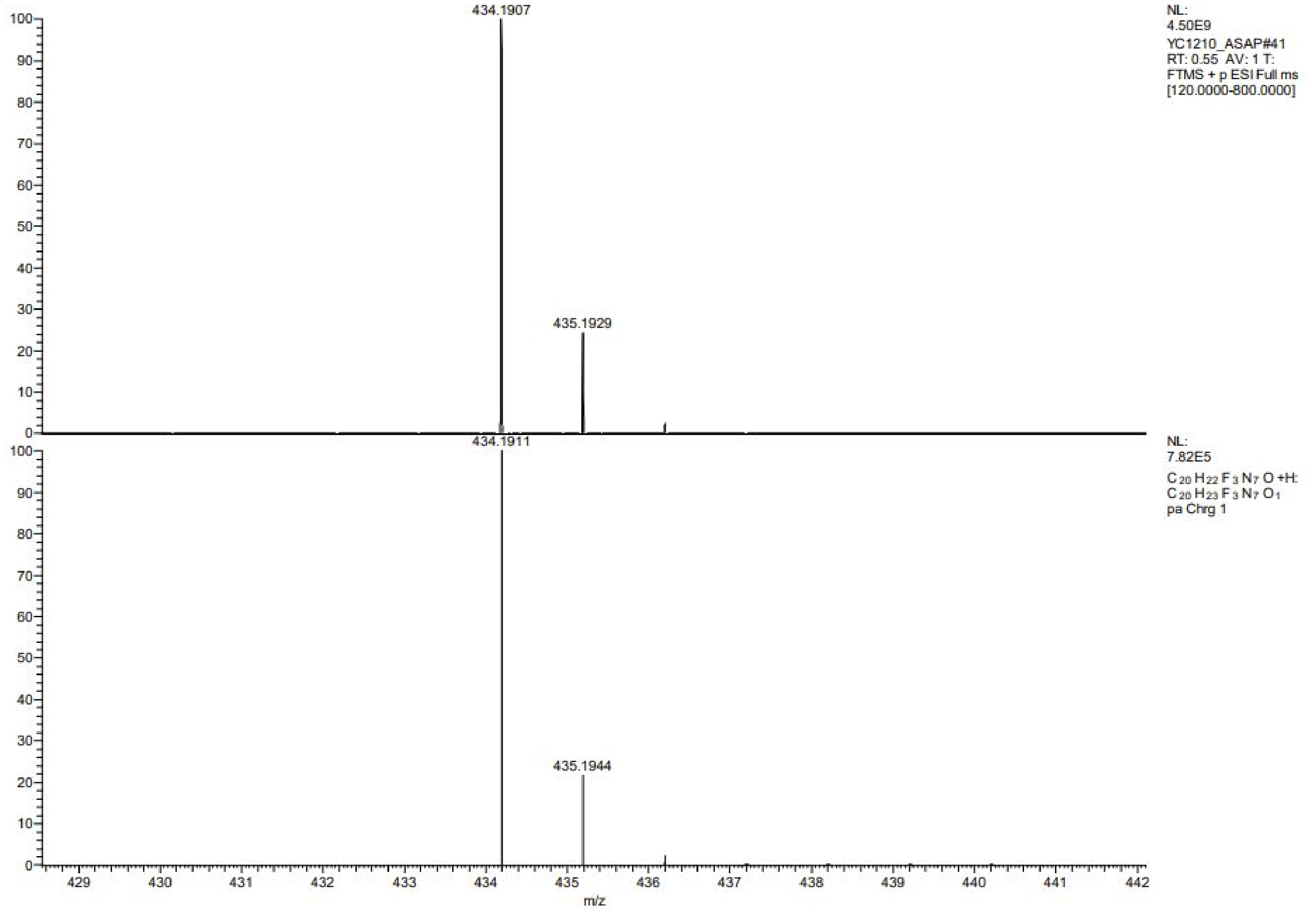

Figure S18-4: HRMS spectrum of Compound **21.**

Representative Example of HPLC Profile of Some Compounds

Note: first peak is solvent front.

Compound 7

Agilent Technologies

ID:	YC013 1mM DMSO Stock			File:	20210826_YC013 1mM DMSO Stock_5730.D
Date	8/26/2021 5:19:05 PM	Vial:	P1-B-04	User:	Admin

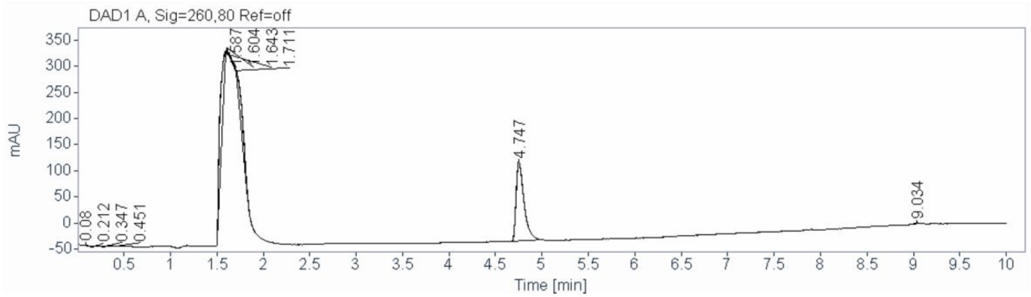

Compound 8

ID: YC096 P File: 20190919_YC096 P_0228.D

Date 9/19/2019 2:54:16 PM Vial: P2-C-04 User: Admin

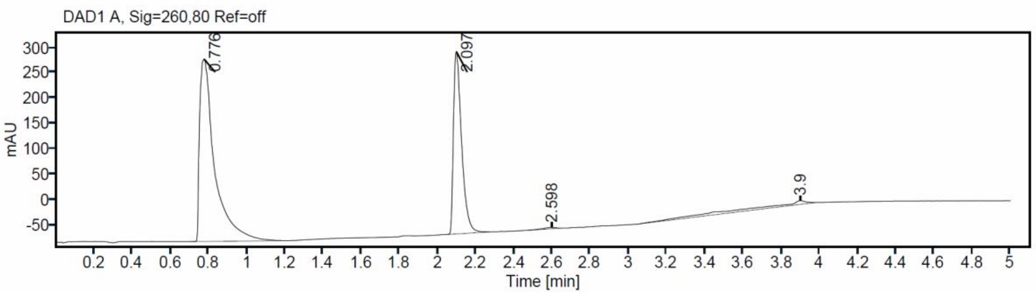

Compound 9

Agilent Technologies

ID: YC099 P File: 20191112_YC099 P_1028.D

Date 11/12/2019 1:48:16 PM Vial: P1-F-06 User: Admin

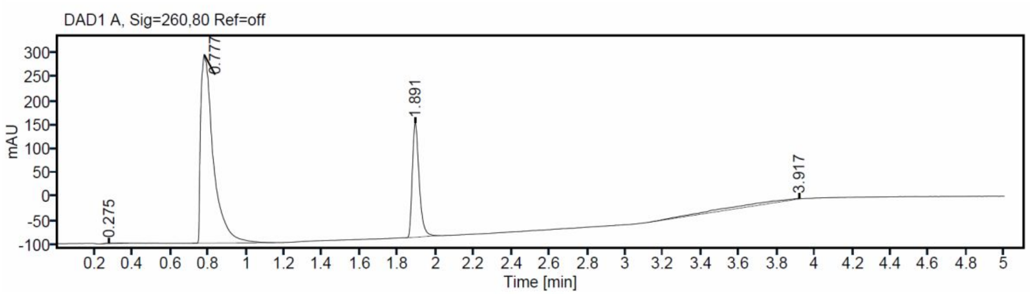

Chiral HPLC Analysis of Compound 7

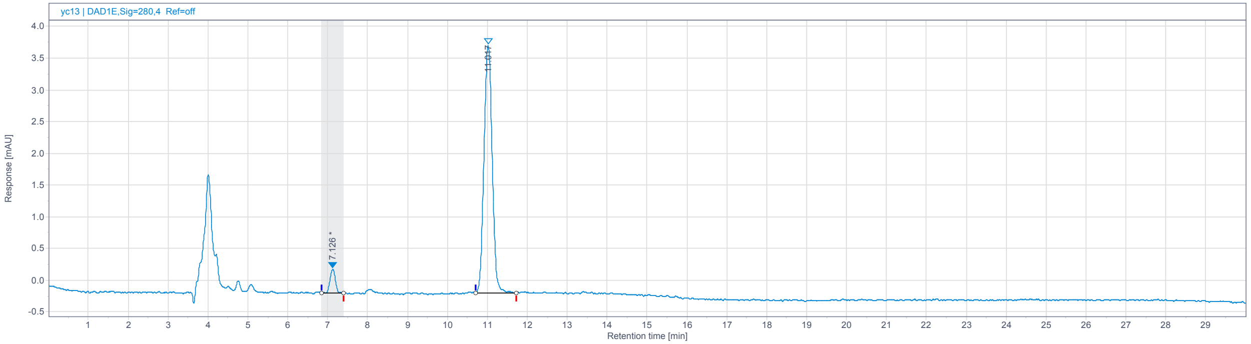

Figure S19a. Rp chiral HPLC analysis of compound **7 (YC-13)** showing a single peak at RT 11.01 minute. HPLC method: MeCN/H_2O/formic acid (50/50/0.1 %), isocratic run for 30 mins, flow rate 0.8 ml min^{-1}, monitored at λ = 280 nm.

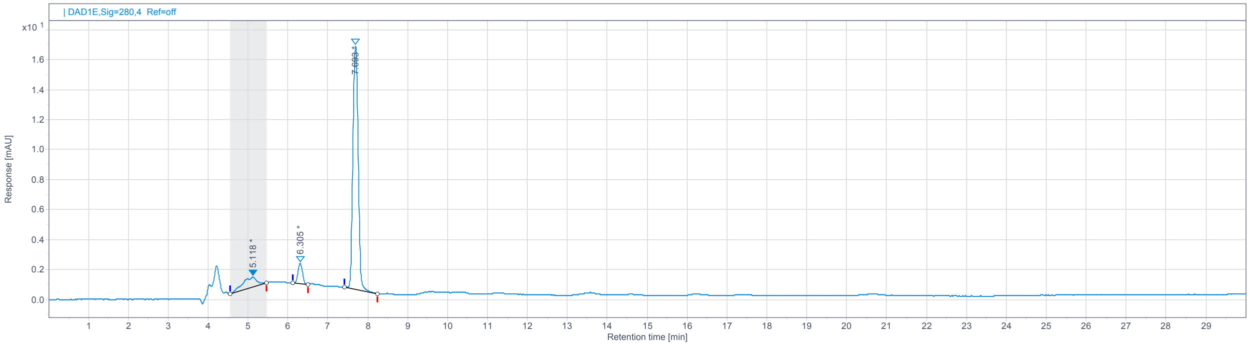

Figure S19b. Rp HPLC analysis of isolated compound **7 (YC-13)** showing a single peak, other than solvent peaks, at RT 7.69 minute. Method: MeCN/H_2O/formic acid(60/40/0.1 %), isocratic run for 30 mins, flow rate 0.8 ml min^{-1}, monitored at λ = 280 nm.

Method: 70:30 ACN: Water, 30 mins

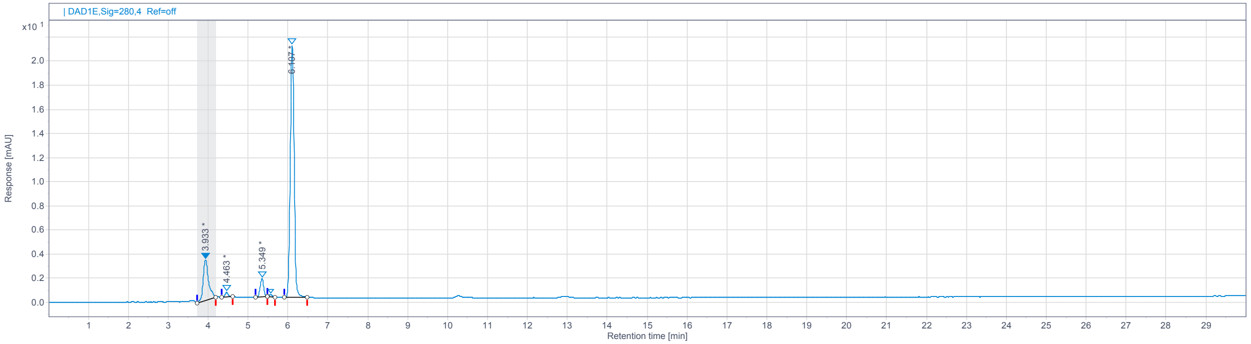

Figure S19c. Rp HPLC analysis of isolated compound **7 (YC-13)** showing a single peak, other than solvent peaks, at RT 6.1 minute. Method: MeCN/H_2O/formic acid (70/30/0.1 %), isocratic run for 30 mins, flow rate 0.8 ml min^{-1}, monitored at λ = 280 nm.

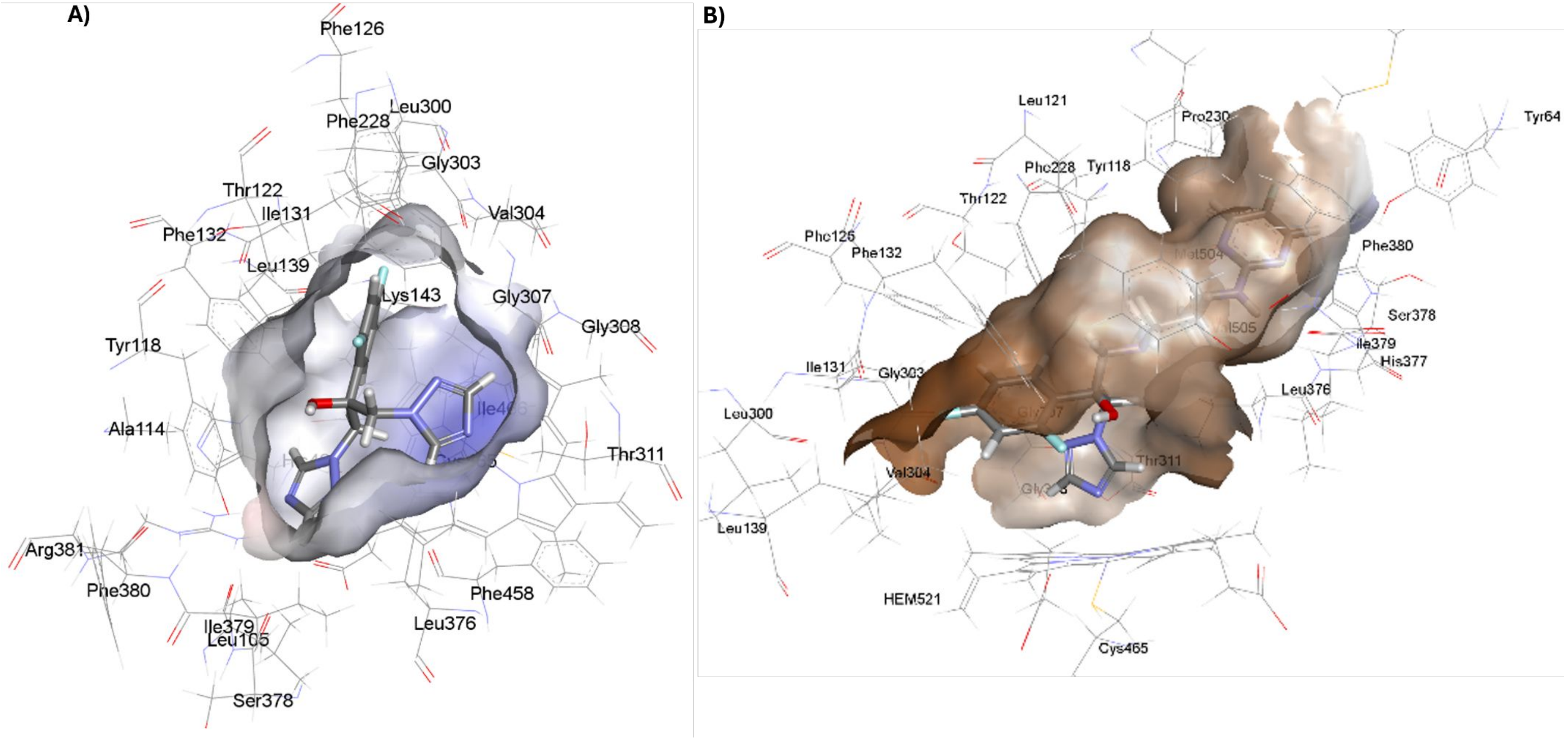

Figure S20: Differences in 3D binding interactions observed for fluconazole (A), and compound **7** (B) with lanosterol 14α-demethylase (LDM) enzyme in *C. auris*. (PDB ID 5TZ1 was used as a template for homology modelling).

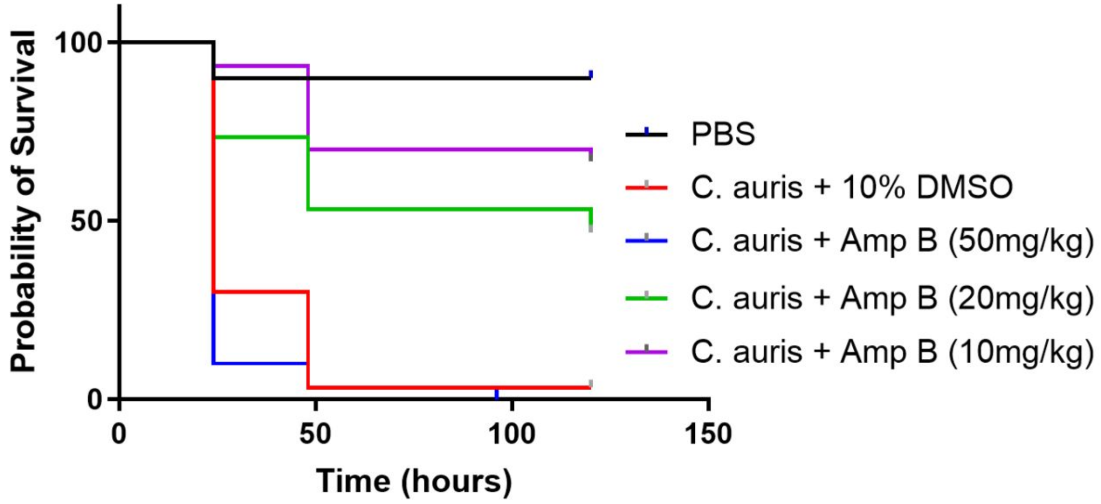

Figure S21. Efficacy of amphotericin B (Amp B) against *C. auris* TDG1912 infection in *G. mellonella* model.

Table S3. Statistical analysis of *G. mellonella* efficacy data.

Treatment	Mantel-Cox		Gehan-Breslow-Wilcoxon	
	**P value**	**Significance**	**P value**	**Significance**
1912 + Fluc 50	0.0016	**	0.0002	***
1912 + Fluc 20	0.2812	no	0.1672	No
1912 + Fluc 10	0.2953	no	0.1483	No
1912 + YC13 50	<0.0001	****	<0.0001	****
1912 + YC13 20	0.0002	***	<0.0001	****
1912 + YC13 10	0.0109	*	0.0021	**
1912 + amp b 50	0.0849	no	0.0627	No
1912 + amp b 20	<0.0001	****	<0.0001	****
1912 + amp b 10	<0.0001	****	<0.0001	****

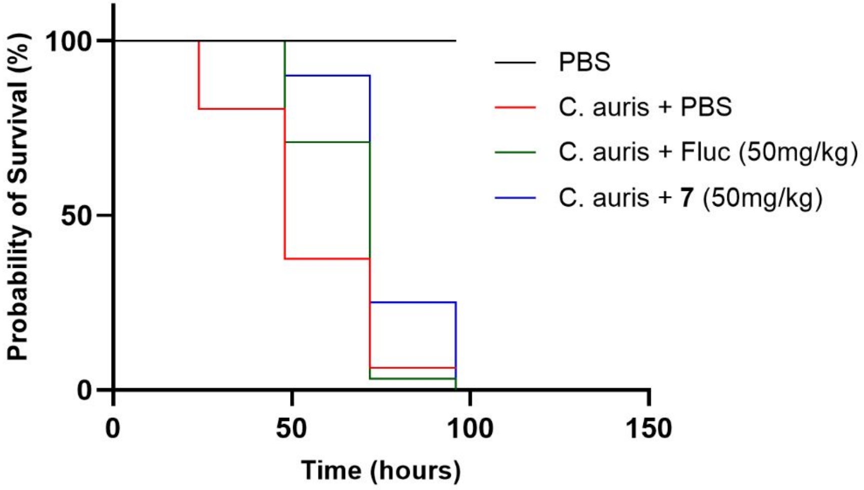

Figure S22. Efficacy of modified azole compound **7** and fluconazole against *C. auris* TDG1912 infection in *D. melanogaster* model at an infection dose of 1 × 10^4 yeast cells per fly.
